# Membrane stabilizer Poloxamer 188 improves yield of primary isolated rat cardiomyocytes without impairing function

**DOI:** 10.14814/phy2.14382

**Published:** 2020-02-28

**Authors:** Teresa L. Czeiszperger, Madison P. Wang, Charles S. Chung

**Affiliations:** ^1^ Department of Physiology Wayne State University Detroit MI USA

**Keywords:** calcium, cardiomyocyte, P188 (Poloxamer 188), sarcomere

## Abstract

Intact cardiomyocytes are used to investigate cardiac contractility and evaluate the efficacy of new therapeutic compounds. Primary enzymatic isolation of adult rodent cardiomyocytes has limitations, including low cardiomyocyte survival, which is likely due to ischemic conditions and/or membrane damage. The addition of Poloxamer 188 (P188) has been used to reduce ischemia‐ and membrane‐related damage in ischemia–reperfusion and muscular dystrophy studies. P188 stabilizes membranes, reducing cell death. Cardiomyocytes were isolated from rats, under three conditions: (1) using standard isolation solutions, (2) with P188 added during cannulation (ischemic event), and (3) with P188 added during cannulation, enzymatic digestion, and trituration. Cell survival was assessed by quantifying the number of rod‐shaped versus contracted cells on the day of isolation and up to 3 days post‐isolation. Adding P188 only during cannulation yielded improved survival on the day of isolation. Little difference in survival was seen among the three conditions in the days post‐isolation. Cardiomyocyte function was assessed by measuring calcium transients and unloaded sarcomere lengths for up to 2 days post‐isolation. P188 did not consistently alter calcium handling or sarcomere shortening in the isolated cardiomyocytes. We conclude that the addition of P188 to the cannulation (e.g., wash) of the isolated heart may improve initial survival of cardiomyocytes upon primary enzymatic isolation.

## Graphical abstract

Primary isolated cardiomyocytes are valuable tools for cardiovascular and pharmacological research. We show that adding Poloxamer 188 can improve initial yield of myocytes, which may help reduce animal models in the long term.

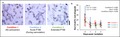

## 1 INTRODUCTION

Isolated, intact cardiomyocytes are an important tool to investigate molecular mechanisms underlying cardiac contractility and to screen novel therapeutic compounds (Aronson & Krum, 2012; Campbell, Haynes, Kelsey Snapp, Nava, & Campbell, 2013; King et al., 2011; Malik & Morgan, 2011). While primary isolation of adult (typically rodent, but also canine, human, and avian) myocytes is common (Ackers‐Johnson et al., 2016; King et al., 2011; Louch, Sheehan, & Wolska, 2011; Makwana et al., 2010), limitations to their use exist. Previously, we and others assessed simple, low‐cost methods to enable the use of myocytes for several days following primary isolation without the use of culture by inhibiting cell membrane blebbing (Abi‐Gerges et al., 2013; Chung, Mechas, & Campbell, 2015). However, cardiomyocyte survival remains a hurdle in reducing the use of animal models and the ability to use a single biological replicate in multiple conditions.

**Figure 1:**
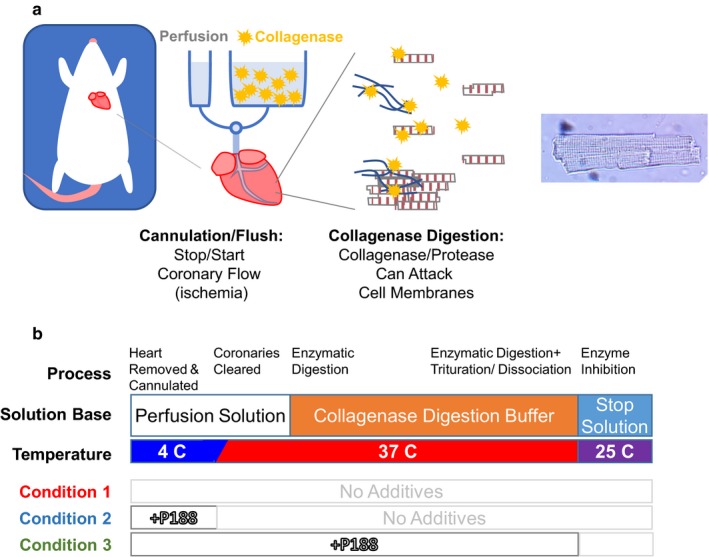
Experimental considerations. (a) Graphical timeline of isolation shows that there are two areas of high‐risk damage to cardiomyocytes during primary isolation. (b) A typical isolation process, including its solutions and temperatures, are outlined. The addition of Poloxamer 188 (P188) is noted during cannulation and wash (Condition 2) or through dissociation (Condition 3)

The traditional method to isolate myocytes includes excision of a heart and Langendorff perfusion before enzymatic digestion of collagen and cellular adhesions (Louch et al., 2011). Attempts to isolate myocytes have existed for many years (Cavanaugh, 1955) and development of Langendorff protocols (Berry, Friend, & Scheuer, 1970; Powell & Twist, 1976; Vahouny, Wei, Starkweather, & Davis, 1970) accelerated after the publication of work by Kono (1969). While protocols avoiding Langendorff perfusion have been proposed (Ackers‐Johnson et al., 2016), we re‐evaluated this more traditional method. We speculate that there are two time points at which myocytes are most vulnerable to damage during this protocol (Figure 1a). First, the excision of the heart and subsequent Langendorff perfusion creates a brief ischemic condition. Even though high potassium solutions and crossbridge inhibitors are used, the brief ischemia–reperfusion may create a risk of increased apoptosis. Second, the enzyme mixtures not only include collagenases but also proteases. Combined with a trituration step to increase dissociation, this period may induce significant damage on cellular membranes.

One compound that may counteract both of these issues is Poloxamer 188 (P188). P188 is sometimes referred to as a membrane sealant and has been trialed in both the treatment of ischemia and in membrane stabilization. In myocardial ischemia–reperfusion studies, P188 preserved larger areas of viable myocardium (Bartos et al., 2016). We hypothesized that inclusion of P188 could improve the survival of myocytes undergoing primary isolation via a Langendorff method. Furthermore, we investigated whether any benefit from P188 survival is maintained for multiple days and/or if functional changes were apparent due to the use of P188 during the isolation process. We chose two conditions to test this in hopes of reducing the frequency of isolations in research laboratories.

## 2 METHODS

### 2.1 Animals

In all, 18 female Sprague‐Dawley rats (3.6 ± 0.7 months of age; Charles River) were used in this study. All animal use was approved by the Institutional Animal Use and Care Committee of Wayne State University.

### 2.2 Cardiomyocyte Isolation

Cardiomyocytes were isolated as previously described (Campbell et al., 2013; Chung & Campbell, 2013; Chung et al., 2015), except without pretreatment of IP heparin. Rats were anesthetized using 3% isoflurane and afterwards killed by exsanguination. The excised hearts were placed in a 4°C, oxygenated (10–15 ppm) perfusion solution (in mmol/L: 113 NaCl, 4.7 KCl, 0.6 KH_2_PO_4_, 1.2 MgSO_4_, 12 NaHCO_3_, 10 KHCO_3_, 10 4‐(2‐hydroxyethyl)‐1‐piperazineethanesulfonic acid [HEPES], 30 Taurine, 5.5 glucose, and 10 2,3‐butanedione monoxime [BDM]; pH ~7.3). Subsequently, the hearts were immediately cannulated and flushed with 5 ml of cold perfusion solution. The hearts were then mounted on a gravity‐fed Langendorff perfusion stage and perfused with warm perfusion solution (37°C, 90 mmHg pressure) until they were completely cleared of blood (until warm perfusion dripped clear). The heart was then perfused with warm (37°C) digestion solution (perfusion solution containing 2 µmol/L CaCl_2_ and ~235 U/ml Collagenase Type II, Worthington Biochemical). Hearts were enzymatically digested until they were soft and pale pink (9 ± 1 min) and then removed from the cannula. The digestion solution was bubbled with 95% oxygen and 5% carbon dioxide throughout the enzymatic digestion period. The heart was removed, and the ventricles were manually dissociated by trituration in the digestion solution. The cells were then pipetted into individual 1.5 ml Eppendorf tubes after being filtered through a nylon mesh (300 micron opening) to prevent non‐separated cells, remaining vasculature, or excess matrix from entering the tube. These tubes were also filled with a stop solution (perfusion solution with 10% fetal bovine serum (FBS) and 12 µmol/L CaCl_2_) and then mixed. The cells were allowed to settle by gravity to a pellet for 5 min. The supernatant above the pelleted cells was then drawn off and replaced with a second enzyme stop solution (perfusion solution with 5% FBS and 12 µmol/L CaCl_2_), cells mixed again, and then allowed to settle again for 5 min. Finally, the extracellular calcium was raised to 0.5 mmol/L using a four‐step calcium ladder.

There were three groups tested during this study: (1) using control isolation solutions as described above, (2) with 150 µmol/L Poloxamer 188 (#15759, Sigma Aldrich) added acutely only to the perfusion solution during cannulation, and (3) with prolonged addition of 150 µmol/L P188 to both the perfusion and digestion but not stop solutions. The conditions are outlined in Figure 1b. P188 was mixed into the solutions slowly to minimize bubbles at the surface. Six rats were used for each condition, with the order varied throughout the study.

### 2.3 Preparation and measurement of isolated myocytes

In all, 18 tubes of cells were initially made on the day of each isolation. Three tubes of cells were selected at random each day. On the day of isolation, a five‐step calcium ladder was performed to raise the extracellular calcium concentration to 1 mmol/L for three tubes, and the cells were allowed to acclimate for ~1 hr. For the remaining tubes, the calcium concentration was raised to 0.5 mmol/L in four steps and placed in a storage solution (perfusion solution with 5 mmol/L BDM and 0.5 mmol/L CaCl_2_; Chung et al., 2015). The storage solution was changed in all tubes every day, and with glucose added and freshly oxygenated as above. For each day cells were used, the storage solution was removed or diluted out of the stored tubes via three 20‐min incubations in BDM‐free perfusion solution, and the extracellular calcium concentration was raised to 1 mmol/L. The cells acclimated to calcium and room temperatures for at least 1 hr before functional measurements were performed.

The survival of myocytes was first assessed as previously described (Chung et al., 2015). Briefly, ~15 µl of lightly pelleted cells was taken from each tube and pipetted on a microscope slide using a large‐bore pipette tip. Photos of the cells were taken under a microscope (IX73, Olympus) using a 4× and a 40× objective and a 10‐megapixel camera (MU1003, AmScope) mounted in the microscope's eyepiece. A minimum of three images were acquired from each tube each day until 3 days post‐isolation.

Subsequently, functional measurements were taken. These were performed in an oxygenated (10–15 ppm) Tyrode's solution (in mmol/L: 140 NaCl, 5.4 KCl, 1.8 CaCl_2_, 1 MgCl_2_, 10 HEPES, and 10 glucose), pH ~7.3 similar to what was previously described (Campbell et al., 2013; Chung & Campbell, 2013; Chung et al., 2015). Briefly, a 40 µL aliquot of cells acclimated to 1 mmol/L calcium was added to 230 µl of Tyrode's solution with 0.02% pluronic acid and 2 µmol/L Fura‐2AM (Life Technologies). Fura‐2 was allowed to incorporate into the cytosol for 10 min. A volume of 30 µl of the Fura‐loaded cells was then placed in 150 µl of Tyrode's solution to prevent an excess loading of Fura or loading of Fura into organelles. After 5 min, cells were loaded into a chamber filled with ~1.3 ml oxygenated Tyrode's at 25°C. After allowing the cells to settle into the cover glass (a few seconds), cells were continuously paced at 0.5 Hz during a 5‐msec duration bipolar excitation throughout the experiment using an IonOptix Myopacer. Measurements were performed using a commercially available calcium and contractility system and software (IonWizard 6, IonOptix). Sarcomere length profiles were measured using a high‐speed video camera (Myocam‐S). Calcium transients were measured by monitoring Fura‐2 fluorescence at 510 nm using pseudo‐ratio sampling via excitation at 360 and 380 nm (Ionoptix µstep).

Data were acquired in a series of trials. During each trial, the stage was moved to focus the microscope on a single cell, and signals representing sarcomere length and calcium fluorescence emission were acquired while the cell was beating. About 8–10 cells were measured per tube on the day of isolation, and on subsequent days, ~5 cells were measured per tube. When a new tube of cells was introduced to the chamber, it was completely flushed out and filled with fresh Tyrode's solution.

### 2.4 Analysis

Myocytes were counted in IrfanView and/or ImageJ. Cells counted as living (surviving) cells were rod‐shaped with clearly defined edges, had lengths at least twice as long as the width, and typically had a bright/translucent color. Cells marked as dead appeared ovate or circular with blurry, indistinct edges and a dark color. All cells were counted for each image and summed for each tube/trial (three images per tube); each tube was used as a technical replicate. The percent survival of each tube was calculated as the number of living cells divided by the total number of cells counted.

Calcium transients and sarcomere length twitch profiles from each cell were quantified offline using IonWizard 6 software and calculated parameters were averaged over a minimum of 10 beats. The following parameters were calculated from the calcium transients: minimum and amplitude of the calcium fluorescence ratios, time from the pacing stimulus pulse to the peak fluorescence, and the monoexponential time constant of calcium decline (tau). The parameters obtained from the sarcomere length profiles were as follows: diastolic sarcomere length, amplitude of shortening, time from the pacing stimulus pulse to the peak shortening, and the rates of sarcomere length shortening and relengthening. Each myocyte was treated as an independent sample. Software filtering was applied to all data. Data from individual cardiomyocyte twitches were excluded from further analysis if its calcium transient or sarcomere length profile included noise, if the analysis fits were discontinuous, if the IonWizard software did not detect a transient or profile or if data were greater than five standard deviations from the total and condition means. If the software yielded data with gaps, discontinuities, or completely flat transients, the improper transients were excluded as well.

### 2.5 Statistics

Statistical testing was performed in SPSS (version 25, IBM). The generalized linear model function was used to predict a linear response for each dependent variable. The predictive factors were the isolation (P188) condition and days post‐isolation; a full factorial model was used. To determine the impact of P188 on isolation times and parameters, a one‐way ANOVA or unpaired *t* test was used. The Bonferroni adjustment for Multiple Comparisons was used to test for differences between significant factors (post hoc) in the model. A *p* value <.05 was considered statistically significant. Data reported as mean ± *SD*.

## 3 RESULTS

The effect of Poloxamer 188 (P188) on isolation conditions and times was assesed. Digestion time between the three conditions was not different (Condition 1:9.0 ± 1.0 min, Condition 2:9.1 ± 0.7 min, Condition 3:9.2 ± 0.6 min, *F* = 0.067, *p* = .935 by ANOVA). The volume of digestion buffer used was also not statistically different (Condition 1:48 ± 14 ml, Condition 2:54 ± 7 ml, Condition 3:50 ± 10 ml, *F* = 0.457, *p* = .64 by ANOVA). We also observed no difference in flow rate of the two digestion buffers through the Langendorff system without a heart attached (data not shown).

**Figure 2:**
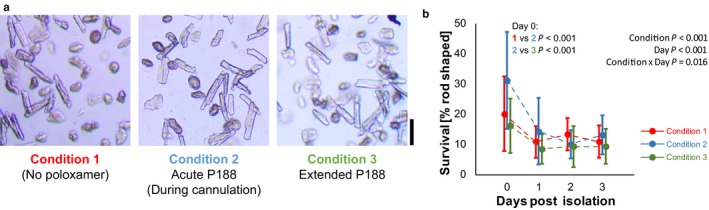
Cardiomyocyte survival. (a) Sample images showing cell survival on the day of isolation (scale bar shows 100 µm). The longer rod‐shaped cells indicate good survival, while the rounded and short cells are no longer viable. (b) Quantification of survival (percent of rod‐shaped cardiomyocytes, mean ± *SD*) on the day of isolation and up to 3 days after; detailed statistics including sample size and post hoc testing are reported in Supplementary Materials

Cardiomyocyte survival was assessed by determining the relative percentage of rod‐shaped cells to the total number of cells counted. The acute addition of P188 only during cannulation (Condition 2) resulted in an increased number of rod‐shaped cells on the day of isolation compared to control conditions (Condition 1) (Figure 2). Cell survival (% rod‐shaped cells) was not different between prolonged P188 addition (Condition 3) and control conditions. P188 did not impact cell survival after 1–3 days of storage at 4°C with 0.5 mmol/L BDM.

**Figure 3:**
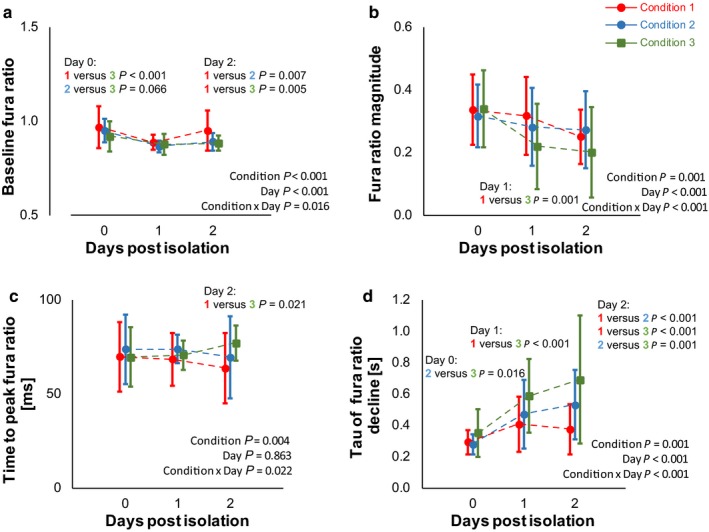
Cardiomyocyte calcium handling. (a) Diastolic (baseline) Fura ratio, (b) Fura ratio magnitude, (c) time to peak Fura magnitude, and (d) Fura recovery rate are shown. The addition of P188 during cannulation (Condition 2) did not significantly alter the magnitude of calcium in the cell. In contrast, prolonged exposure to P188 (Condition 3) caused changes in both Fura magnitude and recovery. Data shown as mean ± *SD*, detailed statistics including sample size and post hoc testing are reported in Supplementary Materials

Cardiomyocyte calcium handling is summarized in Figure 3; detailed statistics (including post hoc tests) are included in Supplementary Data. As shown on Figure 3, (a) baseline Fura ratio, (b) Fura ratio magnitude, (c) time to peak Fura ratio, and (d) tau of Fura decline show significant interaction by condition and days post‐isolation. Additionally, the time to peak Fura ratio showed a similar interaction, whereas the absolute peak ration showed only main (Condition and Day) effects. Importantly, nearly all of the significant differences were between Condition 3 (prolonged P188) and Condition 1 (control). Condition 2 (P188 acutely only during cannulation) only differed from Condition 1 2 days after isolation for baseline Fura Ratio and the tau of Fura decline from peak.

**Figure 4:**
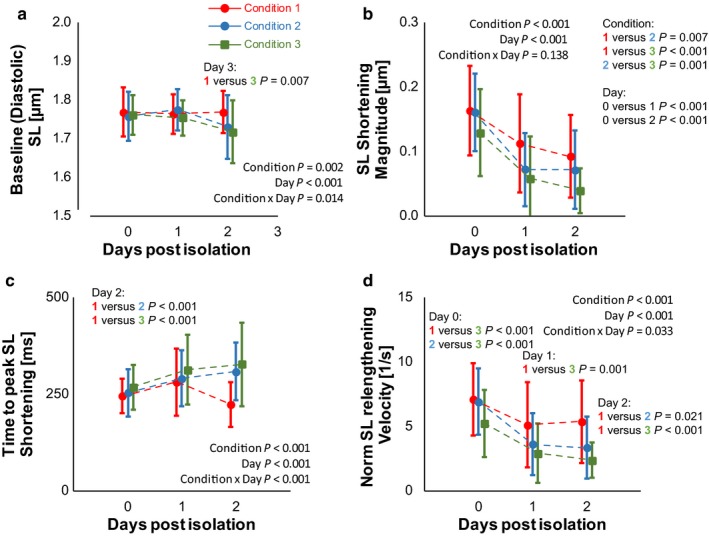
Cardiomyocyte sarcomere length (SL) function. (a) Diastolic (baseline) sarcomere length, (b) sarcomere length shortening magnitude, (c) time to peak SL shortening, and (d) normalized relengthening velocity are shown. The addition of P188 did not significantly modify sarcomere length kinetics on the day of isolation. Data shown as mean ± *SD*; detailed statistics including sample size and post hoc testing are reported in Supplementary Materials

Cardiomyocyte contractile function is summarized in Figure 4; detailed statistics (including post hoc tests) are included in Supplementary Data. Baseline (diastolic) sarcomere length showed a condition by day interaction (Figure 4a) while sarcomere length shortening magnitude did not show an interaction, but did show differences between conditions and days (Figure 4b). For diastolic sarcomere length, only Condition 3 was statistically different from Condition 1 and only 2 days post‐isolation. Shortening magnitude declined after storage (days 1 and 2 post‐isolation), and both Condition 2 and Condition 3 showed statistically lower shortening than Condition 1 on those days. However, Condition 2 was nearly identical to Condition 1 on the day of isolation. Time to peak shortening (Figure 4c) and the shortening velocities showed a condition and day interaction. For time to peak and shortening velocity, only Condition 3 showed statistical differences to Condition 1. Raw relengthening (µm/s) only showed main effects (supplement), but when normalized to the shortening magnitude (Figure 4c) an interaction was observed. Condition 3 again showed the most prominent differences, with Condition 2 only differing from Condition 1 2 days post‐isolation.

## 4 DISCUSSION

Mechanistic studies of cardiac function and drug discovery often utilize intact cardiomyocytes (Aronson & Krum, 2012; King et al., 2011; Malik & Morgan, 2011). Previously, we and others investigated the use of isolated myocytes over several days post‐isolation (Abi‐Gerges et al., 2013; Chung et al., 2015; Kivisto et al., 1995). The current study sought to investigate cell survival of the myocytes during the isolation process and how that might impact cell survival and function over multiple days.

### 4.1 Cardiomyocyte morphology (survival)

Cardiomyocyte survival after isolation has been a concern since myocyte isolation techniques were developed (Cavanaugh, 1955; Kivisto et al., 1995; Kono, 1969; Vahouny et al., 1970) and is reported in a wide range (from ~15% to 80%) (Abi‐Gerges et al., 2013; Ackers‐Johnson et al., 2016; Kivisto et al., 1995). Survival is often reported as a percentage of rod‐shaped cells, as we do in this study. In the current study, the initial cardiomyocyte survival was approximately 22.8% across all groups, which is on the lower end. It is possible that this value is low because, unlike previous studies (Chung et al., 2015; Louch et al., 2011), this study did not use heparin prior to isolation. The lack of anti‐coagulation may have caused micro‐emboli in the coronary arteries causing more detrimental conditions than standard protocols, or more general problems due to a lack of calcium chelation (Ackers‐Johnson et al., 2016).

Adding 150 µmol/L Poloxamer 188 (P188) acutely during cannulation only (Condition 2) significantly increased the survival of cardiomyocytes on the day of isolation compared to the normal condition, increasing survival to 31.2%. This suggests that P188 may be protective during the isolation process. However, excessive exposure may be detrimental as we observed that including 150 µmol/L P188 during both the cannulation and digestion (Condition 3) did not improve survival upon isolation.

Initially, this study also sought to improve the survival of cells stored for functional use in subsequent days post‐isolation. Unfortunately, the current study showed no differences in survival in the days after isolation. This result is likely due to the mechanism of action and/or storage conditions. Cell survival past isolation is typically improved with the use of myosin ATPase inhibitors (Abi‐Gerges et al., 2013; Chung & Campbell, 2013; Kivisto et al., 1995). While P188 may protect mitochondria during ischemia–reperfusion or the membrane during trituration, it does not modify the myosin ATPase. Thus, P188 likely provides some protection and improvement during the isolation process (as discussed below), but does not improve long‐term survival of the cells. Cells are also stored, not cultured. The storage conditions allow cells to pellet to the bottom of an Eppendorf tube, which reduces access to nutrients and oxygen for some cells.

The improved yield upon the addition of P188 might allow for increased efficiency in experiments. While low‐throughput mechanical experiments may not benefit from the increased yield, the increased number of cells surviving in culture may help reduce the number of animals used in drug discovery experiments. This would also allow for increased sampling from a single biological replicate (source heart) for use with different conditions, also increasing throughput.

### 4.2 Poloxamer mechanism

The modest improvement in cardiomyocyte survival on the day of isolation may be due to known mechanisms of P188 function. Poloxamers are a class of molecules that include pluronic acids with hydrophobic centers and two hydrophilic arms, allowing it to insert into the plasma membrane. The properties of poloxamers are dependent on the composition of their hydrophobic and hydrophilic regions (Adhikari, Goliaei, Tsereteli, & Berkowitz, 2016). Pluronic acids are regularly used to allow membrane transit as is common in the loading of calcium‐sensitive dyes acetoxymethyl (‐AM) esters (Louch et al., 2011).

P188 has previously been used to stabilize plasma or sarcolemmal membranes in both ischemia–reperfusion injury and muscular dystrophy models (Bartos et al., 2016; Townsend et al., 2010; Yasuda et al., 2005). It was hypothesized that P188 blocked cellular markers of apoptosis and necrosis that were present in untreated myocytes. Ischemia–reperfusion injury leads to myocyte membrane instability and swelling, causing intracellular calcium levels to elevate, mitochondrial membrane depolarization, and ultimately cell death. Stabilizing sarcolemma membranes using P188 interrupts this feed forward cycle, preventing apoptosis and necrosis (Martindale & Metzger, 2014). After the plasma membrane heals, a poloxamer molecule likely exits the membrane by diffusion when the extracellular concentration is again lowered.

However, poloxamers can cause physiologic side effects or biophysical problems with membrane function. Clinical trials of P188 to improve ischemia–reperfusion injury and muscular dystrophy have been halted due to renal dysfunction (Maynard et al., 1998). P188 is hypothesized not to be metabolized and is excreted via the kidney in its original form, which is correlated with elevated creatinine levels. On a biophysical level, high levels of poloxamers may also be detrimental to the cells of interest. Specifically, when the concentration of poloxamers increases, the phase stability of the lipid bilayers is affected (Maskarinec, Hannig, Lee, & Lee, 2002). This phase transition reduces the stability of the membrane and causes separation of different components. Thus, P188 can acutely interact with and seal small pores (Adhikari et al., 2016), but higher concentrations may cause larger pores or cell damage.

In this study, we used 150 µmol/L P188 to assess if cell survival would be improved. In previous studies, concentrations as low as 60 µmol/L provided some improvements in function in myocytes from *mdx* mice, but did not restore function as much as 150 µmol/L which was shown to restore both compliance and calcium levels back to levels of their C57BL/10 controls (Yasuda et al., 2005). Concentrations up to 1 mmol/L P188 have been used to improve function of skeletal muscles from mice with muscular dystrophies (Ng, Metzger, Claflin, & Faulkner, 2008), but we used 150 µmol/L to stay within a range more commonly used in the literature. Due to the potential negative effects on the lipid bilayers (Maskarinec et al., 2002), we speculate that a high concentration negatively impacts myocyte survival even with acute exposure. However, the exact dose–response is unclear.

P188 has also been observed to both modify blood viscosity and collagenase activity. The reduction in blood viscosity has been leveraged as a sickle cell anemia treatment and is likely to be based on modifying cell–cell interactions (Adams‐Graves et al., 1997). It is possible that P188 improved our coronary flow by interacting with clotted blood in the coronary arteries. However, we observed no significant change in flow rate or volumes of buffer used, suggesting no change in clotting or viscosity. One alternate mechanism of poor survival in Condition 3 may be due to the effect of P188 on collagenase activity. It was previously shown that P188 concentrations of 1 mg/ml and greater increase the activity of *Clostridium histolyticum* (*C. collagenase*) (Jovanovic, Ermis, Mewaldt, Shi, & Carson, 2012). Our study utilized 1.26 mg/ml, which may have caused increased enzymatic activity that could further damage the myocytes, exacerbating any direct effect of P188 on the lipid bilayers.

### 4.3 Functional measurements

We also evaluated if P188 would modify the function of cardiomyocytes since dysfunction would counteract any benefit in cell survival. P188‐induced dysfunction would be especially concerning since cell shortening of unloaded intact cardiomyocytes is used for drug discovery (Aronson & Krum, 2012; Malik & Morgan, 2011). For this study, we evaluated calcium handling and sarcomere length shortening of unloaded intact cardiomyocytes.

This study showed very few significant changes of myocyte function. In regard to calcium handling, cardiomyocytes exposed to prolonged P188 (Condition 3) showed the most different calcium handling from the Condition 1 control group. The function of myocytes from the acute P188 exposure group (Condition 2) only differed from the control group after storage. The most apparent change is the slowed tau of the decline of the Fura Ratio. For Condition 3, the Fura Ratio decline is exacerbated compared to the other groups, possibly indicating that the prolonged exposure to P188 caused problems in the sarcoplasmic reticulum or cell membranes, as described above. Condition 2 diverged from Condition 1 2 days post‐isolation. While this might indicate that some remaining poloxamer actually destabilized the membrane and requires further washout, it is surprising given the relatively short exposure to P188 and the daily exchange of the storage solutions each day post‐isolation. Alternately, the larger number of surviving cells may have created a larger cell pellet causing further dysfunction after storage. While they were statistically significant, all measures were similar to the normal range seen in previous studies (Chung et al., 2015).

In regard to sarcomere length and contractility, prolonged exposure to P188 (Condition 3) again showed the most differences. Again, these results likely reflect an excessive addition of P188 to the system myocytes. Acute exposure to P188 (Condition 2) was very similar to Condition 1, especially upon isolation, but again began to diverge functionally from the Condition 1 control group within 2 days of isolation. Importantly, the major difference was in the diastolic relaxation rate, quantified here as the relengthening velocity. This is likely due to the slowed calcium decline (tau of the Fura Ratio) (Biesiadecki, Davis, Ziolo, & Janssen, 2014), but we cannot exclude any direct or post‐translational sarcomeric alterations that would similarly slow the lengthening velocities.

Overall, the function of the myocytes was within the range of previous work and not clearly different under treatment for 2 days post‐isolation. While pacing threshold of the myocytes was not directly assessed, it was not apparently different. This is important because poloxamers are often used to enhance membrane permeability (Sharma, Stebe, Murphy, & Tung, 1996); we use pluronic acid to enhance Fura2‐AM transit into the cells. The data here were all acquired at 25°C. We also anticipate better function of cells in Condition 2 and possibly worse function in Condition 3 at physiological conditions. Specifically, more damage may occur in Condition 3 due to higher temperatures and pacing rates, altered rates of ion flux and possibly more ion motion through the cell membrane.

Other limitations merit consideration. The otherwise normal function of the cells in all conditions may be due to sampling. Cell function in this study was only measured in rod‐like cells. While the rod‐like cells are likely to show signs of blebbing after storage (Chung et al., 2015), their functional parameters are not consistently altered over several days. Furthermore, this study is also limited in that it did not specifically investigate post‐translational modifications or RNA expression changes, which may impact longer‐term studies or cell‐culture type studies. Post‐translational modifications in downstream signals have been observed (Bajaj et al., 2010). While it is unlikely, it is possible that some of our functional differences may be the result of a post‐translational modification. In general, we did not observe significant dysfunction of myocytes with the addition of P188, which suggests that P188 may be used to improve cell survival of primary isolated intact cardiomyocytes for functional experiments, especially under acute conditions.

## 5 SUMMARY

We continue to seek out improvements in the reproducibility and use of primary isolated intact cardiac myocytes. This study found that the acute addition of Poloxamer 188 (P188) can improve the survival of myocytes upon primary isolation. Isolation methods are similar across species (Abi‐Gerges et al., 2013; Ackers‐Johnson et al., 2016; King et al., 2011; Louch et al., 2011; Makwana et al., 2010). The inclusion of P188 would potentially be beneficial for the primary isolation of intact cardiomyocytes from most species, from mouse to man and possibly avian (Makwana et al., 2010) and reptilian myocytes as well. Additional modifications such as improved storage conditions may continue to reduce the use of animal models and enhance the use of myocytes in cardiovascular research.

## CONFLICT OF INTEREST

None declared.
