# Supplementary material for: Membrane stabilizer Poloxamer 188 improves yield of primary isolated rat cardiomyocytes without impairing function
Source: Physiol Rep. 2020 Feb 28;8(4):e14382. doi: 10.14814/phy2.14382 (PMC7048379; doi:10.14814/phy2.14382)
Supplement: Supplementary file 1 [file PHY2-8-e14382-s001.pdf]

SUPPLEMENTAL DATA

Membrane stabilizer Poloxamer 188 improves yield of primary isolated rat cardiomyocytes without impairing function

Running Title: Improving Cardiomyocyte Survival

Teresa L Czeiszperger, Madison P Wang, and Charles S Chung
Department of Physiology, Wayne State University, Detroit MI USA

Supplemental statistical data for “Membrane stabilizer Poloxamer 188 improves yield of primary isolated rat cardiomyocytes without impairing function”

Statistics include summary statistics (mean, sample size, standard deviation, min, max) and generalized linear model analysis outputs as calculated in SPSS.
Cases excluded indicates no measurements or improperly detected transients.

Page	Parameter
3	Cell Survival (Figure 2B)
22	Baseline Fura Ratio (Figure 3A)
31	Peak Fura Ratio
39	Magnitude of Fura Ratio (Figure 3B)
48	Time to Peak Fura Ratio (Figure 3C)
57	Tau of Fura Ratio Decline (Figure 3D)
66	Baseline (Diastolic) Sarcomere Length (Figure 4A)
75	Peak (Systolic) Sarcomere Length
84	Sarcomere Length Shortening Magnitude (Figure 4B)
92	Time to Peak Sarcomere Length (Figure 4C)
103	Sarcomere Length Shortening Velocity
111	Sarcomere Length Relengthening Velocity
119	Normalized Sarcomere Length Shortening Velocity
130	Normalized Sarcomere Length Relengthening Velocity (Figure 4D)

Cell Survival [Percent rod-shaped cells]

Means

Case Processing Summary

	Cases					
	Included		Excluded		Total	
	N	Percent	N	Percent	N	Percent
Survival * Condition * Number of days post-isolation	194	99.5%	1	0.5%	195	100.0%

Report

Survival

Condition	Number of days post-isolation	Mean	N	Std. Deviation	Minimum	Maximum
1	0	20.122522	18	12.3624229	2.0270	44.1558
	1	10.903680	15	5.2206091	1.8634	18.5263
	2	13.313738	17	5.3560811	4.0000	21.2581
	3	10.931767	9	5.4208208	5.4697	20.9414
	Total	14.414916	59	8.8812770	1.8634	44.1558
2	0	31.228094	18	15.9755216	11.5039	65.6342
	1	14.390312	15	11.0031960	3.9906	48.4496
	2	10.108565	18	4.6339401	3.3864	20.8696
	3	13.176547	15	6.4614213	2.4609	23.2742
	Total	17.538829	66	13.4646271	2.4609	65.6342
3	0	16.205501	18	8.9330694	4.0212	30.8835
	1	8.540034	18	4.8151564	.9816	20.0000
	2	9.341934	18	6.7784508	.7772	26.5574

	3	9.384106	15	5.7124514	2.0070	21.8666
	Total	10.932406	69	7.3501909	.7772	30.8835
Total	0	22.518705	54	14.0615368	2.0270	65.6342
	1	11.106885	48	7.6515029	.9816	48.4496
	2	10.876274	53	5.8163240	.7772	26.5574
	3	11.199890	39	6.0374751	2.0070	23.2742
	Total	14.239066	194	10.5564731	.7772	65.6342

```
* Generalized Linear Models.
GENLIN Survival BY Condition Days (ORDER=ASCENDING)
  /MODEL Condition Days Condition*Days INTERCEPT=YES
 DISTRIBUTION=NORMAL LINK=IDENTITY
  /CRITERIA SCALE=MLE COVB=MODEL PCONVERGE=1E-006(ABSOLUTE) SINGULAR=1E-012 ANALYSISTYPE=3(WALD)
    CILEVEL=95 CITYPE=WALD LIKELIHOOD=FULL
  /EMMEANS TABLES=Condition SCALE=ORIGINAL COMPARE=Condition CONTRAST=PAIRWISE PADJUST=BONFERRONI
  /EMMEANS TABLES=Days SCALE=ORIGINAL COMPARE=Days CONTRAST=PAIRWISE PADJUST=BONFERRONI
  /EMMEANS TABLES=Condition*Days SCALE=ORIGINAL COMPARE=Condition*Days CONTRAST=PAIRWISE
    PADJUST=BONFERRONI
  /MISSING CLASSMISSING=EXCLUDE
  /PRINT CPS DESCRIPTIVES MODELINFO FIT SUMMARY SOLUTION.
```

Generalized Linear Models

Model Information

Dependent Variable	Survival
Probability Distribution	Normal
Link Function	Identity

Case Processing Summary

	N	Percent
Included	194	99.5%
Excluded	1	0.5%
Total	195	100.0%

Categorical Variable Information

			N	Percent
Factor	Condition	1	59	30.4%
		2	66	34.0%
		3	69	35.6%
		Total	194	100.0%
	Number of days post-isolation	0	54	27.8%
		1	48	24.7%
		2	53	27.3%
		3	39	20.1%
		Total	194	100.0%

Continuous Variable Information

		N	Minimum	Maximum	Mean	Std. Deviation
Dependent Variable	Survival	194	.7772	65.6342	14.239066	10.5564731

Goodness of Fit[a]

	Value	df	Value/df
Deviance	13645.686	182	74.976

Scaled Deviance	194.000	182	
Pearson Chi-Square	13645.686	182	74.976
Scaled Pearson Chi-Square	194.000	182	
Log Likelihood[b]	-687.846		
Akaike's Information Criterion (AIC)	1401.692		
Finite Sample Corrected AIC (AICC)	1403.715		
Bayesian Information Criterion (BIC)	1444.174		
Consistent AIC (CAIC)	1457.174		

Dependent Variable: Survival

Model: (Intercept), Condition, Number of days post-isolation, Condition * Number of days post-isolation

a. Information criteria are in smaller-is-better form.

b. The full log likelihood function is displayed and used in computing information criteria.

Omnibus Test[a]

Likelihood Ratio Chi-Square	df	Sig.
88.268	11	.000

Dependent Variable: Survival

Model: (Intercept), Condition, Number of days post-isolation, Condition * Number of days post-isolation

a. Compares the fitted model against the intercept-only model.

Tests of Model Effects

Source	Type III		
	Wald Chi-Square	df	Sig.
(Intercept)	518.971	1	.000
Condition	19.259	2	.000
Number of days post-isolation	71.783	3	.000
Condition * Number of days post-isolation	18.675	6	.005

Dependent Variable: Survival

Model: (Intercept), Condition, Number of days post-isolation, Condition * Number of days post-isolation

Parameter Estimates

Parameter	B	Std. Error	95% Wald Confidence Interval		Hypothesis Test		
			Lower	Upper	Wald Chi-Square	df	Sig.
(Intercept)	9.384	2.1655	5.140	13.628	18.779	1	.000
[Condition=1]	1.548	3.5362	-5.383	8.478	.192	1	.662
[Condition=2]	3.792	3.0624	-2.210	9.795	1.534	1	.216
[Condition=3]	0[a]	.	.	.	.	.	.
[Number of days post-isolation=0]	6.821	2.9321	1.075	12.568	5.413	1	.020
[Number of days post-isolation=1]	-.844	2.9321	-6.591	4.903	.083	1	.773
[Number of days post-isolation=2]	-.042	2.9321	-5.789	5.705	.000	1	.989
[Number of days post-isolation=3]	0[a]	.	.	.	.	.	.
[Condition=1] * [Number of days post-isolation=0]	2.369	4.5078	-6.466	11.204	.276	1	.599

[Condition=1] * [Number of days post-isolation=1]	.816	4.5936	-8.187	9.819	.032	1	.859
[Condition=1] * [Number of days post-isolation=2]	2.424	4.5332	-6.461	11.309	.286	1	.593
[Condition=1] * [Number of days post-isolation=3]	0[a]	.	.	.	.	.	.
[Condition=2] * [Number of days post-isolation=0]	11.230	4.1465	3.103	19.357	7.335	1	.007
[Condition=2] * [Number of days post-isolation=1]	2.058	4.2397	-6.252	10.368	.236	1	.627
[Condition=2] * [Number of days post-isolation=2]	-3.026	4.1465	-11.153	5.101	.532	1	.466
[Condition=2] * [Number of days post-isolation=3]	0[a]	.	.	.	.	.	.
[Condition=3] * [Number of days post-isolation=0]	0[a]	.	.	.	.	.	.
[Condition=3] * [Number of days post-isolation=1]	0[a]	.	.	.	.	.	.
[Condition=3] * [Number of days post-isolation=2]	0[a]	.	.	.	.	.	.
[Condition=3] * [Number of days post-isolation=3]	0[a]	.	.	.	.	.	.
(Scale)	70.339[b]	7.1418	57.646	85.826			

Dependent Variable: Survival

Model: (Intercept), Condition, Number of days post-isolation, Condition * Number of days post-isolation

a. Set to zero because this parameter is redundant.

b. Maximum likelihood estimate.

Estimated Marginal Means 1: Condition

Estimates

Condition	Mean	Std. Error	95% Wald Confidence Interval	
			Lower	Upper
1	13.817927	1.1333000	11.596700	16.039154
2	17.225879	1.0366375	15.194107	19.257652
3	10.867894	1.0128036	8.882835	12.852952

Pairwise Comparisons

(I) Condition	(J) Condition	Mean Difference (I-J)	Std. Error	df	Bonferroni Sig.	95% Wald Confidence Interval for Difference	
						Lower	Upper
1	2	-3.407953	1.5358991	1	.079	-7.084864	.268959
	3	2.950033	1.5199144	1	.157	-.688611	6.588678
2	1	3.407953	1.5358991	1	.079	-.268959	7.084864
	3	6.357986[a]	1.4492717	1	.000	2.888459	9.827513
3	1	-2.950033	1.5199144	1	.157	-6.588678	.688611
	2	-6.357986[a]	1.4492717	1	.000	-9.827513	-2.888459

Pairwise comparisons of estimated marginal means based on the original scale of dependent variable Survival

a. The mean difference is significant at the .05 level.

Overall Test Results

Wald Chi-Square	df	Sig.
19.259	2	.000

The Wald chi-square tests the effect of Condition. This test is based on the linearly independent pairwise comparisons among the estimated marginal means.

Estimated Marginal Means 2: Number of days post-isolation

Estimates

Number of days post-isolation	Mean	Std. Error	95% Wald Confidence Interval	
			Lower	Upper
0	22.518705	1.1413003	20.281798	24.755613
1	11.278008	1.2150070	8.896639	13.659378
2	10.921412	1.1524352	8.662681	13.180144
3	11.164140	1.3821833	8.455110	13.873170

Pairwise Comparisons

(I) Number of days post-isolation	(J) Number of days post-isolation	Mean Difference (I-J)	Std. Error	df	Bonferroni Sig.	95% Wald Confidence Interval for Difference	
						Lower	Upper
0	1	11.240697[a]	1.6669758	1	.000	6.842786	15.638608
	2	11.597293[a]	1.6219351	1	.000	7.318211	15.876375
	3	11.354565[a]	1.7924835	1	.000	6.625533	16.083598
1	0	-11.240697[a]	1.6669758	1	.000	-15.638608	-6.842786
	2	.356596	1.6746190	1	1.000	-4.061480	4.774672
	3	.113868	1.8402915	1	1.000	-4.741294	4.969031

2	0	-11.597293[a]	1.6219351	1	.000	-15.876375	-7.318211
	1	-.356596	1.6746190	1	1.000	-4.774672	4.061480
	3	-.242728	1.7995938	1	1.000	-4.990519	4.505064
3	0	-11.354565[a]	1.7924835	1	.000	-16.083598	-6.625533
	1	-.113868	1.8402915	1	1.000	-4.969031	4.741294
	2	.242728	1.7995938	1	1.000	-4.505064	4.990519

Pairwise comparisons of estimated marginal means based on the original scale of dependent variable Survival

a. The mean difference is significant at the .05 level.

Overall Test Results

Wald Chi-Square	df	Sig.
71.783	3	.000

The Wald chi-square tests the effect of Number of days post-isolation. This test is based on the linearly independent pairwise comparisons among the estimated marginal means.

Estimated Marginal Means 3: Condition* Number of days post-isolation

Estimates

Condition	Number of days post-isolation	Mean	Std. Error	95% Wald Confidence Interval Lower	95% Wald Confidence Interval Upper
1	0	20.122522	1.9767901	16.248085	23.996960
	1	10.903680	2.1654651	6.659446	15.147913
	2	13.313738	2.0341003	9.326974	17.300501

	3	10.931767	2.7956034	5.452485	16.411049
2	0	31.228094	1.9767901	27.353656	35.102531
	1	14.390312	2.1654651	10.146078	18.634545
	2	10.108565	1.9767901	6.234127	13.983002
	3	13.176547	2.1654651	8.932314	17.420781
3	0	16.205501	1.9767901	12.331063	20.079938
	1	8.540034	1.9767901	4.665596	12.414471
	2	9.341934	1.9767901	5.467497	13.216372
	3	9.384106	2.1654651	5.139872	13.628339

Pairwise Comparisons

(I) Condition*Number of days post-isolation	(J) Condition*Number of days post-isolation	Mean Difference (I-J)	Std. Error	df	Bonferroni Sig.	95% Wald Confidence Interval for Difference Lower	Upper
[Condition=1]*[Number of days post-isolation=0]	[Condition=1]*[Number of days post-isolation=1]	9.218842	2.9320536	1	.110	-.655866	19.093551
	[Condition=1]*[Number of days post-isolation=2]	6.808784	2.8364173	1	1.000	-2.743836	16.361404
	[Condition=1]*[Number of days post-isolation=3]	9.190755	3.4239010	1	.480	-2.340420	20.721930
	[Condition=2]*[Number of days post-isolation=0]	-11.105572[a]	2.7956034	1	.005	-20.520737	-1.690406
	[Condition=2]*[Number of days post-isolation=1]	5.732210	2.9320536	1	1.000	-4.142498	15.606919
	[Condition=2]*[Number of days post-isolation=2]	10.013957[a]	2.7956034	1	.023	.598792	19.429122
	[Condition=2]*[Number of days post-isolation=3]	6.945975	2.9320536	1	1.000	-2.928734	16.820683
	[Condition=3]*[Number of days post-isolation=0]	3.917021	2.7956034	1	1.000	-5.498144	13.332187

	[Condition=3]*[Number of days post-isolation=1]	11.582488[a]	2.7956034	1	.002	2.167323	20.997653
	[Condition=3]*[Number of days post-isolation=2]	10.780588[a]	2.7956034	1	.008	1.365423	20.195753
	[Condition=3]*[Number of days post-isolation=3]	10.738416[a]	2.9320536	1	.016	.863708	20.613125
[Condition=1]*[Number of days post-isolation=1]	[Condition=1]*[Number of days post-isolation=0]	-9.218842	2.9320536	1	.110	-19.093551	.655866
	[Condition=1]*[Number of days post-isolation=2]	-2.410058	2.9709936	1	1.000	-12.415910	7.595795
	[Condition=1]*[Number of days post-isolation=3]	-.028087	3.5361897	1	1.000	-11.937434	11.881259
	[Condition=2]*[Number of days post-isolation=0]	-20.324414[a]	2.9320536	1	.000	-30.199122	-10.449705
	[Condition=2]*[Number of days post-isolation=1]	-3.486632	3.0624301	1	1.000	-13.800429	6.827165
	[Condition=2]*[Number of days post-isolation=2]	.795115	2.9320536	1	1.000	-9.079594	10.669823
	[Condition=2]*[Number of days post-isolation=3]	-2.272867	3.0624301	1	1.000	-12.586664	8.040929
	[Condition=3]*[Number of days post-isolation=0]	-5.301821	2.9320536	1	1.000	-15.176529	4.572888
	[Condition=3]*[Number of days post-isolation=1]	2.363646	2.9320536	1	1.000	-7.511062	12.238355
	[Condition=3]*[Number of days post-isolation=2]	1.561746	2.9320536	1	1.000	-8.312963	11.436454
	[Condition=3]*[Number of days post-isolation=3]	1.519574	3.0624301	1	1.000	-8.794222	11.833371
[Condition=1]*[Number of days post-isolation=2]	[Condition=1]*[Number of days post-isolation=0]	-6.808784	2.8364173	1	1.000	-16.361404	2.743836

	[Condition=1]*[Number of days post-isolation=1]	2.410058	2.9709936	1	1.000	-7.595795	12.415910
	[Condition=1]*[Number of days post-isolation=3]	2.381971	3.4573057	1	1.000	-9.261706	14.025648
	[Condition=2]*[Number of days post-isolation=0]	-17.914356[a]	2.8364173	1	.000	-27.466976	-8.361736
	[Condition=2]*[Number of days post-isolation=1]	-1.076574	2.9709936	1	1.000	-11.082427	8.929278
	[Condition=2]*[Number of days post-isolation=2]	3.205173	2.8364173	1	1.000	-6.347447	12.757793
	[Condition=2]*[Number of days post-isolation=3]	.137190	2.9709936	1	1.000	-9.868662	10.143043
	[Condition=3]*[Number of days post-isolation=0]	-2.891763	2.8364173	1	1.000	-12.444383	6.660857
	[Condition=3]*[Number of days post-isolation=1]	4.773704	2.8364173	1	1.000	-4.778916	14.326324
	[Condition=3]*[Number of days post-isolation=2]	3.971804	2.8364173	1	1.000	-5.580817	13.524424
	[Condition=3]*[Number of days post-isolation=3]	3.929632	2.9709936	1	1.000	-6.076220	13.935485
[Condition=1]*[Number of days post-isolation=3]	[Condition=1]*[Number of days post-isolation=0]	-9.190755	3.4239010	1	.480	-20.721930	2.340420
	[Condition=1]*[Number of days post-isolation=1]	.028087	3.5361897	1	1.000	-11.881259	11.937434
	[Condition=1]*[Number of days post-isolation=2]	-2.381971	3.4573057	1	1.000	-14.025648	9.261706
	[Condition=2]*[Number of days post-isolation=0]	-20.296327[a]	3.4239010	1	.000	-31.827502	-8.765151
	[Condition=2]*[Number of days post-isolation=1]	-3.458545	3.5361897	1	1.000	-15.367891	8.450802

	[Condition=2]*[Number of days post-isolation=2]	.823202	3.4239010	1	1.000	-10.707973	12.354377
	[Condition=2]*[Number of days post-isolation=3]	-2.244780	3.5361897	1	1.000	-14.154127	9.664566
	[Condition=3]*[Number of days post-isolation=0]	-5.273734	3.4239010	1	1.000	-16.804909	6.257442
	[Condition=3]*[Number of days post-isolation=1]	2.391733	3.4239010	1	1.000	-9.139442	13.922908
	[Condition=3]*[Number of days post-isolation=2]	1.589833	3.4239010	1	1.000	-9.941342	13.121008
	[Condition=3]*[Number of days post-isolation=3]	1.547661	3.5361897	1	1.000	-10.361685	13.457008
[Condition=2]*[Number of days post-isolation=0]	[Condition=1]*[Number of days post-isolation=0]	11.105572[a]	2.7956034	1	.005	1.690406	20.520737
	[Condition=1]*[Number of days post-isolation=1]	20.324414[a]	2.9320536	1	.000	10.449705	30.199122
	[Condition=1]*[Number of days post-isolation=2]	17.914356[a]	2.8364173	1	.000	8.361736	27.466976
	[Condition=1]*[Number of days post-isolation=3]	20.296327[a]	3.4239010	1	.000	8.765151	31.827502
	[Condition=2]*[Number of days post-isolation=1]	16.837782[a]	2.9320536	1	.000	6.963073	26.712490
	[Condition=2]*[Number of days post-isolation=2]	21.119529[a]	2.7956034	1	.000	11.704363	30.534694
	[Condition=2]*[Number of days post-isolation=3]	18.051546[a]	2.9320536	1	.000	8.176838	27.926255
	[Condition=3]*[Number of days post-isolation=0]	15.022593[a]	2.7956034	1	.000	5.607428	24.437758
	[Condition=3]*[Number of days post-isolation=1]	22.688060[a]	2.7956034	1	.000	13.272895	32.103225

	[Condition=3]*[Number of days post-isolation=2]	21.886159[a]	2.7956034	1	.000	12.470994	31.301325
	[Condition=3]*[Number of days post-isolation=3]	21.843988[a]	2.9320536	1	.000	11.969279	31.718697
[Condition=2]*[Number of days post-isolation=1]	[Condition=1]*[Number of days post-isolation=0]	-5.732210	2.9320536	1	1.000	-15.606919	4.142498
	[Condition=1]*[Number of days post-isolation=1]	3.486632	3.0624301	1	1.000	-6.827165	13.800429
	[Condition=1]*[Number of days post-isolation=2]	1.076574	2.9709936	1	1.000	-8.929278	11.082427
	[Condition=1]*[Number of days post-isolation=3]	3.458545	3.5361897	1	1.000	-8.450802	15.367891
	[Condition=2]*[Number of days post-isolation=0]	-16.837782[a]	2.9320536	1	.000	-26.712490	-6.963073
	[Condition=2]*[Number of days post-isolation=2]	4.281747	2.9320536	1	1.000	-5.592962	14.156455
	[Condition=2]*[Number of days post-isolation=3]	1.213764	3.0624301	1	1.000	-9.100032	11.527561
	[Condition=3]*[Number of days post-isolation=0]	-1.815189	2.9320536	1	1.000	-11.689897	8.059520
	[Condition=3]*[Number of days post-isolation=1]	5.850278	2.9320536	1	1.000	-4.024430	15.724987
	[Condition=3]*[Number of days post-isolation=2]	5.048378	2.9320536	1	1.000	-4.826331	14.923086
	[Condition=3]*[Number of days post-isolation=3]	5.006206	3.0624301	1	1.000	-5.307590	15.320003
[Condition=2]*[Number of days post-isolation=2]	[Condition=1]*[Number of days post-isolation=0]	-10.013957[a]	2.7956034	1	.023	-19.429122	-.598792
	[Condition=1]*[Number of days post-isolation=1]	-.795115	2.9320536	1	1.000	-10.669823	9.079594

	[Condition=1]*[Number of days post-isolation=2]	-3.205173	2.8364173	1	1.000	-12.757793	6.347447
	[Condition=1]*[Number of days post-isolation=3]	-.823202	3.4239010	1	1.000	-12.354377	10.707973
	[Condition=2]*[Number of days post-isolation=0]	-21.119529[a]	2.7956034	1	.000	-30.534694	-11.704363
	[Condition=2]*[Number of days post-isolation=1]	-4.281747	2.9320536	1	1.000	-14.156455	5.592962
	[Condition=2]*[Number of days post-isolation=3]	-3.067982	2.9320536	1	1.000	-12.942691	6.806726
	[Condition=3]*[Number of days post-isolation=0]	-6.096936	2.7956034	1	1.000	-15.512101	3.318229
	[Condition=3]*[Number of days post-isolation=1]	1.568531	2.7956034	1	1.000	-7.846634	10.983696
	[Condition=3]*[Number of days post-isolation=2]	.766631	2.7956034	1	1.000	-8.648534	10.181796
	[Condition=3]*[Number of days post-isolation=3]	.724459	2.9320536	1	1.000	-9.150249	10.599168
[Condition=2]*[Number of days post-isolation=3]	[Condition=1]*[Number of days post-isolation=0]	-6.945975	2.9320536	1	1.000	-16.820683	2.928734
	[Condition=1]*[Number of days post-isolation=1]	2.272867	3.0624301	1	1.000	-8.040929	12.586664
	[Condition=1]*[Number of days post-isolation=2]	-.137190	2.9709936	1	1.000	-10.143043	9.868662
	[Condition=1]*[Number of days post-isolation=3]	2.244780	3.5361897	1	1.000	-9.664566	14.154127
	[Condition=2]*[Number of days post-isolation=0]	-18.051546[a]	2.9320536	1	.000	-27.926255	-8.176838
	[Condition=2]*[Number of days post-isolation=1]	-1.213764	3.0624301	1	1.000	-11.527561	9.100032

	[Condition=2]*[Number of days post-isolation=2]	3.067982	2.9320536	1	1.000	-6.806726	12.942691
	[Condition=3]*[Number of days post-isolation=0]	-3.028953	2.9320536	1	1.000	-12.903662	6.845755
	[Condition=3]*[Number of days post-isolation=1]	4.636514	2.9320536	1	1.000	-5.238195	14.511222
	[Condition=3]*[Number of days post-isolation=2]	3.834613	2.9320536	1	1.000	-6.040095	13.709322
	[Condition=3]*[Number of days post-isolation=3]	3.792442	3.0624301	1	1.000	-6.521355	14.106238
[Condition=3]*[Number of days post-isolation=0]	[Condition=1]*[Number of days post-isolation=0]	-3.917021	2.7956034	1	1.000	-13.332187	5.498144
	[Condition=1]*[Number of days post-isolation=1]	5.301821	2.9320536	1	1.000	-4.572888	15.176529
	[Condition=1]*[Number of days post-isolation=2]	2.891763	2.8364173	1	1.000	-6.660857	12.444383
	[Condition=1]*[Number of days post-isolation=3]	5.273734	3.4239010	1	1.000	-6.257442	16.804909
	[Condition=2]*[Number of days post-isolation=0]	-15.022593[a]	2.7956034	1	.000	-24.437758	-5.607428
	[Condition=2]*[Number of days post-isolation=1]	1.815189	2.9320536	1	1.000	-8.059520	11.689897
	[Condition=2]*[Number of days post-isolation=2]	6.096936	2.7956034	1	1.000	-3.318229	15.512101
	[Condition=2]*[Number of days post-isolation=3]	3.028953	2.9320536	1	1.000	-6.845755	12.903662
	[Condition=3]*[Number of days post-isolation=1]	7.665467	2.7956034	1	.403	-1.749698	17.080632
	[Condition=3]*[Number of days post-isolation=2]	6.863567	2.7956034	1	.930	-2.551599	16.278732

	[Condition=3]*[Number of days post-isolation=3]	6.821395	2.9320536	1	1.000	-3.053313	16.696104
[Condition=3]*[Number of days post-isolation=1]	[Condition=1]*[Number of days post-isolation=0]	-11.582488[a]	2.7956034	1	.002	-20.997653	-2.167323
	[Condition=1]*[Number of days post-isolation=1]	-2.363646	2.9320536	1	1.000	-12.238355	7.511062
	[Condition=1]*[Number of days post-isolation=2]	-4.773704	2.8364173	1	1.000	-14.326324	4.778916
	[Condition=1]*[Number of days post-isolation=3]	-2.391733	3.4239010	1	1.000	-13.922908	9.139442
	[Condition=2]*[Number of days post-isolation=0]	-22.688060[a]	2.7956034	1	.000	-32.103225	-13.272895
	[Condition=2]*[Number of days post-isolation=1]	-5.850278	2.9320536	1	1.000	-15.724987	4.024430
	[Condition=2]*[Number of days post-isolation=2]	-1.568531	2.7956034	1	1.000	-10.983696	7.846634
	[Condition=2]*[Number of days post-isolation=3]	-4.636514	2.9320536	1	1.000	-14.511222	5.238195
	[Condition=3]*[Number of days post-isolation=0]	-7.665467	2.7956034	1	.403	-17.080632	1.749698
	[Condition=3]*[Number of days post-isolation=2]	-.801900	2.7956034	1	1.000	-10.217066	8.613265
	[Condition=3]*[Number of days post-isolation=3]	-.844072	2.9320536	1	1.000	-10.718780	9.030637
[Condition=3]*[Number of days post-isolation=2]	[Condition=1]*[Number of days post-isolation=0]	-10.780588[a]	2.7956034	1	.008	-20.195753	-1.365423
	[Condition=1]*[Number of days post-isolation=1]	-1.561746	2.9320536	1	1.000	-11.436454	8.312963
	[Condition=1]*[Number of days post-isolation=2]	-3.971804	2.8364173	1	1.000	-13.524424	5.580817

	[Condition=1]*[Number of days post-isolation=3]	-1.589833	3.4239010	1	1.000	-13.121008	9.941342
	[Condition=2]*[Number of days post-isolation=0]	-21.886159[a]	2.7956034	1	.000	-31.301325	-12.470994
	[Condition=2]*[Number of days post-isolation=1]	-5.048378	2.9320536	1	1.000	-14.923086	4.826331
	[Condition=2]*[Number of days post-isolation=2]	-.766631	2.7956034	1	1.000	-10.181796	8.648534
	[Condition=2]*[Number of days post-isolation=3]	-3.834613	2.9320536	1	1.000	-13.709322	6.040095
	[Condition=3]*[Number of days post-isolation=0]	-6.863567	2.7956034	1	.930	-16.278732	2.551599
	[Condition=3]*[Number of days post-isolation=1]	.801900	2.7956034	1	1.000	-8.613265	10.217066
	[Condition=3]*[Number of days post-isolation=3]	-.042171	2.9320536	1	1.000	-9.916880	9.832537
[Condition=3]*[Number of days post-isolation=3]	[Condition=1]*[Number of days post-isolation=0]	-10.738416[a]	2.9320536	1	.016	-20.613125	-.863708
	[Condition=1]*[Number of days post-isolation=1]	-1.519574	3.0624301	1	1.000	-11.833371	8.794222
	[Condition=1]*[Number of days post-isolation=2]	-3.929632	2.9709936	1	1.000	-13.935485	6.076220
	[Condition=1]*[Number of days post-isolation=3]	-1.547661	3.5361897	1	1.000	-13.457008	10.361685
	[Condition=2]*[Number of days post-isolation=0]	-21.843988[a]	2.9320536	1	.000	-31.718697	-11.969279
	[Condition=2]*[Number of days post-isolation=1]	-5.006206	3.0624301	1	1.000	-15.320003	5.307590
	[Condition=2]*[Number of days post-isolation=2]	-.724459	2.9320536	1	1.000	-10.599168	9.150249

	[Condition=2]*[Number of days post-isolation=3]	-3.792442	3.0624301	1	1.000	-14.106238	6.521355
	[Condition=3]*[Number of days post-isolation=0]	-6.821395	2.9320536	1	1.000	-16.696104	3.053313
	[Condition=3]*[Number of days post-isolation=1]	.844072	2.9320536	1	1.000	-9.030637	10.718780
	[Condition=3]*[Number of days post-isolation=2]	.042171	2.9320536	1	1.000	-9.832537	9.916880

Pairwise comparisons of estimated marginal means based on the original scale of dependent variable Survival

a. The mean difference is significant at the .05 level.

Overall Test Results

Wald Chi-Square	df	Sig.
111.775	11	.000

The Wald chi-square tests the effect of Condition*Number of days post-isolation. This test is based on the linearly independent pairwise comparisons among the estimated marginal means.

Baseline Fura Ratio

```
MEANS TABLES=Fura_Baseline BY Condition BY Days
  /CELLS=MEAN COUNT STDDEV MIN MAX.
```

Means

Case Processing Summary

	Cases					
	Included		Excluded		Total	
	N	Percent	N	Percent	N	Percent
Baseline Fura Ratio * Condition * Days	662	98.5%	10	1.5%	672	100.0%

Report

Baseline Fura Ratio

Condition	Days	Mean	N	Std. Deviation	Minimum	Maximum
1	0	.9687416	140	.10952299	.78515	1.53230
	1	.8864527	51	.03975514	.83191	1.05640
	2	.9518146	40	.10611260	.83160	1.39916
	Total	.9476428	231	.10298751	.78515	1.53230
2	0	.9503423	136	.06403738	.84588	1.12747
	1	.8669119	38	.03164331	.80156	.98195
	2	.8922957	53	.04678102	.83674	1.12708
	Total	.9228233	227	.06570349	.80156	1.12747

3	0	.9207545	123	.07938579	.82914	1.44806
	1	.8786995	48	.05568815	.82690	1.18959
	2	.8833864	33	.03898114	.83141	1.00317
	Total	.9048144	204	.07165796	.82690	1.44806
Total	0	.9476772	399	.08885151	.78515	1.53230
	1	.8783162	137	.04456919	.80156	1.18959
	2	.9088572	126	.07543582	.83141	1.39916
	Total	.9259343	662	.08395967	.78515	1.53230

```
* Generalized Linear Models.
GENLIN Fura_Baseline BY Condition Days (ORDER=ASCENDING)
  /MODEL Condition Days Condition*Days INTERCEPT=YES
 DISTRIBUTION=NORMAL LINK=IDENTITY
  /CRITERIA SCALE=MLE COVB=MODEL PCONVERGE=1E-006(ABSOLUTE) SINGULAR=1E-012 ANALYSISTYPE=3(WALD)
    CILEVEL=95 CITYPE=WALD LIKELIHOOD=FULL
  /EMMEANS TABLES=Condition*Days SCALE=ORIGINAL COMPARE=Condition*Days CONTRAST=PAIRWISE
    PADJUST=BONFERRONI
  /MISSING CLASSMISSING=EXCLUDE
  /PRINT CPS DESCRIPTIVES MODELINFO FIT SUMMARY SOLUTION.
```

Generalized Linear Models

Model Information

Dependent Variable	Baseline Fura Ratio
Probability Distribution	Normal
Link Function	Identity

Case Processing Summary

	N	Percent
Included	662	98.5%
Excluded	10	1.5%
Total	672	100.0%

Categorical Variable Information

			N	Percent
Factor	Condition	1	231	34.9%
		2	227	34.3%
		3	204	30.8%
		Total	662	100.0%
	Days	0	399	60.3%
		1	137	20.7%
		2	126	19.0%
		Total	662	100.0%

Continuous Variable Information

		N	Minimum	Maximum	Mean	Std. Deviation
Dependent Variable	Baseline Fura Ratio	662	.78515	1.53230	.9259343	.08395967

Goodness of Fit[a]

	Value	df	Value/df
Deviance	3.853	653	.006
Scaled Deviance	662.000	653	
Pearson Chi-Square	3.853	653	.006
Scaled Pearson Chi-Square	662.000	653	

Log Likelihood[b]	764.109		
Akaike's Information Criterion (AIC)	-1508.218		
Finite Sample Corrected AIC (AICC)	-1507.880		
Bayesian Information Criterion (BIC)	-1463.265		
Consistent AIC (CAIC)	-1453.265		

Dependent Variable: Baseline Fura Ratio

Model: (Intercept), Condition, Days, Condition * Days

a. Information criteria are in smaller-is-better form.

b. The full log likelihood function is displayed and used in computing information criteria.

Omnibus Test[a]

Likelihood Ratio Chi-Square	df	Sig.
125.789	8	.000

Dependent Variable: Baseline Fura Ratio

Model: (Intercept), Condition, Days, Condition * Days

a. Compares the fitted model against the intercept-only model.

Tests of Model Effects

Source	Type III Wald Chi-Square	df	Sig.
(Intercept)	70622.077	1	.000
Condition	27.217	2	.000

Days	89.713	2	.000
Condition * Days	12.161	4	.016

Dependent Variable: Baseline Fura Ratio

Model: (Intercept), Condition, Days, Condition * Days

Parameter Estimates

Parameter	B	Std. Error	95% Wald Confidence Interval		Hypothesis Test		
			Lower	Upper	Wald Chi-Square	df	Sig.
(Intercept)	.883	.0133	.857	.909	4424.380	1	.000
[Condition=1]	.068	.0179	.033	.104	14.547	1	.000
[Condition=2]	.009	.0169	-.024	.042	.277	1	.598
[Condition=3]	0[a]	.	.	.	.	.	.
[Days=0]	.037	.0150	.008	.067	6.242	1	.012
[Days=1]	-.005	.0173	-.039	.029	.074	1	.786
[Days=2]	0[a]	.	.	.	.	.	.
[Condition=1] * [Days=0]	-.020	.0203	-.060	.019	1.017	1	.313
[Condition=1] * [Days=1]	-.061	.0236	-.107	-.014	6.606	1	.010
[Condition=1] * [Days=2]	0[a]	.	.	.	.	.	.
[Condition=2] * [Days=0]	.021	.0194	-.017	.059	1.136	1	.286
[Condition=2] * [Days=1]	-.021	.0237	-.067	.026	.764	1	.382
[Condition=2] * [Days=2]	0[a]	.	.	.	.	.	.
[Condition=3] * [Days=0]	0[a]	.	.	.	.	.	.
[Condition=3] * [Days=1]	0[a]	.	.	.	.	.	.
[Condition=3] * [Days=2]	0[a]	.	.	.	.	.	.
(Scale)	.006[b]	.0003	.005	.006			

Dependent Variable: Baseline Fura Ratio

Model: (Intercept), Condition, Days, Condition * Days

a. Set to zero because this parameter is redundant.

b. Maximum likelihood estimate.

Estimated Marginal Means: Condition* Days

Estimates

Condition	Days	Mean	Std. Error	95% Wald Confidence Interval Lower	95% Wald Confidence Interval Upper
1	0	.9687416	.00644789	.9561040	.9813792
	1	.8864527	.01068308	.8655142	.9073911
	2	.9518146	.01206289	.9281718	.9754575
2	0	.9503423	.00654202	.9375202	.9631645
	1	.8669119	.01237627	.8426549	.8911690
	2	.8922957	.01047957	.8717561	.9128353
3	0	.9207545	.00687906	.9072718	.9342372
	1	.8786995	.01101186	.8571166	.9002823
	2	.8833864	.01328081	.8573565	.9094163

Pairwise Comparisons

(I) Condition*Days	(J) Condition*Days	Mean Difference (I-J)	Std. Error	df	Bonferroni Sig.	95% Wald Confidence Interval for Difference Lower	95% Wald Confidence Interval for Difference Upper
[Condition=1]*[Days=0]	[Condition=1]*[Days=1]	.0822890[a]	.01247812	1	.000	.0423970	.1221809
	[Condition=1]*[Days=2]	.0169270	.01367803	1	1.000	-.0268010	.0606550
	[Condition=2]*[Days=0]	.0183993	.00918549	1	1.000	-.0109663	.0477649
	[Condition=2]*[Days=1]	.1018297[a]	.01395519	1	.000	.0572156	.1464437
	[Condition=2]*[Days=2]	.0764459[a]	.01230434	1	.000	.0371096	.1157823

	[Condition=3]*[Days=0]	.0479871[a]	.00942850	1	.000	.0178446	.0781296
	[Condition=3]*[Days=1]	.0900422[a]	.01276074	1	.000	.0492467	.1308376
	[Condition=3]*[Days=2]	.0853552[a]	.01476330	1	.000	.0381577	.1325528
[Condition=1]*[Days=1]	[Condition=1]*[Days=0]	-.0822890[a]	.01247812	1	.000	-.1221809	-.0423970
	[Condition=1]*[Days=2]	-.0653620[a]	.01611340	1	.002	-.1168757	-.0138483
	[Condition=2]*[Days=0]	-.0638897[a]	.01252702	1	.000	-.1039379	-.0238414
	[Condition=2]*[Days=1]	.0195407	.01634932	1	1.000	-.0327272	.0718087
	[Condition=2]*[Days=2]	-.0058430	.01496494	1	1.000	-.0536852	.0419992
	[Condition=3]*[Days=0]	-.0343019	.01270628	1	.250	-.0749232	.0063195
	[Condition=3]*[Days=1]	.0077532	.01534240	1	1.000	-.0412957	.0568021
	[Condition=3]*[Days=2]	.0030663	.01704430	1	1.000	-.0514235	.0575560
[Condition=1]*[Days=2]	[Condition=1]*[Days=0]	-.0169270	.01367803	1	1.000	-.0606550	.0268010
	[Condition=1]*[Days=1]	.0653620[a]	.01611340	1	.002	.0138483	.1168757
	[Condition=2]*[Days=0]	.0014723	.01372266	1	1.000	-.0423984	.0453430
	[Condition=2]*[Days=1]	.0849027[a]	.01728252	1	.000	.0296514	.1401540
	[Condition=2]*[Days=2]	.0595190[a]	.01597920	1	.007	.0084343	.1106037
	[Condition=3]*[Days=0]	.0310601	.01388650	1	.911	-.0133343	.0754546
	[Condition=3]*[Days=1]	.0731152[a]	.01633323	1	.000	.0208986	.1253317
	[Condition=3]*[Days=2]	.0684283[a]	.01794138	1	.005	.0110705	.1257860
[Condition=2]*[Days=0]	[Condition=1]*[Days=0]	-.0183993	.00918549	1	1.000	-.0477649	.0109663
	[Condition=1]*[Days=1]	.0638897[a]	.01252702	1	.000	.0238414	.1039379
	[Condition=1]*[Days=2]	-.0014723	.01372266	1	1.000	-.0453430	.0423984
	[Condition=2]*[Days=1]	.0834304[a]	.01399893	1	.000	.0386765	.1281843
	[Condition=2]*[Days=2]	.0580467[a]	.01235393	1	.000	.0185518	.0975415
	[Condition=3]*[Days=0]	.0295878	.00949313	1	.066	-.0007612	.0599369
	[Condition=3]*[Days=1]	.0716429[a]	.01280856	1	.000	.0306945	.1125912
	[Condition=3]*[Days=2]	.0669560[a]	.01480466	1	.000	.0196262	.1142857
[Condition=2]*[Days=1]	[Condition=1]*[Days=0]	-.1018297[a]	.01395519	1	.000	-.1464437	-.0572156
	[Condition=1]*[Days=1]	-.0195407	.01634932	1	1.000	-.0718087	.0327272
	[Condition=1]*[Days=2]	-.0849027[a]	.01728252	1	.000	-.1401540	-.0296514

	[Condition=2]*[Days=0]	-.0834304[a]	.01399893	1	.000	-.1281843	-.0386765
	[Condition=2]*[Days=2]	-.0253837	.01621707	1	1.000	-.0772289	.0264614
	[Condition=3]*[Days=0]	-.0538426[a]	.01415957	1	.005	-.0991100	-.0085751
	[Condition=3]*[Days=1]	-.0117875	.01656602	1	1.000	-.0647483	.0411732
	[Condition=3]*[Days=2]	-.0164744	.01815356	1	1.000	-.0745105	.0415616
[Condition=2]*[Days=2]	[Condition=1]*[Days=0]	-.0764459[a]	.01230434	1	.000	-.1157823	-.0371096
	[Condition=1]*[Days=1]	.0058430	.01496494	1	1.000	-.0419992	.0536852
	[Condition=1]*[Days=2]	-.0595190[a]	.01597920	1	.007	-.1106037	-.0084343
	[Condition=2]*[Days=0]	-.0580467[a]	.01235393	1	.000	-.0975415	-.0185518
	[Condition=2]*[Days=1]	.0253837	.01621707	1	1.000	-.0264614	.0772289
	[Condition=3]*[Days=0]	-.0284588	.01253566	1	.835	-.0685347	.0116170
	[Condition=3]*[Days=1]	.0135962	.01520140	1	1.000	-.0350019	.0621943
	[Condition=3]*[Days=2]	.0089093	.01691748	1	1.000	-.0451751	.0629937
[Condition=3]*[Days=0]	[Condition=1]*[Days=0]	-.0479871[a]	.00942850	1	.000	-.0781296	-.0178446
	[Condition=1]*[Days=1]	.0343019	.01270628	1	.250	-.0063195	.0749232
	[Condition=1]*[Days=2]	-.0310601	.01388650	1	.911	-.0754546	.0133343
	[Condition=2]*[Days=0]	-.0295878	.00949313	1	.066	-.0599369	.0007612
	[Condition=2]*[Days=1]	.0538426[a]	.01415957	1	.005	.0085751	.0991100
	[Condition=2]*[Days=2]	.0284588	.01253566	1	.835	-.0116170	.0685347
	[Condition=3]*[Days=1]	.0420551[a]	.01298394	1	.043	.0005461	.0835641
	[Condition=3]*[Days=2]	.0373681	.01495665	1	.449	-.0104475	.0851838
[Condition=3]*[Days=1]	[Condition=1]*[Days=0]	-.0900422[a]	.01276074	1	.000	-.1308376	-.0492467
	[Condition=1]*[Days=1]	-.0077532	.01534240	1	1.000	-.0568021	.0412957
	[Condition=1]*[Days=2]	-.0731152[a]	.01633323	1	.000	-.1253317	-.0208986
	[Condition=2]*[Days=0]	-.0716429[a]	.01280856	1	.000	-.1125912	-.0306945
	[Condition=2]*[Days=1]	.0117875	.01656602	1	1.000	-.0411732	.0647483
	[Condition=2]*[Days=2]	-.0135962	.01520140	1	1.000	-.0621943	.0350019
	[Condition=3]*[Days=0]	-.0420551[a]	.01298394	1	.043	-.0835641	-.0005461
	[Condition=3]*[Days=2]	-.0046869	.01725228	1	1.000	-.0598416	.0504678
[Condition=3]*[Days=2]	[Condition=1]*[Days=0]	-.0853552[a]	.01476330	1	.000	-.1325528	-.0381577

	[Condition=1]*[Days=1]	-.0030663	.01704430	1	1.000	-.0575560	.0514235
	[Condition=1]*[Days=2]	-.0684283[a]	.01794138	1	.005	-.1257860	-.0110705
	[Condition=2]*[Days=0]	-.0669560[a]	.01480466	1	.000	-.1142857	-.0196262
	[Condition=2]*[Days=1]	.0164744	.01815356	1	1.000	-.0415616	.0745105
	[Condition=2]*[Days=2]	-.0089093	.01691748	1	1.000	-.0629937	.0451751
	[Condition=3]*[Days=0]	-.0373681	.01495665	1	.449	-.0851838	.0104475
	[Condition=3]*[Days=1]	.0046869	.01725228	1	1.000	-.0504678	.0598416

Pairwise comparisons of estimated marginal means based on the original scale of dependent variable Baseline Fura Ratio

a. The mean difference is significant at the .05 level.

Overall Test Results

Wald Chi-Square	df	Sig.
138.534	8	.000

The Wald chi-square tests the effect of Condition*Days. This test is based on the linearly independent pairwise comparisons among the estimated marginal means.

Peak Fura Ratio

```
MEANS TABLES=Fura_Peak BY Condition BY Days
  /CELLS=MEAN COUNT STDDEV MIN MAX.
```

Means

Case Processing Summary

	Cases					
	Included		Excluded		Total	
	N	Percent	N	Percent	N	Percent
Peak Fura Ratio * Condition * Days	662	98.5%	10	1.5%	672	100.0%

Report

Peak Fura Ratio

Condition	Days	Mean	N	Std. Deviation	Minimum	Maximum
1	0	1.3058479	140	.13532698	.86000	1.82556
	1	1.2041781	51	.13951497	.94089	1.46958
	2	1.2026424	40	.15292912	.92209	1.65105
	Total	1.2655303	231	.14756905	.86000	1.82556
2	0	1.2669931	136	.09585433	1.03321	1.54816
	1	1.1486058	38	.13210385	.90933	1.46848
	2	1.1649161	53	.15055371	.92182	1.56033
	Total	1.2233420	227	.12824634	.90933	1.56033
3	0	1.2601392	123	.14760440	.96595	1.90700

	1	1.0995157	48	.16950157	.88550	1.73448
	2	1.0849023	33	.15593263	.87941	1.59725
	Total	1.1939983	204	.17399653	.87941	1.90700
Total	0	1.2785135	399	.12885415	.86000	1.90700
	1	1.1520939	137	.15436727	.88550	1.73448
	2	1.1559367	126	.15816152	.87941	1.65105
	Total	1.2290208	662	.15281973	.86000	1.90700

```
* Generalized Linear Models.
GENLIN Fura_Peak BY Condition Days (ORDER=ASCENDING)
  /MODEL Condition Days Condition*Days INTERCEPT=YES
 DISTRIBUTION=NORMAL LINK=IDENTITY
  /CRITERIA SCALE=MLE COVB=MODEL PCONVERGE=1E-006(ABSOLUTE) SINGULAR=1E-012 ANALYSISTYPE=3(WALD)
    CILEVEL=95 CITYPE=WALD LIKELIHOOD=FULL
  /EMMEANS TABLES=Condition SCALE=ORIGINAL COMPARE=Condition CONTRAST=PAIRWISE PADJUST=BONFERRONI
  /EMMEANS TABLES=Days SCALE=ORIGINAL COMPARE=Days CONTRAST=PAIRWISE PADJUST=BONFERRONI
  /MISSING CLASSMISSING=EXCLUDE
  /PRINT CPS DESCRIPTIVES MODELINFO FIT SUMMARY SOLUTION.
```

Generalized Linear Models

Model Information

Dependent Variable	Peak Fura Ratio
Probability Distribution	Normal
Link Function	Identity

Case Processing Summary

	N	Percent
Included	662	98.5%
Excluded	10	1.5%
Total	672	100.0%

Categorical Variable Information

			N	Percent
Factor	Condition	1	231	34.9%
		2	227	34.3%
		3	204	30.8%
		Total	662	100.0%
	Days	0	399	60.3%
		1	137	20.7%
		2	126	19.0%
		Total	662	100.0%

Continuous Variable Information

		N	Minimum	Maximum	Mean	Std. Deviation
Dependent Variable	Peak Fura Ratio	662	.86000	1.90700	1.2290208	.15281973

Goodness of Fit[a]

	Value	df	Value/df
Deviance	12.282	653	.019
Scaled Deviance	662.000	653	
Pearson Chi-Square	12.282	653	.019
Scaled Pearson Chi-Square	662.000	653	
Log Likelihood[b]	380.401		

Akaike's Information Criterion (AIC)	-740.802		
Finite Sample Corrected AIC (AICC)	-740.464		
Bayesian Information Criterion (BIC)	-695.849		
Consistent AIC (CAIC)	-685.849		

Dependent Variable: Peak Fura Ratio

Model: (Intercept), Condition, Days, Condition * Days

a. Information criteria are in smaller-is-better form.

b. The full log likelihood function is displayed and used in computing information criteria.

Omnibus Test[a]

Likelihood Ratio Chi-Square	df	Sig.
151.346	8	.000

Dependent Variable: Peak Fura Ratio

Model: (Intercept), Condition, Days, Condition * Days

a. Compares the fitted model against the intercept-only model.

Tests of Model Effects

Source	Type III		
	Wald Chi-Square	df	Sig.
(Intercept)	37997.173	1	.000
Condition	34.935	2	.000
Days	135.177	2	.000

Condition * Days	7.338	4	.119

Dependent Variable: Peak Fura Ratio

Model: (Intercept), Condition, Days, Condition * Days

Parameter Estimates

Parameter	B	Std. Error	95% Wald Confidence Interval		Hypothesis Test		
			Lower	Upper	Wald Chi-Square	df	Sig.
(Intercept)	1.085	.0237	1.038	1.131	2093.540	1	.000
[Condition=1]	.118	.0320	.055	.181	13.511	1	.000
[Condition=2]	.080	.0302	.021	.139	7.018	1	.008
[Condition=3]	0[a]	.	.	.	.	.	.
[Days=0]	.175	.0267	.123	.228	43.066	1	.000
[Days=1]	.015	.0308	-.046	.075	.225	1	.635
[Days=2]	0[a]	.	.	.	.	.	.
[Condition=1] * [Days=0]	-.072	.0362	-.143	-.001	3.963	1	.047
[Condition=1] * [Days=1]	-.013	.0421	-.096	.070	.096	1	.756
[Condition=1] * [Days=2]	0[a]	.	.	.	.	.	.
[Condition=2] * [Days=0]	-.073	.0346	-.141	-.005	4.462	1	.035
[Condition=2] * [Days=1]	-.031	.0423	-.114	.052	.535	1	.464
[Condition=2] * [Days=2]	0[a]	.	.	.	.	.	.
[Condition=3] * [Days=0]	0[a]	.	.	.	.	.	.
[Condition=3] * [Days=1]	0[a]	.	.	.	.	.	.
[Condition=3] * [Days=2]	0[a]	.	.	.	.	.	.
(Scale)	.019[b]	.0010	.017	.021			

Dependent Variable: Peak Fura Ratio

Model: (Intercept), Condition, Days, Condition * Days

a. Set to zero because this parameter is redundant.

b. Maximum likelihood estimate.

Estimated Marginal Means 1: Condition

Estimates

Condition	Mean	Std. Error	95% Wald Confidence Interval Lower	95% Wald Confidence Interval Upper
1	1.2375562	.01032866	1.2173124	1.2578000
2	1.1935050	.01040679	1.1731081	1.2139019
3	1.1481857	.01105325	1.1265218	1.1698497

Pairwise Comparisons

(I) Condition	(J) Condition	Mean Difference (I-J)	Std. Error	df	Bonferroni Sig.	95% Wald Confidence Interval for Difference Lower	95% Wald Confidence Interval for Difference Upper
1	2	.0440512[a]	.01466228	1	.008	.0089500	.0791524
	3	.0893704[a]	.01512797	1	.000	.0531544	.1255865
2	1	-.0440512[a]	.01466228	1	.008	-.0791524	-.0089500
	3	.0453193[a]	.01518143	1	.009	.0089752	.0816633
3	1	-.0893704[a]	.01512797	1	.000	-.1255865	-.0531544
	2	-.0453193[a]	.01518143	1	.009	-.0816633	-.0089752

Pairwise comparisons of estimated marginal means based on the original scale of dependent variable Peak Fura Ratio

a. The mean difference is significant at the .05 level.

Overall Test Results

Wald Chi-Square	df	Sig.

34.935	2	.000

The Wald chi-square tests the effect of Condition. This test is based on the linearly independent pairwise comparisons among the estimated marginal means.

Estimated Marginal Means 2: Days

Estimates

Days	Mean	Std. Error	95% Wald Confidence Interval	
			Lower	Upper
0	1.2776601	.00682949	1.2642745	1.2910456
1	1.1507666	.01173097	1.1277743	1.1737588
2	1.1508203	.01236524	1.1265848	1.1750557

Pairwise Comparisons

(I) Days	(J) Days	Mean Difference (I-J)	Std. Error	df	Bonferroni Sig.	95% Wald Confidence Interval for Difference	
						Lower	Upper
0	1	.1268935[a]	.01357415	1	.000	.0943973	.1593898
	2	.1268398[a]	.01412590	1	.000	.0930227	.1606569
1	0	-.1268935[a]	.01357415	1	.000	-.1593898	-.0943973
	2	-.0000537	.01704450	1	1.000	-.0408579	.0407505
2	0	-.1268398[a]	.01412590	1	.000	-.1606569	-.0930227
	1	.0000537	.01704450	1	1.000	-.0407505	.0408579

Pairwise comparisons of estimated marginal means based on the original scale of dependent variable Peak Fura Ratio

a. The mean difference is significant at the .05 level.

Overall Test Results

Wald Chi-Square	df	Sig.
135.177	2	.000

The Wald chi-square tests the effect of Days. This test is based on the linearly independent pairwise comparisons among the estimated marginal means.

Magnitude of Fura Ratio

```
MEANS TABLES=Fura_Mag BY Condition BY Days
  /CELLS=MEAN COUNT STDDEV MIN MAX.
```

Means

Case Processing Summary

	Cases					
	Included		Excluded		Total	
	N	Percent	N	Percent	N	Percent
Fura Ratio Magnitude * Condition * Days	662	98.5%	10	1.5%	672	100.0%

Report

Fura Ratio Magnitude

Condition	Days	Mean	N	Std. Deviation	Minimum	Maximum
1	0	.3371120	140	.11340043	.07480	.81840
	1	.3177091	51	.12440887	.09600	.58942
	2	.2514258	40	.08654693	.07273	.40167
	Total	.3179908	231	.11575691	.07273	.81840
2	0	.3166400	136	.09940403	.10528	.57222
	1	.2817047	38	.12438164	.04915	.53880
	2	.2729942	53	.12199807	.05753	.53884
	Total	.3006014	227	.11069506	.04915	.57222
3	0	.3393861	123	.12281275	.06800	.72961

	1	.2208072	48	.13637149	.04741	.75828
	2	.2015559	33	.14479393	.02924	.70838
	Total	.2891891	204	.14337392	.02924	.75828
Total	0	.3308351	399	.11212302	.06800	.81840
	1	.2737714	137	.13437938	.04741	.75828
	2	.2474371	126	.12138134	.02924	.70838
	Total	.3031525	662	.12371082	.02924	.81840

```
* Generalized Linear Models.
GENLIN Fura_Mag BY Condition Days (ORDER=ASCENDING)
  /MODEL Condition Days Condition*Days INTERCEPT=YES
 DISTRIBUTION=NORMAL LINK=IDENTITY
  /CRITERIA SCALE=MLE COVB=MODEL PCONVERGE=1E-006(ABSOLUTE) SINGULAR=1E-012 ANALYSISTYPE=3(WALD)
    CILEVEL=95 CITYPE=WALD LIKELIHOOD=FULL
  /EMMEANS TABLES=Condition*Days SCALE=ORIGINAL COMPARE=Condition*Days CONTRAST=PAIRWISE
    PADJUST=BONFERRONI
  /MISSING CLASSMISSING=EXCLUDE
  /PRINT CPS DESCRIPTIVES MODELINFO FIT SUMMARY SOLUTION.
```

Generalized Linear Models

Model Information

Dependent Variable	Fura Ratio Magnitude
Probability Distribution	Normal
Link Function	Identity

Case Processing Summary

	N	Percent
Included	662	98.5%
Excluded	10	1.5%
Total	672	100.0%

Categorical Variable Information

			N	Percent
Factor	Condition	1	231	34.9%
		2	227	34.3%
		3	204	30.8%
		Total	662	100.0%
	Days	0	399	60.3%
		1	137	20.7%
		2	126	19.0%
		Total	662	100.0%

Continuous Variable Information

		N	Minimum	Maximum	Mean	Std. Deviation
Dependent Variable	Fura Ratio Magnitude	662	.02924	.81840	.3031525	.12371082

Goodness of Fit[a]

	Value	df	Value/df
Deviance	8.919	653	.014
Scaled Deviance	662.000	653	
Pearson Chi-Square	8.919	653	.014
Scaled Pearson Chi-Square	662.000	653	
Log Likelihood[b]	486.311		

Akaike's Information Criterion (AIC)	-952.621		
Finite Sample Corrected AIC (AICC)	-952.283		
Bayesian Information Criterion (BIC)	-907.669		
Consistent AIC (CAIC)	-897.669		

Dependent Variable: Fura Ratio Magnitude

Model: (Intercept), Condition, Days, Condition * Days

a. Information criteria are in smaller-is-better form.

b. The full log likelihood function is displayed and used in computing information criteria.

Omnibus Test[a]

Likelihood Ratio Chi-Square	df	Sig.
83.389	8	.000

Dependent Variable: Fura Ratio Magnitude

Model: (Intercept), Condition, Days, Condition * Days

a. Compares the fitted model against the intercept-only model.

Tests of Model Effects

Source		Type III	
	Wald Chi-Square	df	Sig.
(Intercept)	2926.350	1	.000
Condition	14.935	2	.001
Days	65.502	2	.000

Condition * Days	21.599	4	.000

Dependent Variable: Fura Ratio Magnitude

Model: (Intercept), Condition, Days, Condition * Days

Parameter Estimates

Parameter	B	Std. Error	95% Wald Confidence Interval		Hypothesis Test		
			Lower	Upper	Wald Chi-Square	df	Sig.
(Intercept)	.202	.0202	.162	.241	99.507	1	.000
[Condition=1]	.050	.0273	-.004	.103	3.338	1	.068
[Condition=2]	.071	.0257	.021	.122	7.704	1	.006
[Condition=3]	0[a]	.	.	.	.	.	.
[Days=0]	.138	.0228	.093	.182	36.688	1	.000
[Days=1]	.019	.0262	-.032	.071	.538	1	.463
[Days=2]	0[a]	.	.	.	.	.	.
[Condition=1] * [Days=0]	-.052	.0308	-.113	.008	2.860	1	.091
[Condition=1] * [Days=1]	.047	.0359	-.023	.117	1.715	1	.190
[Condition=1] * [Days=2]	0[a]	.	.	.	.	.	.
[Condition=2] * [Days=0]	-.094	.0295	-.152	-.036	10.184	1	.001
[Condition=2] * [Days=1]	-.011	.0360	-.081	.060	.086	1	.770
[Condition=2] * [Days=2]	0[a]	.	.	.	.	.	.
[Condition=3] * [Days=0]	0[a]	.	.	.	.	.	.
[Condition=3] * [Days=1]	0[a]	.	.	.	.	.	.
[Condition=3] * [Days=2]	0[a]	.	.	.	.	.	.
(Scale)	.013[b]	.0007	.012	.015			

Dependent Variable: Fura Ratio Magnitude

Model: (Intercept), Condition, Days, Condition * Days

a. Set to zero because this parameter is redundant.

b. Maximum likelihood estimate.

Estimated Marginal Means: Condition* Days

Estimates

Condition	Days	Mean	Std. Error	95% Wald Confidence Interval Lower	95% Wald Confidence Interval Upper
1	0	.3371120	.00980985	.3178850	.3563389
	1	.3177091	.01625330	.2858532	.3495649
	2	.2514258	.01835255	.2154554	.2873961
2	0	.3166400	.00995307	.2971324	.3361477
	1	.2817047	.01882932	.2447999	.3186095
	2	.2729942	.01594368	.2417451	.3042432
3	0	.3393861	.01046584	.3188734	.3598987
	1	.2208072	.01675351	.1879709	.2536435
	2	.2015559	.02020550	.1619539	.2411580

Pairwise Comparisons

(I) Condition*Days	(J) Condition*Days	Mean Difference (I-J)	Std. Error	df	Bonferroni Sig.	95% Wald Confidence Interval for Difference Lower	95% Wald Confidence Interval for Difference Upper
[Condition=1]*[Days=0]	[Condition=1]*[Days=1]	.0194029	.01898428	1	1.000	-.0412889	.0800947
	[Condition=1]*[Days=2]	.0856862[a]	.02080984	1	.001	.0191582	.1522142
	[Condition=2]*[Days=0]	.0204719	.01397486	1	1.000	-.0242050	.0651489
	[Condition=2]*[Days=1]	.0554073	.02123150	1	.326	-.0124688	.1232833
	[Condition=2]*[Days=2]	.0641178[a]	.01871989	1	.022	.0042712	.1239643
	[Condition=3]*[Days=0]	-.0022741	.01434458	1	1.000	-.0481330	.0435848

	[Condition=3]*[Days=1]	.1163047[a]	.01941426	1	.000	.0542383	.1783712
	[Condition=3]*[Days=2]	.1355560[a]	.02246097	1	.000	.0637494	.2073626
[Condition=1]*[Days=1]	[Condition=1]*[Days=0]	-.0194029	.01898428	1	1.000	-.0800947	.0412889
	[Condition=1]*[Days=2]	.0662833	.02451501	1	.247	-.0120900	.1446566
	[Condition=2]*[Days=0]	.0010690	.01905868	1	1.000	-.0598606	.0619987
	[Condition=2]*[Days=1]	.0360044	.02487394	1	1.000	-.0435164	.1155252
	[Condition=2]*[Days=2]	.0447149	.02276775	1	1.000	-.0280725	.1175023
	[Condition=3]*[Days=0]	-.0216770	.01933141	1	1.000	-.0834786	.0401245
	[Condition=3]*[Days=1]	.0969018[a]	.02334202	1	.001	.0222786	.1715251
	[Condition=3]*[Days=2]	.1161531[a]	.02593129	1	.000	.0332521	.1990542
[Condition=1]*[Days=2]	[Condition=1]*[Days=0]	-.0856862[a]	.02080984	1	.001	-.1522142	-.0191582
	[Condition=1]*[Days=1]	-.0662833	.02451501	1	.247	-.1446566	.0120900
	[Condition=2]*[Days=0]	-.0652143	.02087774	1	.064	-.1319593	.0015308
	[Condition=2]*[Days=1]	-.0302789	.02629372	1	1.000	-.1143386	.0537808
	[Condition=2]*[Days=2]	-.0215684	.02431084	1	1.000	-.0992890	.0561522
	[Condition=3]*[Days=0]	-.0879603[a]	.02112700	1	.001	-.1555022	-.0204183
	[Condition=3]*[Days=1]	.0306186	.02484948	1	1.000	-.0488240	.1100611
	[Condition=3]*[Days=2]	.0498699	.02729612	1	1.000	-.0373945	.1371342
[Condition=2]*[Days=0]	[Condition=1]*[Days=0]	-.0204719	.01397486	1	1.000	-.0651489	.0242050
	[Condition=1]*[Days=1]	-.0010690	.01905868	1	1.000	-.0619987	.0598606
	[Condition=1]*[Days=2]	.0652143	.02087774	1	.064	-.0015308	.1319593
	[Condition=2]*[Days=1]	.0349354	.02129805	1	1.000	-.0331534	.1030242
	[Condition=2]*[Days=2]	.0436459	.01879533	1	.728	-.0164419	.1037336
	[Condition=3]*[Days=0]	-.0227460	.01444290	1	1.000	-.0689193	.0234272
	[Condition=3]*[Days=1]	.0958328[a]	.01948702	1	.000	.0335338	.1581318
	[Condition=3]*[Days=2]	.1150841[a]	.02252389	1	.000	.0430764	.1870919
[Condition=2]*[Days=1]	[Condition=1]*[Days=0]	-.0554073	.02123150	1	.326	-.1232833	.0124688
	[Condition=1]*[Days=1]	-.0360044	.02487394	1	1.000	-.1155252	.0435164
	[Condition=1]*[Days=2]	.0302789	.02629372	1	1.000	-.0537808	.1143386
	[Condition=2]*[Days=0]	-.0349354	.02129805	1	1.000	-.1030242	.0331534

	[Condition=2]*[Days=2]	.0087105	.02467275	1	1.000	-.0701671	.0875880
	[Condition=3]*[Days=0]	-.0576814	.02154245	1	.267	-.1265515	.0111887
	[Condition=3]*[Days=1]	.0608974	.02520364	1	.565	-.0196774	.1414722
	[Condition=3]*[Days=2]	.0801487	.02761893	1	.134	-.0081476	.1684451
[Condition=2]*[Days=2]	[Condition=1]*[Days=0]	-.0641178[a]	.01871989	1	.022	-.1239643	-.0042712
	[Condition=1]*[Days=1]	-.0447149	.02276775	1	1.000	-.1175023	.0280725
	[Condition=1]*[Days=2]	.0215684	.02431084	1	1.000	-.0561522	.0992890
	[Condition=2]*[Days=0]	-.0436459	.01879533	1	.728	-.1037336	.0164419
	[Condition=2]*[Days=1]	-.0087105	.02467275	1	1.000	-.0875880	.0701671
	[Condition=3]*[Days=0]	-.0663919[a]	.01907183	1	.018	-.1273636	-.0054202
	[Condition=3]*[Days=1]	.0521870	.02312750	1	.865	-.0217505	.1261244
	[Condition=3]*[Days=2]	.0714383	.02573836	1	.198	-.0108460	.1537225
[Condition=3]*[Days=0]	[Condition=1]*[Days=0]	.0022741	.01434458	1	1.000	-.0435848	.0481330
	[Condition=1]*[Days=1]	.0216770	.01933141	1	1.000	-.0401245	.0834786
	[Condition=1]*[Days=2]	.0879603[a]	.02112700	1	.001	.0204183	.1555022
	[Condition=2]*[Days=0]	.0227460	.01444290	1	1.000	-.0234272	.0689193
	[Condition=2]*[Days=1]	.0576814	.02154245	1	.267	-.0111887	.1265515
	[Condition=2]*[Days=2]	.0663919[a]	.01907183	1	.018	.0054202	.1273636
	[Condition=3]*[Days=1]	.1185788[a]	.01975383	1	.000	.0554268	.1817309
	[Condition=3]*[Days=2]	.1378301[a]	.02275513	1	.000	.0650831	.2105772
[Condition=3]*[Days=1]	[Condition=1]*[Days=0]	-.1163047[a]	.01941426	1	.000	-.1783712	-.0542383
	[Condition=1]*[Days=1]	-.0969018[a]	.02334202	1	.001	-.1715251	-.0222786
	[Condition=1]*[Days=2]	-.0306186	.02484948	1	1.000	-.1100611	.0488240
	[Condition=2]*[Days=0]	-.0958328[a]	.01948702	1	.000	-.1581318	-.0335338
	[Condition=2]*[Days=1]	-.0608974	.02520364	1	.565	-.1414722	.0196774
	[Condition=2]*[Days=2]	-.0521870	.02312750	1	.865	-.1261244	.0217505
	[Condition=3]*[Days=0]	-.1185788[a]	.01975383	1	.000	-.1817309	-.0554268
	[Condition=3]*[Days=2]	.0192513	.02624771	1	1.000	-.0646613	.1031639
[Condition=3]*[Days=2]	[Condition=1]*[Days=0]	-.1355560[a]	.02246097	1	.000	-.2073626	-.0637494
	[Condition=1]*[Days=1]	-.1161531[a]	.02593129	1	.000	-.1990542	-.0332521

	[Condition=1]*[Days=2]	-.0498699	.02729612	1	1.000	-.1371342	.0373945
	[Condition=2]*[Days=0]	-.1150841[a]	.02252389	1	.000	-.1870919	-.0430764
	[Condition=2]*[Days=1]	-.0801487	.02761893	1	.134	-.1684451	.0081476
	[Condition=2]*[Days=2]	-.0714383	.02573836	1	.198	-.1537225	.0108460
	[Condition=3]*[Days=0]	-.1378301[a]	.02275513	1	.000	-.2105772	-.0650831
	[Condition=3]*[Days=1]	-.0192513	.02624771	1	1.000	-.1031639	.0646613

Pairwise comparisons of estimated marginal means based on the original scale of dependent variable Fura Ratio Magnitude

a. The mean difference is significant at the .05 level.

Overall Test Results

Wald Chi-Square	df	Sig.
88.868	8	.000

The Wald chi-square tests the effect of Condition*Days. This test is based on the linearly independent pairwise comparisons among the estimated marginal means.

Time to Peak Fura Ratio [ms]

```
MEANS TABLES=TTP BY Condition BY Days
  /CELLS=MEAN COUNT STDDEV MIN MAX.
```

Means

Case Processing Summary

	Cases					
	Included		Excluded		Total	
	N	Percent	N	Percent	N	Percent
Time to Peak * Condition * Days	662	98.5%	10	1.5%	672	100.0%

Report

Time to Peak

Condition	Days	Mean	N	Std. Deviation	Minimum	Maximum
1	0	69.8798055	140	18.49077433	24.71429	108.55556
	1	68.4734754	51	14.19687676	37.62500	105.83333
	2	63.8825479	40	18.76004719	32.83333	124.14286
	Total	68.5308309	231	17.74795847	24.71429	124.14286
2	0	73.8810543	136	18.43363571	26.53333	118.93750
	1	73.9220180	38	7.53946476	57.50000	87.29412
	2	69.5926604	53	21.77065926	29.93333	166.50000
	Total	72.8866567	227	18.01822531	26.53333	166.50000
3	0	69.6579366	123	15.70751223	29.07692	112.09677

	1	70.8155557	48	7.80704751	56.83333	92.00000
	2	77.2028743	33	9.37268878	59.52941	105.83333
	Total	71.1508222	204	13.54877271	29.07692	112.09677
Total	0	71.1752440	399	17.72292055	24.71429	118.93750
	1	70.8053329	137	10.74157254	37.62500	105.83333
	2	69.7730775	126	18.84549243	29.93333	166.50000
	Total	70.8318138	662	16.74255064	24.71429	166.50000

```
* Generalized Linear Models.
GENLIN TTP BY Condition Days (ORDER=ASCENDING)
  /MODEL Condition Days Condition*Days INTERCEPT=YES
 DISTRIBUTION=NORMAL LINK=IDENTITY
  /CRITERIA SCALE=MLE COVB=MODEL PCONVERGE=1E-006(ABSOLUTE) SINGULAR=1E-012 ANALYSISTYPE=3(WALD)
    CILEVEL=95 CITYPE=WALD LIKELIHOOD=FULL
  /EMMEANS TABLES=Condition*Days SCALE=ORIGINAL COMPARE=Condition*Days CONTRAST=PAIRWISE
    PADJUST=BONFERRONI
  /MISSING CLASSMISSING=EXCLUDE
  /PRINT CPS DESCRIPTIVES MODELINFO FIT SUMMARY SOLUTION.
```

Generalized Linear Models

Model Information

Dependent Variable	Time to Peak
Probability Distribution	Normal
Link Function	Identity

Case Processing Summary

	N	Percent
Included	662	98.5%
Excluded	10	1.5%
Total	672	100.0%

Categorical Variable Information

			N	Percent
Factor	Condition	1	231	34.9%
		2	227	34.3%
		3	204	30.8%
		Total	662	100.0%
	Days	0	399	60.3%
		1	137	20.7%
		2	126	19.0%
		Total	662	100.0%

Continuous Variable Information

		N	Minimum	Maximum	Mean	Std. Deviation
Dependent Variable	Time to Peak	662	24.71429	166.50000	70.8318138	16.74255064

Goodness of Fit[a]

	Value	df	Value/df
Deviance	179726.912	653	275.233
Scaled Deviance	662.000	653	
Pearson Chi-Square	179726.912	653	275.233
Scaled Pearson Chi-Square	662.000	653	
Log Likelihood[b]	-2794.238		

Akaike's Information Criterion (AIC)	5608.475		
Finite Sample Corrected AIC (AICC)	5608.813		
Bayesian Information Criterion (BIC)	5653.428		
Consistent AIC (CAIC)	5663.428		

Dependent Variable: Time to Peak

Model: (Intercept), Condition, Days, Condition * Days

a. Information criteria are in smaller-is-better form.

b. The full log likelihood function is displayed and used in computing information criteria.

Omnibus Test[a]

Likelihood Ratio Chi-Square	df	Sig.
20.169	8	.010

Dependent Variable: Time to Peak

Model: (Intercept), Condition, Days, Condition * Days

a. Compares the fitted model against the intercept-only model.

Tests of Model Effects

Source	Type III		
	Wald Chi-Square	df	Sig.
(Intercept)	9147.088	1	.000
Condition	10.823	2	.004
Days	.294	2	.863

Condition * Days	11.414	4	.022

Dependent Variable: Time to Peak

Model: (Intercept), Condition, Days, Condition * Days

Parameter Estimates

			95% Wald Confidence Interval		Hypothesis Test		
Parameter	B	Std. Error	Lower	Upper	Wald Chi-Square	df	Sig.
(Intercept)	77.203	2.8683	71.581	82.825	724.479	1	.000
[Condition=1]	-13.320	3.8748	-20.915	-5.726	11.818	1	.001
[Condition=2]	-7.610	3.6537	-14.771	-.449	4.338	1	.037
[Condition=3]	0[a]	.	.	.	.	.	.
[Days=0]	-7.545	3.2302	-13.876	-1.214	5.456	1	.020
[Days=1]	-6.387	3.7260	-13.690	.916	2.939	1	.086
[Days=2]	0[a]	.	.	.	.	.	.
[Condition=1] * [Days=0]	13.542	4.3773	4.963	22.122	9.571	1	.002
[Condition=1] * [Days=1]	10.978	5.0984	.986	20.971	4.637	1	.031
[Condition=1] * [Days=2]	0[a]	.	.	.	.	.	.
[Condition=2] * [Days=0]	11.833	4.1896	3.622	20.045	7.977	1	.005
[Condition=2] * [Days=1]	10.717	5.1137	.694	20.739	4.392	1	.036
[Condition=2] * [Days=2]	0[a]	.	.	.	.	.	.
[Condition=3] * [Days=0]	0[a]	.	.	.	.	.	.
[Condition=3] * [Days=1]	0[a]	.	.	.	.	.	.
[Condition=3] * [Days=2]	0[a]	.	.	.	.	.	.
(Scale)	271.491[b]	14.9225	243.764	302.372			

Dependent Variable: Time to Peak

Model: (Intercept), Condition, Days, Condition * Days

a. Set to zero because this parameter is redundant.

b. Maximum likelihood estimate.

Estimated Marginal Means: Condition* Days

Estimates

Condition	Days	Mean	Std. Error	95% Wald Confidence Interval Lower	Upper
1	0	69.8798055	1.39255880	67.1504404	72.6091706
	1	68.4734754	2.30723841	63.9513712	72.9955796
	2	63.8825479	2.60523897	58.7763733	68.9887224
2	0	73.8810543	1.41288921	71.1118423	76.6502662
	1	73.9220180	2.67291878	68.6831934	79.1608425
	2	69.5926604	2.26328699	65.1566994	74.0286214
3	0	69.6579366	1.48567907	66.7460592	72.5698141
	1	70.8155557	2.37824692	66.1542774	75.4768340
	2	77.2028743	2.86827370	71.5811611	82.8245875

Pairwise Comparisons

(I) Condition*Days	(J) Condition*Days	Mean Difference (I-J)	Std. Error	df	Bonferroni Sig.	95% Wald Confidence Interval for Difference Lower	Upper
[Condition=1]*[Days=0]	[Condition=1]*[Days=1]	1.4063301	2.69491542	1	1.000	-7.2091803	10.0218406
	[Condition=1]*[Days=2]	5.9972576	2.95406332	1	1.000	-3.4467358	15.4412510
	[Condition=2]*[Days=0]	-4.0012488	1.98380340	1	1.000	-10.3433695	2.3408719
	[Condition=2]*[Days=1]	-4.0422125	3.01392018	1	1.000	-13.6775653	5.5931403
	[Condition=2]*[Days=2]	.2871451	2.65738368	1	1.000	-8.2083783	8.7826684
	[Condition=3]*[Days=0]	.2218689	2.03628641	1	1.000	-6.2880374	6.7317751

	[Condition=3]*[Days=1]	-.9357502	2.75595327	1	1.000	-9.7463957	7.8748952
	[Condition=3]*[Days=2]	-7.3230688	3.18845010	1	.779	-17.5163851	2.8702475
[Condition=1]*[Days=1]	[Condition=1]*[Days=0]	-1.4063301	2.69491542	1	1.000	-10.0218406	7.2091803
	[Condition=1]*[Days=2]	4.5909275	3.48003149	1	1.000	-6.5345600	15.7164150
	[Condition=2]*[Days=0]	-5.4075789	2.70547685	1	1.000	-14.0568538	3.2416959
	[Condition=2]*[Days=1]	-5.4485426	3.53098342	1	1.000	-16.7369209	5.8398356
	[Condition=2]*[Days=2]	-1.1191850	3.23199893	1	1.000	-11.4517247	9.2133547
	[Condition=3]*[Days=0]	-1.1844613	2.74419230	1	1.000	-9.9575075	7.5885849
	[Condition=3]*[Days=1]	-2.3420803	3.31351890	1	1.000	-12.9352353	8.2510747
	[Condition=3]*[Days=2]	-8.7293989	3.68107907	1	.638	-20.4976255	3.0388276
[Condition=1]*[Days=2]	[Condition=1]*[Days=0]	-5.9972576	2.95406332	1	1.000	-15.4412510	3.4467358
	[Condition=1]*[Days=1]	-4.5909275	3.48003149	1	1.000	-15.7164150	6.5345600
	[Condition=2]*[Days=0]	-9.9985064[a]	2.96370140	1	.027	-19.4733123	-.5237005
	[Condition=2]*[Days=1]	-10.0394701	3.73252795	1	.257	-21.9721762	1.8932360
	[Condition=2]*[Days=2]	-5.7101125	3.45104884	1	1.000	-16.7429439	5.3227189
	[Condition=3]*[Days=0]	-5.7753887	2.99908526	1	1.000	-15.3633151	3.8125376
	[Condition=3]*[Days=1]	-6.9330078	3.52751024	1	1.000	-18.2102825	4.3442668
	[Condition=3]*[Days=2]	-13.3203264[a]	3.87482440	1	.021	-25.7079472	-.9327057
[Condition=2]*[Days=0]	[Condition=1]*[Days=0]	4.0012488	1.98380340	1	1.000	-2.3408719	10.3433695
	[Condition=1]*[Days=1]	5.4075789	2.70547685	1	1.000	-3.2416959	14.0568538
	[Condition=1]*[Days=2]	9.9985064[a]	2.96370140	1	.027	.5237005	19.4733123
	[Condition=2]*[Days=1]	-.0409637	3.02336745	1	1.000	-9.7065190	9.6245916
	[Condition=2]*[Days=2]	4.2883939	2.66809368	1	1.000	-4.2413688	12.8181566
	[Condition=3]*[Days=0]	4.2231177	2.05024345	1	1.000	-2.3314086	10.7776439
	[Condition=3]*[Days=1]	3.0654986	2.76628168	1	1.000	-5.7781663	11.9091634
	[Condition=3]*[Days=2]	-3.3218200	3.19738173	1	1.000	-13.5436903	6.9000503
[Condition=2]*[Days=1]	[Condition=1]*[Days=0]	4.0422125	3.01392018	1	1.000	-5.5931403	13.6775653
	[Condition=1]*[Days=1]	5.4485426	3.53098342	1	1.000	-5.8398356	16.7369209
	[Condition=1]*[Days=2]	10.0394701	3.73252795	1	.257	-1.8932360	21.9721762
	[Condition=2]*[Days=0]	.0409637	3.02336745	1	1.000	-9.6245916	9.7065190

	[Condition=2]*[Days=2]	4.3293576	3.50242242	1	1.000	-6.8677126	15.5264278
	[Condition=3]*[Days=0]	4.2640814	3.05806101	1	1.000	-5.5123875	14.0405502
	[Condition=3]*[Days=1]	3.1064623	3.57778608	1	1.000	-8.3315418	14.5444663
	[Condition=3]*[Days=2]	-3.2808563	3.92064904	1	1.000	-15.8149761	9.2532635
[Condition=2]*[Days=2]	[Condition=1]*[Days=0]	-.2871451	2.65738368	1	1.000	-8.7826684	8.2083783
	[Condition=1]*[Days=1]	1.1191850	3.23199893	1	1.000	-9.2133547	11.4517247
	[Condition=1]*[Days=2]	5.7101125	3.45104884	1	1.000	-5.3227189	16.7429439
	[Condition=2]*[Days=0]	-4.2883939	2.66809368	1	1.000	-12.8181566	4.2413688
	[Condition=2]*[Days=1]	-4.3293576	3.50242242	1	1.000	-15.5264278	6.8677126
	[Condition=3]*[Days=0]	-.0652762	2.70734377	1	1.000	-8.7205195	8.5899671
	[Condition=3]*[Days=1]	-1.2228953	3.28306661	1	1.000	-11.7186959	9.2729052
	[Condition=3]*[Days=2]	-7.6102139	3.65369156	1	1.000	-19.2908840	4.0704562
[Condition=3]*[Days=0]	[Condition=1]*[Days=0]	-.2218689	2.03628641	1	1.000	-6.7317751	6.2880374
	[Condition=1]*[Days=1]	1.1844613	2.74419230	1	1.000	-7.5885849	9.9575075
	[Condition=1]*[Days=2]	5.7753887	2.99908526	1	1.000	-3.8125376	15.3633151
	[Condition=2]*[Days=0]	-4.2231177	2.05024345	1	1.000	-10.7776439	2.3314086
	[Condition=2]*[Days=1]	-4.2640814	3.05806101	1	1.000	-14.0405502	5.5123875
	[Condition=2]*[Days=2]	.0652762	2.70734377	1	1.000	-8.5899671	8.7205195
	[Condition=3]*[Days=1]	-1.1576191	2.80415775	1	1.000	-10.1223719	7.8071337
	[Condition=3]*[Days=2]	-7.5449377	3.23020686	1	.702	-17.8717482	2.7818729
[Condition=3]*[Days=1]	[Condition=1]*[Days=0]	.9357502	2.75595327	1	1.000	-7.8748952	9.7463957
	[Condition=1]*[Days=1]	2.3420803	3.31351890	1	1.000	-8.2510747	12.9352353
	[Condition=1]*[Days=2]	6.9330078	3.52751024	1	1.000	-4.3442668	18.2102825
	[Condition=2]*[Days=0]	-3.0654986	2.76628168	1	1.000	-11.9091634	5.7781663
	[Condition=2]*[Days=1]	-3.1064623	3.57778608	1	1.000	-14.5444663	8.3315418
	[Condition=2]*[Days=2]	1.2228953	3.28306661	1	1.000	-9.2729052	11.7186959
	[Condition=3]*[Days=0]	1.1576191	2.80415775	1	1.000	-7.8071337	10.1223719
	[Condition=3]*[Days=2]	-6.3873186	3.72599684	1	1.000	-18.2991450	5.5245079
[Condition=3]*[Days=2]	[Condition=1]*[Days=0]	7.3230688	3.18845010	1	.779	-2.8702475	17.5163851
	[Condition=1]*[Days=1]	8.7293989	3.68107907	1	.638	-3.0388276	20.4976255

	[Condition=1]*[Days=2]	13.3203264[a]	3.87482440	1	.021	.9327057	25.7079472
	[Condition=2]*[Days=0]	3.3218200	3.19738173	1	1.000	-6.9000503	13.5436903
	[Condition=2]*[Days=1]	3.2808563	3.92064904	1	1.000	-9.2532635	15.8149761
	[Condition=2]*[Days=2]	7.6102139	3.65369156	1	1.000	-4.0704562	19.2908840
	[Condition=3]*[Days=0]	7.5449377	3.23020686	1	.702	-2.7818729	17.8717482
	[Condition=3]*[Days=1]	6.3873186	3.72599684	1	1.000	-5.5245079	18.2991450

Pairwise comparisons of estimated marginal means based on the original scale of dependent variable Time to Peak

a. The mean difference is significant at the .05 level.

Overall Test Results

Wald Chi-Square	df	Sig.
20.479	8	.009

The Wald chi-square tests the effect of Condition*Days. This test is based on the linearly independent pairwise comparisons among the estimated marginal means.

Tau of Fura Ratio Decline [s]

```
MEANS TABLES=Fura_tau BY Condition BY Days
  /CELLS=MEAN COUNT STDDEV MIN MAX.
```

Means

Case Processing Summary

	Cases					
	Included		Excluded		Total	
	N	Percent	N	Percent	N	Percent
tau of Fura Decline * Condition * Days	662	98.5%	10	1.5%	672	100.0%

Report

tau of Fura Decline

Condition	Days	Mean	N	Std. Deviation	Minimum	Maximum
1	0	.2935984	140	.07883380	.09643	.63117
	1	.4080736	51	.17492531	.20000	1.04089
	2	.3772529	40	.16066347	.19343	.94545
	Total	.3333578	231	.13160519	.09643	1.04089
2	0	.2791872	136	.06245089	.11325	.46964
	1	.4726517	38	.21795347	.24997	1.41427
	2	.5318214	53	.22187440	.23732	1.09600
	Total	.3705584	227	.18522679	.11325	1.41427
3	0	.3530741	123	.15150830	.13116	1.33762

	1	.5871118	48	.23434505	.23970	1.27217
	2	.6909448	33	.40847768	.22500	1.82147
	Total	.4627973	204	.26874314	.13116	1.82147
Total	0	.3070209	399	.10730017	.09643	1.33762
	1	.4887145	137	.22154040	.20000	1.41427
	2	.5244272	126	.29244118	.19343	1.82147
	Total	.3860016	662	.20690093	.09643	1.82147

```
* Generalized Linear Models.
GENLIN Fura_tau BY Condition Days (ORDER=ASCENDING)
  /MODEL Condition Days Condition*Days INTERCEPT=YES
 DISTRIBUTION=NORMAL LINK=IDENTITY
  /CRITERIA SCALE=MLE COVB=MODEL PCONVERGE=1E-006(ABSOLUTE) SINGULAR=1E-012 ANALYSISTYPE=3(WALD)
    CILEVEL=95 CITYPE=WALD LIKELIHOOD=FULL
  /EMMEANS TABLES=Condition*Days SCALE=ORIGINAL COMPARE=Condition*Days CONTRAST=PAIRWISE
    PADJUST=BONFERRONI
  /MISSING CLASSMISSING=EXCLUDE
  /PRINT CPS DESCRIPTIVES MODELINFO FIT SUMMARY SOLUTION.
```

Generalized Linear Models

Model Information

Dependent Variable	tau of Fura Decline
Probability Distribution	Normal
Link Function	Identity

Case Processing Summary

	N	Percent
Included	662	98.5%
Excluded	10	1.5%
Total	672	100.0%

Categorical Variable Information

			N	Percent
Factor	Condition	1	231	34.9%
		2	227	34.3%
		3	204	30.8%
		Total	662	100.0%
	Days	0	399	60.3%
		1	137	20.7%
		2	126	19.0%
		Total	662	100.0%

Continuous Variable Information

		N	Minimum	Maximum	Mean	Std. Deviation
Dependent Variable	tau of Fura Decline	662	.09643	1.82147	.3860016	.20690093

Goodness of Fit[a]

	Value	df	Value/df
Deviance	18.965	653	.029
Scaled Deviance	662.000	653	
Pearson Chi-Square	18.965	653	.029
Scaled Pearson Chi-Square	662.000	653	
Log Likelihood[b]	236.589		

Akaike's Information Criterion (AIC)	-453.177		
Finite Sample Corrected AIC (AICC)	-452.839		
Bayesian Information Criterion (BIC)	-408.225		
Consistent AIC (CAIC)	-398.225		

Dependent Variable: tau of Fura Decline

Model: (Intercept), Condition, Days, Condition * Days

a. Information criteria are in smaller-is-better form.

b. The full log likelihood function is displayed and used in computing information criteria.

Omnibus Test[a]

Likelihood Ratio Chi-Square	df	Sig.
264.869	8	.000

Dependent Variable: tau of Fura Decline

Model: (Intercept), Condition, Days, Condition * Days

a. Compares the fitted model against the intercept-only model.

Tests of Model Effects

Source	Type III Wald Chi-Square	df	Sig.
(Intercept)	3403.992	1	.000
Condition	97.083	2	.000
Days	225.224	2	.000

Condition * Days	37.037	4	.000

Dependent Variable: tau of Fura Decline

Model: (Intercept), Condition, Days, Condition * Days

Parameter Estimates

Parameter	B	Std. Error	95% Wald Confidence Interval Lower	95% Wald Confidence Interval Upper	Hypothesis Test Wald Chi-Square	Hypothesis Test df	Hypothesis Test Sig.
(Intercept)	.691	.0295	.633	.749	549.915	1	.000
[Condition=1]	-.314	.0398	-.392	-.236	62.109	1	.000
[Condition=2]	-.159	.0375	-.233	-.086	17.974	1	.000
[Condition=3]	0[a]	.	.	.	.	.	.
[Days=0]	-.338	.0332	-.403	-.273	103.679	1	.000
[Days=1]	-.104	.0383	-.179	-.029	7.359	1	.007
[Days=2]	0[a]	.	.	.	.	.	.
[Condition=1] * [Days=0]	.254	.0450	.166	.342	31.963	1	.000
[Condition=1] * [Days=1]	.135	.0524	.032	.237	6.610	1	.010
[Condition=1] * [Days=2]	0[a]	.	.	.	.	.	.
[Condition=2] * [Days=0]	.085	.0430	.001	.170	3.922	1	.048
[Condition=2] * [Days=1]	.045	.0525	-.058	.148	.723	1	.395
[Condition=2] * [Days=2]	0[a]	.	.	.	.	.	.
[Condition=3] * [Days=0]	0[a]	.	.	.	.	.	.
[Condition=3] * [Days=1]	0[a]	.	.	.	.	.	.
[Condition=3] * [Days=2]	0[a]	.	.	.	.	.	.
(Scale)	.029[b]	.0016	.026	.032			

Dependent Variable: tau of Fura Decline

Model: (Intercept), Condition, Days, Condition * Days

a. Set to zero because this parameter is redundant.

b. Maximum likelihood estimate.

Estimated Marginal Means: Condition* Days

Estimates

Condition	Days	Mean	Std. Error	95% Wald Confidence Interval Lower	95% Wald Confidence Interval Upper
1	0	.2935984	.01430502	.2655611	.3216358
	1	.4080736	.02370105	.3616204	.4545268
	2	.3772529	.02676225	.3247999	.4297060
2	0	.2791872	.01451387	.2507405	.3076338
	1	.4726517	.02745749	.4188361	.5264674
	2	.5318214	.02324956	.4862531	.5773897
3	0	.3530741	.01526160	.3231619	.3829863
	1	.5871118	.02443048	.5392289	.6349946
	2	.6909448	.02946427	.6331959	.7486937

Pairwise Comparisons

(I) Condition*Days	(J) Condition*Days	Mean Difference (I-J)	Std. Error	df	Bonferroni Sig.	95% Wald Confidence Interval for Difference Lower	95% Wald Confidence Interval for Difference Upper
[Condition=1]*[Days=0]	[Condition=1]*[Days=1]	-.1144752[a]	.02768345	1	.001	-.2029778	-.0259726
	[Condition=1]*[Days=2]	-.0836545	.03034554	1	.210	-.1806677	.0133587
	[Condition=2]*[Days=0]	.0144112	.02037857	1	1.000	-.0507380	.0795605
	[Condition=2]*[Days=1]	-.1790533[a]	.03096042	1	.000	-.2780322	-.0800744
	[Condition=2]*[Days=2]	-.2382230[a]	.02729790	1	.000	-.3254930	-.1509529
	[Condition=3]*[Days=0]	-.0594757	.02091770	1	.161	-.1263485	.0073972

	[Condition=3]*[Days=1]	-.2935133[a]	.02831046	1	.000	-.3840205	-.2030062
	[Condition=3]*[Days=2]	-.3973464[a]	.03275327	1	.000	-.5020569	-.2926358
[Condition=1]*[Days=1]	[Condition=1]*[Days=0]	.1144752[a]	.02768345	1	.001	.0259726	.2029778
	[Condition=1]*[Days=2]	.0308207	.03574853	1	1.000	-.0834656	.1451070
	[Condition=2]*[Days=0]	.1288864[a]	.02779194	1	.000	.0400370	.2177359
	[Condition=2]*[Days=1]	-.0645781	.03627193	1	1.000	-.1805377	.0513815
	[Condition=2]*[Days=2]	-.1237478[a]	.03320062	1	.007	-.2298885	-.0176070
	[Condition=3]*[Days=0]	.0549995	.02818964	1	1.000	-.0351214	.1451204
	[Condition=3]*[Days=1]	-.1790381[a]	.03403804	1	.000	-.2878560	-.0702202
	[Condition=3]*[Days=2]	-.2828711[a]	.03781379	1	.000	-.4037599	-.1619823
[Condition=1]*[Days=2]	[Condition=1]*[Days=0]	.0836545	.03034554	1	.210	-.0133587	.1806677
	[Condition=1]*[Days=1]	-.0308207	.03574853	1	1.000	-.1451070	.0834656
	[Condition=2]*[Days=0]	.0980657[a]	.03044455	1	.046	.0007360	.1953954
	[Condition=2]*[Days=1]	-.0953988	.03834230	1	.462	-.2179772	.0271796
	[Condition=2]*[Days=2]	-.1545685[a]	.03545081	1	.000	-.2679030	-.0412340
	[Condition=3]*[Days=0]	.0241788	.03080802	1	1.000	-.0743129	.1226705
	[Condition=3]*[Days=1]	-.2098589[a]	.03623626	1	.000	-.3257044	-.0940133
	[Condition=3]*[Days=2]	-.3136919[a]	.03980403	1	.000	-.4409434	-.1864404
[Condition=2]*[Days=0]	[Condition=1]*[Days=0]	-.0144112	.02037857	1	1.000	-.0795605	.0507380
	[Condition=1]*[Days=1]	-.1288864[a]	.02779194	1	.000	-.2177359	-.0400370
	[Condition=1]*[Days=2]	-.0980657[a]	.03044455	1	.046	-.1953954	-.0007360
	[Condition=2]*[Days=1]	-.1934646[a]	.03105746	1	.000	-.2927537	-.0941754
	[Condition=2]*[Days=2]	-.2526342[a]	.02740792	1	.000	-.3402560	-.1650124
	[Condition=3]*[Days=0]	-.0738869[a]	.02106107	1	.016	-.1412181	-.0065557
	[Condition=3]*[Days=1]	-.3079246[a]	.02841656	1	.000	-.3987709	-.2170783
	[Condition=3]*[Days=2]	-.4117576[a]	.03284502	1	.000	-.5167615	-.3067537
[Condition=2]*[Days=1]	[Condition=1]*[Days=0]	.1790533[a]	.03096042	1	.000	.0800744	.2780322
	[Condition=1]*[Days=1]	.0645781	.03627193	1	1.000	-.0513815	.1805377
	[Condition=1]*[Days=2]	.0953988	.03834230	1	.462	-.0271796	.2179772
	[Condition=2]*[Days=0]	.1934646[a]	.03105746	1	.000	.0941754	.2927537

	[Condition=2]*[Days=2]	-.0591697	.03597854	1	1.000	-.1741913	.0558520
	[Condition=3]*[Days=0]	.1195776[a]	.03141385	1	.005	.0191491	.2200062
	[Condition=3]*[Days=1]	-.1144600	.03675271	1	.066	-.2319566	.0030366
	[Condition=3]*[Days=2]	-.2182930[a]	.04027476	1	.000	-.3470495	-.0895366
[Condition=2]*[Days=2]	[Condition=1]*[Days=0]	.2382230[a]	.02729790	1	.000	.1509529	.3254930
	[Condition=1]*[Days=1]	.1237478[a]	.03320062	1	.007	.0176070	.2298885
	[Condition=1]*[Days=2]	.1545685[a]	.03545081	1	.000	.0412340	.2679030
	[Condition=2]*[Days=0]	.2526342[a]	.02740792	1	.000	.1650124	.3402560
	[Condition=2]*[Days=1]	.0591697	.03597854	1	1.000	-.0558520	.1741913
	[Condition=3]*[Days=0]	.1787473[a]	.02781112	1	.000	.0898365	.2676581
	[Condition=3]*[Days=1]	-.0552904	.03372522	1	1.000	-.1631082	.0525275
	[Condition=3]*[Days=2]	-.1591234[a]	.03753245	1	.001	-.2791128	-.0391340
[Condition=3]*[Days=0]	[Condition=1]*[Days=0]	.0594757	.02091770	1	.161	-.0073972	.1263485
	[Condition=1]*[Days=1]	-.0549995	.02818964	1	1.000	-.1451204	.0351214
	[Condition=1]*[Days=2]	-.0241788	.03080802	1	1.000	-.1226705	.0743129
	[Condition=2]*[Days=0]	.0738869[a]	.02106107	1	.016	.0065557	.1412181
	[Condition=2]*[Days=1]	-.1195776[a]	.03141385	1	.005	-.2200062	-.0191491
	[Condition=2]*[Days=2]	-.1787473[a]	.02781112	1	.000	-.2676581	-.0898365
	[Condition=3]*[Days=1]	-.2340377[a]	.02880564	1	.000	-.3261278	-.1419475
	[Condition=3]*[Days=2]	-.3378707[a]	.03318222	1	.000	-.4439526	-.2317888
[Condition=3]*[Days=1]	[Condition=1]*[Days=0]	.2935133[a]	.02831046	1	.000	.2030062	.3840205
	[Condition=1]*[Days=1]	.1790381[a]	.03403804	1	.000	.0702202	.2878560
	[Condition=1]*[Days=2]	.2098589[a]	.03623626	1	.000	.0940133	.3257044
	[Condition=2]*[Days=0]	.3079246[a]	.02841656	1	.000	.2170783	.3987709
	[Condition=2]*[Days=1]	.1144600	.03675271	1	.066	-.0030366	.2319566
	[Condition=2]*[Days=2]	.0552904	.03372522	1	1.000	-.0525275	.1631082
	[Condition=3]*[Days=0]	.2340377[a]	.02880564	1	.000	.1419475	.3261278
	[Condition=3]*[Days=2]	-.1038330	.03827521	1	.240	-.2261969	.0185309
[Condition=3]*[Days=2]	[Condition=1]*[Days=0]	.3973464[a]	.03275327	1	.000	.2926358	.5020569
	[Condition=1]*[Days=1]	.2828711[a]	.03781379	1	.000	.1619823	.4037599

	[Condition=1]*[Days=2]	.3136919[a]	.03980403	1	.000	.1864404	.4409434
	[Condition=2]*[Days=0]	.4117576[a]	.03284502	1	.000	.3067537	.5167615
	[Condition=2]*[Days=1]	.2182930[a]	.04027476	1	.000	.0895366	.3470495
	[Condition=2]*[Days=2]	.1591234[a]	.03753245	1	.001	.0391340	.2791128
	[Condition=3]*[Days=0]	.3378707[a]	.03318222	1	.000	.2317888	.4439526
	[Condition=3]*[Days=1]	.1038330	.03827521	1	.240	-.0185309	.2261969

Pairwise comparisons of estimated marginal means based on the original scale of dependent variable tau of Fura Decline

a. The mean difference is significant at the .05 level.

Overall Test Results

Wald Chi-Square	df	Sig.
325.691	8	.000

The Wald chi-square tests the effect of Condition*Days. This test is based on the linearly independent pairwise comparisons among the estimated marginal means.

Baseline (Diastolic) Sarcomere Length [µm]

```
DATASET ACTIVATE DataSet5.
MEANS TABLES=BaselineSL BY Condition BY Day
  /CELLS=MEAN COUNT STDDEV MIN MAX.
```

Means

```
[DataSet5] C:\Lab\charles\Projects\Myocyte\01 polaxamer\6 appendix stats data\sl_for_suppl.sav
```

Case Processing Summary

	Cases					
	Included		Excluded		Total	
	N	Percent	N	Percent	N	Percent
Baseline Diastolic SL * Condition * Day	656	97.6%	16	2.4%	672	100.0%

Report

Baseline Diastolic SL

Condition	Day	Mean	N	Std. Deviation	Minimum	Maximum
1	0	1.7682456	139	.06317064	1.54964	1.92994
	1	1.7639352	51	.05138318	1.64196	1.86995
	2	1.7680987	39	.05439820	1.61064	1.86112
	Total	1.7672606	229	.05908213	1.54964	1.92994
2	0	1.7571244	133	.06366971	1.55125	1.89175

	1	1.7746257	46	.05356551	1.64403	1.88988
	2	1.7302446	41	.08279795	1.34383	1.84031
	Total	1.7557744	220	.06695445	1.34383	1.89175
3	0	1.7612748	122	.05054115	1.58937	1.87963
	1	1.7535462	48	.04514182	1.62526	1.83409
	2	1.7165625	37	.08126459	1.51015	1.84003
	Total	1.7514906	207	.05827711	1.51015	1.87963
Total	0	1.7623330	394	.05975962	1.54964	1.92994
	1	1.7638876	145	.05050728	1.62526	1.88988
	2	1.7385358	117	.07651783	1.34383	1.86112
	Total	1.7584323	656	.06186210	1.34383	1.92994

```
* Generalized Linear Models.
GENLIN BaselineSL BY Condition Day (ORDER=ASCENDING)
  /MODEL Condition Day Condition*Day INTERCEPT=YES
 DISTRIBUTION=NORMAL LINK=IDENTITY
  /CRITERIA SCALE=MLE COVB=MODEL PCONVERGE=1E-006(ABSOLUTE) SINGULAR=1E-012 ANALYSISTYPE=3(WALD)
    CILEVEL=95 CITYPE=WALD LIKELIHOOD=FULL
  /EMMEANS TABLES=Condition*Day SCALE=ORIGINAL COMPARE=Condition*Day CONTRAST=PAIRWISE
    PADJUST=BONFERRONI
  /MISSING CLASSMISSING=EXCLUDE
  /PRINT CPS DESCRIPTIVES MODELINFO FIT SUMMARY SOLUTION.
```

Generalized Linear Models

Model Information

Dependent Variable	Baseline Diastolic SL
Probability Distribution	Normal

Link Function	Identity

Case Processing Summary

	N	Percent
Included	656	97.6%
Excluded	16	2.4%
Total	672	100.0%

Categorical Variable Information

			N	Percent
Factor	Condition	1	229	34.9%
		2	220	33.5%
		3	207	31.6%
		Total	656	100.0%
	Day	0	394	60.1%
		1	145	22.1%
		2	117	17.8%
		Total	656	100.0%

Continuous Variable Information

		N	Minimum	Maximum	Mean	Std. Deviation
Dependent Variable	Baseline Diastolic SL	656	1.34383	1.92994	1.7584323	.06186210

Goodness of Fit[a]

	Value	df	Value/df

Deviance	2.376	647	.004
Scaled Deviance	656.000	647	
Pearson Chi-Square	2.376	647	.004
Scaled Pearson Chi-Square	656.000	647	
Log Likelihood[b]	912.753		
Akaike's Information Criterion (AIC)	-1805.505		
Finite Sample Corrected AIC (AICC)	-1805.164		
Bayesian Information Criterion (BIC)	-1760.644		
Consistent AIC (CAIC)	-1750.644		

Dependent Variable: Baseline Diastolic SL

Model: (Intercept), Condition, Day, Condition * Day

a. Information criteria are in smaller-is-better form.

b. The full log likelihood function is displayed and used in computing information criteria.

Omnibus Test[a]

Likelihood Ratio Chi-Square	df	Sig.
35.056	8	.000

Dependent Variable: Baseline Diastolic SL

Model: (Intercept), Condition, Day, Condition * Day

a. Compares the fitted model against the intercept-only model.

Tests of Model Effects

Source	Type III Wald Chi-Square	df	Sig.
(Intercept)	424692.272	1	.000
Condition	12.162	2	.002
Day	15.871	2	.000
Condition * Day	12.538	4	.014

Dependent Variable: Baseline Diastolic SL

Model: (Intercept), Condition, Day, Condition * Day

Parameter Estimates

Parameter	B	Std. Error	95% Wald Confidence Interval		Hypothesis Test		
			Lower	Upper	Wald Chi-Square	df	Sig.
(Intercept)	1.717	.0099	1.697	1.736	30098.335	1	.000
[Condition=1]	.052	.0138	.024	.079	13.922	1	.000
[Condition=2]	.014	.0136	-.013	.040	1.005	1	.316
[Condition=3]	0[a]	.	.	.	.	.	.
[Day=0]	.045	.0113	.023	.067	15.669	1	.000
[Day=1]	.037	.0132	.011	.063	7.890	1	.005
[Day=2]	0[a]	.	.	.	.	.	.
[Condition=1] * [Day=0]	-.045	.0157	-.075	-.014	8.056	1	.005
[Condition=1] * [Day=1]	-.041	.0184	-.077	-.005	5.020	1	.025
[Condition=1] * [Day=2]	0[a]	.	.	.	.	.	.
[Condition=2] * [Day=0]	-.018	.0156	-.048	.013	1.308	1	.253
[Condition=2] * [Day=1]	.007	.0185	-.029	.044	.161	1	.688
[Condition=2] * [Day=2]	0[a]	.	.	.	.	.	.
[Condition=3] * [Day=0]	0[a]	.	.	.	.	.	.
[Condition=3] * [Day=1]	0[a]	.	.	.	.	.	.
[Condition=3] * [Day=2]	0[a]	.	.	.	.	.	.
(Scale)	.004[b]	.0002	.003	.004			

Dependent Variable: Baseline Diastolic SL

Model: (Intercept), Condition, Day, Condition * Day

a. Set to zero because this parameter is redundant.

b. Maximum likelihood estimate.

Estimated Marginal Means: Condition* Day

Estimates

Condition	Day	Mean	Std. Error	95% Wald Confidence Interval Lower	95% Wald Confidence Interval Upper
1	0	1.7682456	.00510484	1.7582403	1.7782509
	1	1.7639352	.00842760	1.7474174	1.7804530
	2	1.7680987	.00963733	1.7492098	1.7869875
2	0	1.7571244	.00521871	1.7468959	1.7673529
	1	1.7746257	.00887381	1.7572333	1.7920180
	2	1.7302446	.00939934	1.7118223	1.7486670
3	0	1.7612748	.00544891	1.7505952	1.7719545
	1	1.7535462	.00868698	1.7365200	1.7705724
	2	1.7165625	.00989438	1.6971699	1.7359552

Pairwise Comparisons

(I) Condition*Day	(J) Condition*Day	Mean Difference (I-J)	Std. Error	df	Bonferroni Sig.	95% Wald Confidence Interval for Difference Lower	95% Wald Confidence Interval for Difference Upper
[Condition=1]*[Day=0]	[Condition=1]*[Day=1]	.0043104	.00985312	1	1.000	-.0271896	.0358103

	[Condition=1]*[Day=2]	.0001469	.01090585	1	1.000	-.0347185	.0350124
	[Condition=2]*[Day=0]	.0111212	.00730030	1	1.000	-.0122175	.0344599
	[Condition=2]*[Day=1]	-.0063801	.01023738	1	1.000	-.0391085	.0263483
	[Condition=2]*[Day=2]	.0380010[a]	.01069612	1	.014	.0038060	.0721959
	[Condition=3]*[Day=0]	.0069708	.00746659	1	1.000	-.0168995	.0308411
	[Condition=3]*[Day=1]	.0146994	.01007586	1	1.000	-.0175126	.0469114
	[Condition=3]*[Day=2]	.0516831[a]	.01113364	1	.000	.0160894	.0872768
[Condition=1]*[Day=1]	[Condition=1]*[Day=0]	-.0043104	.00985312	1	1.000	-.0358103	.0271896
	[Condition=1]*[Day=2]	-.0041634	.01280245	1	1.000	-.0450922	.0367654
	[Condition=2]*[Day=0]	.0068108	.00991259	1	1.000	-.0248793	.0385009
	[Condition=2]*[Day=1]	-.0106904	.01223802	1	1.000	-.0498148	.0284339
	[Condition=2]*[Day=2]	.0336906	.01262427	1	.274	-.0066685	.0740498
	[Condition=3]*[Day=0]	.0026604	.01003569	1	1.000	-.0294232	.0347440
	[Condition=3]*[Day=1]	.0103890	.01210323	1	1.000	-.0283044	.0490824
	[Condition=3]*[Day=2]	.0473727[a]	.01299705	1	.010	.0058218	.0889236
[Condition=1]*[Day=2]	[Condition=1]*[Day=0]	-.0001469	.01090585	1	1.000	-.0350124	.0347185
	[Condition=1]*[Day=1]	.0041634	.01280245	1	1.000	-.0367654	.0450922
	[Condition=2]*[Day=0]	.0109742	.01095962	1	1.000	-.0240631	.0460116
	[Condition=2]*[Day=1]	-.0065270	.01310049	1	1.000	-.0484086	.0353546
	[Condition=2]*[Day=2]	.0378540	.01346201	1	.177	-.0051833	.0808914
	[Condition=3]*[Day=0]	.0068239	.01107108	1	1.000	-.0285698	.0422176
	[Condition=3]*[Day=1]	.0145525	.01297466	1	1.000	-.0269269	.0560318
	[Condition=3]*[Day=2]	.0515361[a]	.01381220	1	.007	.0073792	.0956931
[Condition=2]*[Day=0]	[Condition=1]*[Day=0]	-.0111212	.00730030	1	1.000	-.0344599	.0122175
	[Condition=1]*[Day=1]	-.0068108	.00991259	1	1.000	-.0385009	.0248793
	[Condition=1]*[Day=2]	-.0109742	.01095962	1	1.000	-.0460116	.0240631
	[Condition=2]*[Day=1]	-.0175013	.01029464	1	1.000	-.0504127	.0154102
	[Condition=2]*[Day=2]	.0268798	.01075093	1	.447	-.0074904	.0612500
	[Condition=3]*[Day=0]	-.0041504	.00754490	1	1.000	-.0282711	.0199703
	[Condition=3]*[Day=1]	.0035782	.01013403	1	1.000	-.0288198	.0359762

	[Condition=3]*[Day=2]	.0405619[a]	.01118631	1	.010	.0047998	.0763240
[Condition=2]*[Day=1]	[Condition=1]*[Day=0]	.0063801	.01023738	1	1.000	-.0263483	.0391085
	[Condition=1]*[Day=1]	.0106904	.01223802	1	1.000	-.0284339	.0498148
	[Condition=1]*[Day=2]	.0065270	.01310049	1	1.000	-.0353546	.0484086
	[Condition=2]*[Day=0]	.0175013	.01029464	1	1.000	-.0154102	.0504127
	[Condition=2]*[Day=2]	.0443811[a]	.01292641	1	.021	.0030560	.0857062
	[Condition=3]*[Day=0]	.0133509	.01041322	1	1.000	-.0199397	.0466414
	[Condition=3]*[Day=1]	.0210795	.01241806	1	1.000	-.0186204	.0607794
	[Condition=3]*[Day=2]	.0580632[a]	.01329072	1	.000	.0155734	.1005529
[Condition=2]*[Day=2]	[Condition=1]*[Day=0]	-.0380010[a]	.01069612	1	.014	-.0721959	-.0038060
	[Condition=1]*[Day=1]	-.0336906	.01262427	1	.274	-.0740498	.0066685
	[Condition=1]*[Day=2]	-.0378540	.01346201	1	.177	-.0808914	.0051833
	[Condition=2]*[Day=0]	-.0268798	.01075093	1	.447	-.0612500	.0074904
	[Condition=2]*[Day=1]	-.0443811[a]	.01292641	1	.021	-.0857062	-.0030560
	[Condition=3]*[Day=0]	-.0310302	.01086454	1	.154	-.0657636	.0037032
	[Condition=3]*[Day=1]	-.0233016	.01279887	1	1.000	-.0642189	.0176158
	[Condition=3]*[Day=2]	.0136821	.01364721	1	1.000	-.0299473	.0573115
[Condition=3]*[Day=0]	[Condition=1]*[Day=0]	-.0069708	.00746659	1	1.000	-.0308411	.0168995
	[Condition=1]*[Day=1]	-.0026604	.01003569	1	1.000	-.0347440	.0294232
	[Condition=1]*[Day=2]	-.0068239	.01107108	1	1.000	-.0422176	.0285698
	[Condition=2]*[Day=0]	.0041504	.00754490	1	1.000	-.0199703	.0282711
	[Condition=2]*[Day=1]	-.0133509	.01041322	1	1.000	-.0466414	.0199397
	[Condition=2]*[Day=2]	.0310302	.01086454	1	.154	-.0037032	.0657636
	[Condition=3]*[Day=1]	.0077286	.01025447	1	1.000	-.0250544	.0405116
	[Condition=3]*[Day=2]	.0447123[a]	.01129554	1	.003	.0086010	.0808236
[Condition=3]*[Day=1]	[Condition=1]*[Day=0]	-.0146994	.01007586	1	1.000	-.0469114	.0175126
	[Condition=1]*[Day=1]	-.0103890	.01210323	1	1.000	-.0490824	.0283044
	[Condition=1]*[Day=2]	-.0145525	.01297466	1	1.000	-.0560318	.0269269
	[Condition=2]*[Day=0]	-.0035782	.01013403	1	1.000	-.0359762	.0288198
	[Condition=2]*[Day=1]	-.0210795	.01241806	1	1.000	-.0607794	.0186204

	[Condition=2]*[Day=2]	.0233016	.01279887	1	1.000	-.0176158	.0642189
	[Condition=3]*[Day=0]	-.0077286	.01025447	1	1.000	-.0405116	.0250544
	[Condition=3]*[Day=2]	.0369837	.01316671	1	.179	-.0051096	.0790770
[Condition=3]*[Day=2]	[Condition=1]*[Day=0]	-.0516831[a]	.01113364	1	.000	-.0872768	-.0160894
	[Condition=1]*[Day=1]	-.0473727[a]	.01299705	1	.010	-.0889236	-.0058218
	[Condition=1]*[Day=2]	-.0515361[a]	.01381220	1	.007	-.0956931	-.0073792
	[Condition=2]*[Day=0]	-.0405619[a]	.01118631	1	.010	-.0763240	-.0047998
	[Condition=2]*[Day=1]	-.0580632[a]	.01329072	1	.000	-.1005529	-.0155734
	[Condition=2]*[Day=2]	-.0136821	.01364721	1	1.000	-.0573115	.0299473
	[Condition=3]*[Day=0]	-.0447123[a]	.01129554	1	.003	-.0808236	-.0086010
	[Condition=3]*[Day=1]	-.0369837	.01316671	1	.179	-.0790770	.0051096

Pairwise comparisons of estimated marginal means based on the original scale of dependent variable Baseline Diastolic SL

a. The mean difference is significant at the .05 level.

Overall Test Results

Wald Chi-Square	df	Sig.
36.010	8	.000

The Wald chi-square tests the effect of Condition*Day. This test is based on the linearly independent pairwise comparisons among the estimated marginal means.

Peak (Systolic) Sarcomere Length [μm]

```
MEANS TABLES=PeakSL BY Condition BY Day
  /CELLS=MEAN COUNT STDDEV MIN MAX.
```

Means

Case Processing Summary

	Cases					
	Included		Excluded		Total	
	N	Percent	N	Percent	N	Percent
Peak Systolic SL * Condition * Day	656	97.6%	16	2.4%	672	100.0%

Report

Peak Systolic SL

Condition	Day	Mean	N	Std. Deviation	Minimum	Maximum
1	0	1.6051260	139	.09221841	1.26868	1.78255
	1	1.6515212	51	.08133151	1.45225	1.84535
	2	1.6757275	39	.08307868	1.48835	1.84324
	Total	1.6274824	229	.09263029	1.26868	1.84535
2	0	1.5966888	133	.09304394	1.27085	1.83793
	1	1.7028677	46	.07249269	1.46086	1.88725
	2	1.6585053	41	.09214832	1.32686	1.79348
	Total	1.6304102	220	.09894393	1.27085	1.88725
3	0	1.6323878	122	.08758130	1.35300	1.82673

	1	1.6953794	48	.08440485	1.44271	1.79666
	2	1.6770424	37	.09332321	1.44500	1.83590
	Total	1.6549763	207	.09177637	1.35300	1.83590
Total	0	1.6107194	394	.09208184	1.26868	1.83793
	1	1.6823290	145	.08241349	1.44271	1.88725
	2	1.6701082	117	.08924943	1.32686	1.84324
	Total	1.6371399	656	.09517045	1.26868	1.88725

```
* Generalized Linear Models.
GENLIN PeakSL BY Condition Day (ORDER=ASCENDING)
  /MODEL Condition Day Condition*Day INTERCEPT=YES
 DISTRIBUTION=NORMAL LINK=IDENTITY
  /CRITERIA SCALE=MLE COVB=MODEL PCONVERGE=1E-006(ABSOLUTE) SINGULAR=1E-012 ANALYSISTYPE=3(WALD)
    CILEVEL=95 CITYPE=WALD LIKELIHOOD=FULL
  /EMMEANS TABLES=Condition*Day SCALE=ORIGINAL COMPARE=Condition*Day CONTRAST=PAIRWISE
    PADJUST=BONFERRONI
  /MISSING CLASSMISSING=EXCLUDE
  /PRINT CPS DESCRIPTIVES MODELINFO FIT SUMMARY SOLUTION.
```

Generalized Linear Models

Model Information

Dependent Variable	Peak Systolic SL
Probability Distribution	Normal
Link Function	Identity

Case Processing Summary

	N	Percent
Included	656	97.6%
Excluded	16	2.4%
Total	672	100.0%

Categorical Variable Information

			N	Percent
Factor	Condition	1	229	34.9%
		2	220	33.5%
		3	207	31.6%
		Total	656	100.0%
	Day	0	394	60.1%
		1	145	22.1%
		2	117	17.8%
		Total	656	100.0%

Continuous Variable Information

		N	Minimum	Maximum	Mean	Std. Deviation
Dependent Variable	Peak Systolic SL	656	1.26868	1.88725	1.6371399	.09517045

Goodness of Fit[a]

	Value	df	Value/df
Deviance	5.062	647	.008
Scaled Deviance	656.000	647	
Pearson Chi-Square	5.062	647	.008
Scaled Pearson Chi-Square	656.000	647	
Log Likelihood[b]	664.700		

Akaike's Information Criterion (AIC)	-1309.401		
Finite Sample Corrected AIC (AICC)	-1309.059		
Bayesian Information Criterion (BIC)	-1264.539		
Consistent AIC (CAIC)	-1254.539		

Dependent Variable: Peak Systolic SL

Model: (Intercept), Condition, Day, Condition * Day

a. Information criteria are in smaller-is-better form.

b. The full log likelihood function is displayed and used in computing information criteria.

Omnibus Test[a]

Likelihood Ratio Chi-Square	df	Sig.
104.111	8	.000

Dependent Variable: Peak Systolic SL

Model: (Intercept), Condition, Day, Condition * Day

a. Compares the fitted model against the intercept-only model.

Tests of Model Effects

Source	Type III		
	Wald Chi-Square	df	Sig.
(Intercept)	177323.322	1	.000
Condition	6.406	2	.041
Day	90.348	2	.000

Condition * Day	10.827	4	.029

Dependent Variable: Peak Systolic SL

Model: (Intercept), Condition, Day, Condition * Day

Parameter Estimates

Parameter	B	Std. Error	95% Wald Confidence Interval Lower	95% Wald Confidence Interval Upper	Hypothesis Test Wald Chi-Square	Hypothesis Test df	Hypothesis Test Sig.
(Intercept)	1.677	.0144	1.649	1.705	13485.680	1	.000
[Condition=1]	-.001	.0202	-.041	.038	.004	1	.948
[Condition=2]	-.019	.0199	-.058	.021	.866	1	.352
[Condition=3]	0[a]	.	.	.	.	.	.
[Day=0]	-.045	.0165	-.077	-.012	7.336	1	.007
[Day=1]	.018	.0192	-.019	.056	.910	1	.340
[Day=2]	0[a]	.	.	.	.	.	.
[Condition=1] * [Day=0]	-.026	.0229	-.071	.019	1.282	1	.258
[Condition=1] * [Day=1]	-.043	.0268	-.095	.010	2.519	1	.112
[Condition=1] * [Day=2]	0[a]	.	.	.	.	.	.
[Condition=2] * [Day=0]	-.017	.0228	-.062	.027	.569	1	.451
[Condition=2] * [Day=1]	.026	.0269	-.027	.079	.934	1	.334
[Condition=2] * [Day=2]	0[a]	.	.	.	.	.	.
[Condition=3] * [Day=0]	0[a]	.	.	.	.	.	.
[Condition=3] * [Day=1]	0[a]	.	.	.	.	.	.
[Condition=3] * [Day=2]	0[a]	.	.	.	.	.	.
(Scale)	.008[b]	.0004	.007	.009			

Dependent Variable: Peak Systolic SL

Model: (Intercept), Condition, Day, Condition * Day

a. Set to zero because this parameter is redundant.

b. Maximum likelihood estimate.

Estimated Marginal Means: Condition* Day

Estimates

Condition	Day	Mean	Std. Error	95% Wald Confidence Interval	
				Lower	Upper
1	0	1.6051260	.00745077	1.5905227	1.6197292
	1	1.6515212	.01230052	1.6274126	1.6756297
	2	1.6757275	.01406618	1.6481583	1.7032967
2	0	1.5966888	.00761698	1.5817598	1.6116178
	1	1.7028677	.01295178	1.6774827	1.7282528
	2	1.6585053	.01371881	1.6316169	1.6853936
3	0	1.6323878	.00795296	1.6168003	1.6479753
	1	1.6953794	.01267908	1.6705289	1.7202300
	2	1.6770424	.01444134	1.6487379	1.7053469

Pairwise Comparisons

(I) Condition*Day	(J) Condition*Day	Mean Difference (I-J)	Std. Error	df	Bonferroni Sig.	95% Wald Confidence Interval for Difference	
						Lower	Upper
[Condition=1]*[Day=0]	[Condition=1]*[Day=1]	-.0463952[a]	.01438112	1	.045	-.0923709	-.0004194
	[Condition=1]*[Day=2]	-.0706015[a]	.01591764	1	.000	-.1214895	-.0197136
	[Condition=2]*[Day=0]	.0084371	.01065515	1	1.000	-.0256268	.0425011
	[Condition=2]*[Day=1]	-.0977417[a]	.01494198	1	.000	-.1455105	-.0499730
	[Condition=2]*[Day=2]	-.0533793[a]	.01561153	1	.023	-.1032886	-.0034700
	[Condition=3]*[Day=0]	-.0272618	.01089786	1	.445	-.0621017	.0075781

	[Condition=3]*[Day=1]	-.0902534[a]	.01470623	1	.000	-.1372685	-.0432384
	[Condition=3]*[Day=2]	-.0719164[a]	.01625012	1	.000	-.1238672	-.0199656
[Condition=1]*[Day=1]	[Condition=1]*[Day=0]	.0463952[a]	.01438112	1	.045	.0004194	.0923709
	[Condition=1]*[Day=2]	-.0242064	.01868583	1	1.000	-.0839440	.0355313
	[Condition=2]*[Day=0]	.0548323[a]	.01446793	1	.005	.0085791	.1010856
	[Condition=2]*[Day=1]	-.0513466	.01786201	1	.146	-.1084505	.0057574
	[Condition=2]*[Day=2]	-.0069841	.01842576	1	1.000	-.0658903	.0519221
	[Condition=3]*[Day=0]	.0191334	.01464760	1	1.000	-.0276943	.0659610
	[Condition=3]*[Day=1]	-.0438583	.01766527	1	.469	-.1003332	.0126167
	[Condition=3]*[Day=2]	-.0255212	.01896985	1	1.000	-.0861669	.0351245
[Condition=1]*[Day=2]	[Condition=1]*[Day=0]	.0706015[a]	.01591764	1	.000	.0197136	.1214895
	[Condition=1]*[Day=1]	.0242064	.01868583	1	1.000	-.0355313	.0839440
	[Condition=2]*[Day=0]	.0790387[a]	.01599612	1	.000	.0278999	.1301775
	[Condition=2]*[Day=1]	-.0271402	.01912083	1	1.000	-.0882685	.0339881
	[Condition=2]*[Day=2]	.0172223	.01964849	1	1.000	-.0455930	.0800375
	[Condition=3]*[Day=0]	.0433398	.01615880	1	.263	-.0083191	.0949986
	[Condition=3]*[Day=1]	-.0196519	.01893717	1	1.000	-.0801931	.0408893
	[Condition=3]*[Day=2]	-.0013148	.02015961	1	1.000	-.0657641	.0631344
[Condition=2]*[Day=0]	[Condition=1]*[Day=0]	-.0084371	.01065515	1	1.000	-.0425011	.0256268
	[Condition=1]*[Day=1]	-.0548323[a]	.01446793	1	.005	-.1010856	-.0085791
	[Condition=1]*[Day=2]	-.0790387[a]	.01599612	1	.000	-.1301775	-.0278999
	[Condition=2]*[Day=1]	-.1061789[a]	.01502555	1	.000	-.1542148	-.0581430
	[Condition=2]*[Day=2]	-.0618164[a]	.01569153	1	.003	-.1119815	-.0116514
	[Condition=3]*[Day=0]	-.0356989[a]	.01101217	1	.043	-.0709043	-.0004936
	[Condition=3]*[Day=1]	-.0986906[a]	.01479113	1	.000	-.1459771	-.0514041
	[Condition=3]*[Day=2]	-.0803535[a]	.01632699	1	.000	-.1325501	-.0281569
[Condition=2]*[Day=1]	[Condition=1]*[Day=0]	.0977417[a]	.01494198	1	.000	.0499730	.1455105
	[Condition=1]*[Day=1]	.0513466	.01786201	1	.146	-.0057574	.1084505
	[Condition=1]*[Day=2]	.0271402	.01912083	1	1.000	-.0339881	.0882685
	[Condition=2]*[Day=0]	.1061789[a]	.01502555	1	.000	.0581430	.1542148

	[Condition=2]*[Day=2]	.0443625	.01886676	1	.673	-.0159536	.1046785
	[Condition=3]*[Day=0]	.0704800[a]	.01519862	1	.000	.0218907	.1190692
	[Condition=3]*[Day=1]	.0074883	.01812478	1	1.000	-.0504557	.0654324
	[Condition=3]*[Day=2]	.0258254	.01939848	1	1.000	-.0361906	.0878413
[Condition=2]*[Day=2]	[Condition=1]*[Day=0]	.0533793[a]	.01561153	1	.023	.0034700	.1032886
	[Condition=1]*[Day=1]	.0069841	.01842576	1	1.000	-.0519221	.0658903
	[Condition=1]*[Day=2]	-.0172223	.01964849	1	1.000	-.0800375	.0455930
	[Condition=2]*[Day=0]	.0618164[a]	.01569153	1	.003	.0116514	.1119815
	[Condition=2]*[Day=1]	-.0443625	.01886676	1	.673	-.1046785	.0159536
	[Condition=3]*[Day=0]	.0261175	.01585734	1	1.000	-.0245776	.0768126
	[Condition=3]*[Day=1]	-.0368741	.01868060	1	1.000	-.0965951	.0228468
	[Condition=3]*[Day=2]	-.0185371	.01991879	1	1.000	-.0822165	.0451423
[Condition=3]*[Day=0]	[Condition=1]*[Day=0]	.0272618	.01089786	1	.445	-.0075781	.0621017
	[Condition=1]*[Day=1]	-.0191334	.01464760	1	1.000	-.0659610	.0276943
	[Condition=1]*[Day=2]	-.0433398	.01615880	1	.263	-.0949986	.0083191
	[Condition=2]*[Day=0]	.0356989[a]	.01101217	1	.043	.0004936	.0709043
	[Condition=2]*[Day=1]	-.0704800[a]	.01519862	1	.000	-.1190692	-.0218907
	[Condition=2]*[Day=2]	-.0261175	.01585734	1	1.000	-.0768126	.0245776
	[Condition=3]*[Day=1]	-.0629916[a]	.01496692	1	.001	-.1108401	-.0151431
	[Condition=3]*[Day=2]	-.0446546	.01648642	1	.243	-.0973608	.0080517
[Condition=3]*[Day=1]	[Condition=1]*[Day=0]	.0902534[a]	.01470623	1	.000	.0432384	.1372685
	[Condition=1]*[Day=1]	.0438583	.01766527	1	.469	-.0126167	.1003332
	[Condition=1]*[Day=2]	.0196519	.01893717	1	1.000	-.0408893	.0801931
	[Condition=2]*[Day=0]	.0986906[a]	.01479113	1	.000	.0514041	.1459771
	[Condition=2]*[Day=1]	-.0074883	.01812478	1	1.000	-.0654324	.0504557
	[Condition=2]*[Day=2]	.0368741	.01868060	1	1.000	-.0228468	.0965951
	[Condition=3]*[Day=0]	.0629916[a]	.01496692	1	.001	.0151431	.1108401
	[Condition=3]*[Day=2]	.0183370	.01921748	1	1.000	-.0431003	.0797744
[Condition=3]*[Day=2]	[Condition=1]*[Day=0]	.0719164[a]	.01625012	1	.000	.0199656	.1238672
	[Condition=1]*[Day=1]	.0255212	.01896985	1	1.000	-.0351245	.0861669

	[Condition=1]*[Day=2]	.0013148	.02015961	1	1.000	-.0631344	.0657641
	[Condition=2]*[Day=0]	.0803535[a]	.01632699	1	.000	.0281569	.1325501
	[Condition=2]*[Day=1]	-.0258254	.01939848	1	1.000	-.0878413	.0361906
	[Condition=2]*[Day=2]	.0185371	.01991879	1	1.000	-.0451423	.0822165
	[Condition=3]*[Day=0]	.0446546	.01648642	1	.243	-.0080517	.0973608
	[Condition=3]*[Day=1]	-.0183370	.01921748	1	1.000	-.0797744	.0431003

Pairwise comparisons of estimated marginal means based on the original scale of dependent variable Peak Systolic SL

a. The mean difference is significant at the .05 level.

Overall Test Results

Wald Chi-Square	df	Sig.
112.827	8	.000

The Wald chi-square tests the effect of Condition*Day. This test is based on the linearly independent pairwise comparisons among the estimated marginal means.

Sarcomere Length Shortening Magnitude [μm]

```
MEANS TABLES=SLmag BY Condition BY Day
  /CELLS=MEAN COUNT STDDEV MIN MAX.
```

Means

Case Processing Summary

	Cases					
	Included		Excluded		Total	
	N	Percent	N	Percent	N	Percent
Shortening Magnitude * Condition * Day	656	97.6%	16	2.4%	672	100.0%

Report

Shortening Magnitude

Condition	Day	Mean	N	Std. Deviation	Minimum	Maximum
1	0	.1631256	139	.06990247	.03368	.33141
	1	.1124312	51	.07636196	.00194	.28824
	2	.0923900	39	.06354084	.00982	.28492
	Total	.1397889	229	.07611127	.00194	.33141
2	0	.1604578	133	.06026090	.03825	.30256
	1	.0718257	46	.05731838	.00350	.25157
	2	.0717379	41	.06130112	.00747	.23854
	Total	.1253915	220	.07374692	.00350	.30256
3	0	.1288675	122	.06760672	.00500	.30850

	1	.0582160	48	.06515454	.00535	.27416
	2	.0395389	37	.03489490	.00100	.15343
	Total	.0965176	207	.07355803	.00100	.30850
Total	0	.1516172	394	.06765154	.00500	.33141
	1	.0816023	145	.07061443	.00194	.28824
	2	.0684393	117	.05881685	.00100	.28492
	Total	.1213063	656	.07652332	.00100	.33141

```
* Generalized Linear Models.
GENLIN SLmag BY Condition Day (ORDER=ASCENDING)
  /MODEL Condition Day Condition*Day INTERCEPT=YES
 DISTRIBUTION=NORMAL LINK=IDENTITY
  /CRITERIA SCALE=MLE COVB=MODEL PCONVERGE=1E-006(ABSOLUTE) SINGULAR=1E-012 ANALYSISTYPE=3(WALD)
    CILEVEL=95 CITYPE=WALD LIKELIHOOD=FULL
  /EMMEANS TABLES=Condition SCALE=ORIGINAL COMPARE=Condition CONTRAST=PAIRWISE PADJUST=BONFERRONI
  /EMMEANS TABLES=Day SCALE=ORIGINAL COMPARE=Day CONTRAST=PAIRWISE PADJUST=BONFERRONI
  /MISSING CLASSMISSING=EXCLUDE
  /PRINT CPS DESCRIPTIVES MODELINFO FIT SUMMARY SOLUTION.
```

Generalized Linear Models

Model Information

Dependent Variable	Shortening Magnitude
Probability Distribution	Normal
Link Function	Identity

Case Processing Summary

	N	Percent
Included	656	97.6%
Excluded	16	2.4%
Total	672	100.0%

Categorical Variable Information

			N	Percent
Factor	Condition	1	229	34.9%
		2	220	33.5%
		3	207	31.6%
		Total	656	100.0%
	Day	0	394	60.1%
		1	145	22.1%
		2	117	17.8%
		Total	656	100.0%

Continuous Variable Information

		N	Minimum	Maximum	Mean	Std. Deviation
Dependent Variable	Shortening Magnitude	656	.00100	.33141	.1213063	.07652332

Goodness of Fit[a]

	Value	df	Value/df
Deviance	2.693	647	.004
Scaled Deviance	656.000	647	
Pearson Chi-Square	2.693	647	.004
Scaled Pearson Chi-Square	656.000	647	
Log Likelihood[b]	871.677		

Akaike's Information Criterion (AIC)	-1723.355		
Finite Sample Corrected AIC (AICC)	-1723.014		
Bayesian Information Criterion (BIC)	-1678.493		
Consistent AIC (CAIC)	-1668.493		

Dependent Variable: Shortening Magnitude

Model: (Intercept), Condition, Day, Condition * Day

a. Information criteria are in smaller-is-better form.

b. The full log likelihood function is displayed and used in computing information criteria.

Omnibus Test[a]

Likelihood Ratio Chi-Square	df	Sig.
231.952	8	.000

Dependent Variable: Shortening Magnitude

Model: (Intercept), Condition, Day, Condition * Day

a. Compares the fitted model against the intercept-only model.

Tests of Model Effects

	Type III		
Source	Wald Chi-Square	df	Sig.
(Intercept)	1212.959	1	.000
Condition	44.895	2	.000
Day	222.189	2	.000

Condition * Day	6.953	4	.138

Dependent Variable: Shortening Magnitude

Model: (Intercept), Condition, Day, Condition * Day

Parameter Estimates

			95% Wald Confidence Interval		Hypothesis Test		
Parameter	B	Std. Error	Lower	Upper	Wald Chi-Square	df	Sig.
(Intercept)	.040	.0105	.019	.060	14.089	1	.000
[Condition=1]	.053	.0147	.024	.082	12.918	1	.000
[Condition=2]	.032	.0145	.004	.061	4.911	1	.027
[Condition=3]	0[a]	.	.	.	.	.	.
[Day=0]	.089	.0120	.066	.113	55.180	1	.000
[Day=1]	.019	.0140	-.009	.046	1.775	1	.183
[Day=2]	0[a]	.	.	.	.	.	.
[Condition=1] * [Day=0]	-.019	.0167	-.051	.014	1.237	1	.266
[Condition=1] * [Day=1]	.001	.0196	-.037	.040	.005	1	.944
[Condition=1] * [Day=2]	0[a]	.	.	.	.	.	.
[Condition=2] * [Day=0]	-.001	.0166	-.033	.032	.001	1	.971
[Condition=2] * [Day=1]	-.019	.0196	-.057	.020	.896	1	.344
[Condition=2] * [Day=2]	0[a]	.	.	.	.	.	.
[Condition=3] * [Day=0]	0[a]	.	.	.	.	.	.
[Condition=3] * [Day=1]	0[a]	.	.	.	.	.	.
[Condition=3] * [Day=2]	0[a]	.	.	.	.	.	.
(Scale)	.004[b]	.0002	.004	.005			

Dependent Variable: Shortening Magnitude

Model: (Intercept), Condition, Day, Condition * Day

a. Set to zero because this parameter is redundant.

b. Maximum likelihood estimate.

Estimated Marginal Means 1: Condition

Estimates

Condition	Mean	Std. Error	95% Wald Confidence Interval	
			Lower	Upper
1	.1226489	.00489109	.1130626	.1322353
2	.1013405	.00494696	.0916446	.1110363
3	.0755408	.00505681	.0656296	.0854519

Pairwise Comparisons

(I) Condition	(J) Condition	Mean Difference (I-J)	Std. Error	df	Bonferroni Sig.	95% Wald Confidence Interval for Difference	
						Lower	Upper
1	2	.0213085[a]	.00695667	1	.007	.0046544	.0379626
	3	.0471082[a]	.00703520	1	.000	.0302661	.0639503
2	1	-.0213085[a]	.00695667	1	.007	-.0379626	-.0046544
	3	.0257997[a]	.00707416	1	.001	.0088643	.0427351
3	1	-.0471082[a]	.00703520	1	.000	-.0639503	-.0302661
	2	-.0257997[a]	.00707416	1	.001	-.0427351	-.0088643

Pairwise comparisons of estimated marginal means based on the original scale of dependent variable Shortening Magnitude

a. The mean difference is significant at the .05 level.

Overall Test Results

Wald Chi-Square	df	Sig.

44.895	2	.000

The Wald chi-square tests the effect of Condition. This test is based on the linearly independent pairwise comparisons among the estimated marginal means.

Estimated Marginal Means 2: Day

Estimates

Day	Mean	Std. Error	95% Wald Confidence Interval	
			Lower	Upper
0	.1508170	.00323275	.1444809	.1571530
1	.0808243	.00532583	.0703858	.0912627
2	.0678889	.00592886	.0562686	.0795093

Pairwise Comparisons

(I) Day	(J) Day	Mean Difference (I-J)	Std. Error	df	Bonferroni Sig.	95% Wald Confidence Interval for Difference	
						Lower	Upper
0	1	.0699927[a]	.00623018	1	.000	.0550777	.0849076
	2	.0829280[a]	.00675292	1	.000	.0667617	.0990944
1	0	-.0699927[a]	.00623018	1	.000	-.0849076	-.0550777
	2	.0129354	.00796968	1	.314	-.0061439	.0320146
2	0	-.0829280[a]	.00675292	1	.000	-.0990944	-.0667617
	1	-.0129354	.00796968	1	.314	-.0320146	.0061439

Pairwise comparisons of estimated marginal means based on the original scale of dependent variable Shortening Magnitude

a. The mean difference is significant at the .05 level.

Overall Test Results

Wald Chi-Square	df	Sig.
222.189	2	.000

The Wald chi-square tests the effect of Day. This test is based on the linearly independent pairwise comparisons among the estimated marginal means.

Time to Peak Sarcomere Length [ms]

```
MEANS TABLES=TTP BY Condition BY Day
  /CELLS=MEAN COUNT STDDEV MIN MAX.
```

Means

Case Processing Summary

	Cases					
	Included		Excluded		Total	
	N	Percent	N	Percent	N	Percent
TTP * Condition * Day	654	97.3%	18	2.7%	672	100.0%

Report

TTP

Condition	Day	Mean	N	Std. Deviation	Minimum	Maximum
1	0	245.2176837	139	45.42184992	145.43750	368.66667
	1	281.7457760	51	86.59060816	123.20000	502.00000
	2	223.9756547	39	57.91543995	149.12500	361.55000
	Total	249.7351229	229	61.70259670	123.20000	502.00000
2	0	253.5880960	133	60.81826524	120.31818	395.60000
	1	290.9095352	46	73.06933796	162.53333	479.96970
	2	308.7098940	41	74.86450670	140.45455	532.40000
	Total	271.6643684	220	69.88120175	120.31818	532.40000
3	0	268.3477795	122	58.17775281	151.75000	488.45455
	1	312.4651175	48	90.34637711	165.11429	536.82857
	2	326.6854067	35	107.51817890	156.66667	597.36842
	Total	288.6377755	205	80.26817782	151.75000	597.36842

Total	0	255.2053399	394	55.66604020	120.31818	488.45455
	1	294.8220609	145	84.29278326	123.20000	536.82857
	2	285.4447428	115	92.42217673	140.45455	597.36842
	Total	269.3061898	654	72.34234409	120.31818	597.36842

```
* Generalized Linear Models.
GENLIN TTP BY Condition Day (ORDER=ASCENDING)
  /MODEL Condition Day Condition*Day INTERCEPT=YES
 DISTRIBUTION=NORMAL LINK=IDENTITY
  /CRITERIA SCALE=MLE COVB=MODEL PCONVERGE=1E-006(ABSOLUTE) SINGULAR=1E-012 ANALYSISTYPE=3(WALD)
    CILEVEL=95 CITYPE=WALD LIKELIHOOD=FULL
  /EMMEANS TABLES=Condition SCALE=ORIGINAL COMPARE=Condition CONTRAST=PAIRWISE PADJUST=BONFERRONI
  /EMMEANS TABLES=Day SCALE=ORIGINAL COMPARE=Day CONTRAST=PAIRWISE PADJUST=BONFERRONI
  /EMMEANS TABLES=Condition*Day SCALE=ORIGINAL COMPARE=Condition*Day CONTRAST=PAIRWISE
    PADJUST=BONFERRONI
  /MISSING CLASSMISSING=EXCLUDE
  /PRINT CPS DESCRIPTIVES MODELINFO FIT SUMMARY SOLUTION.
```

Generalized Linear Models

Model Information

Dependent Variable	TTP
Probability Distribution	Normal
Link Function	Identity

Case Processing Summary

	N	Percent
Included	654	97.3%
Excluded	18	2.7%

Total	672	100.0%

Categorical Variable Information

			N	Percent
Factor	Condition	1	229	35.0%
		2	220	33.6%
		3	205	31.3%
		Total	654	100.0%
	Day	0	394	60.2%
		1	145	22.2%
		2	115	17.6%
		Total	654	100.0%

Continuous Variable Information

		N	Minimum	Maximum	Mean	Std. Deviation
Dependent Variable	TTP	654	120.31818	597.36842	269.3061898	72.34234409

Goodness of Fit[a]

	Value	df	Value/df
Deviance	2925992.530	645	4536.423
Scaled Deviance	654.000	645	
Pearson Chi-Square	2925992.530	645	4536.423
Scaled Pearson Chi-Square	654.000	645	
Log Likelihood[b]	-3676.760		
Akaike's Information Criterion (AIC)	7373.520		

Finite Sample Corrected AIC (AICC)	7373.862		
Bayesian Information Criterion (BIC)	7418.351		
Consistent AIC (CAIC)	7428.351		

Dependent Variable: TTP

Model: (Intercept), Condition, Day, Condition * Day

a. Information criteria are in smaller-is-better form.

b. The full log likelihood function is displayed and used in computing information criteria.

Omnibus Test[a]

Likelihood Ratio Chi-Square	df	Sig.
101.535	8	.000

Dependent Variable: TTP

Model: (Intercept), Condition, Day, Condition * Day

a. Compares the fitted model against the intercept-only model.

Tests of Model Effects

	Type III		
Source	Wald Chi-Square	df	Sig.
(Intercept)	8613.736	1	.000
Condition	51.893	2	.000
Day	45.123	2	.000
Condition * Day	28.335	4	.000

Dependent Variable: TTP

Model: (Intercept), Condition, Day, Condition * Day

Parameter Estimates

Parameter	B	Std. Error	95% Wald Confidence Interval		Hypothesis Test		
			Lower	Upper	Wald Chi-Square	df	Sig.
(Intercept)	326.685	11.3061	304.526	348.845	834.895	1	.000
[Condition=1]	-102.710	15.5739	-133.234	-72.185	43.494	1	.000
[Condition=2]	-17.976	15.3932	-48.146	12.195	1.364	1	.243
[Condition=3]	0[a]	.	.	.	.	.	.
[Day=0]	-58.338	12.8258	-83.476	-33.200	20.689	1	.000
[Day=1]	-14.220	14.8673	-43.360	14.919	.915	1	.339
[Day=2]	0[a]	.	.	.	.	.	.
[Condition=1] * [Day=0]	79.580	17.6467	44.993	114.167	20.337	1	.000
[Condition=1] * [Day=1]	71.990	20.5786	31.657	112.324	12.238	1	.000
[Condition=1] * [Day=2]	0[a]	.	.	.	.	.	.
[Condition=2] * [Day=0]	3.216	17.5289	-31.140	37.572	.034	1	.854
[Condition=2] * [Day=1]	-3.580	20.6741	-44.101	36.940	.030	1	.863
[Condition=2] * [Day=2]	0[a]	.	.	.	.	.	.
[Condition=3] * [Day=0]	0[a]	.	.	.	.	.	.
[Condition=3] * [Day=1]	0[a]	.	.	.	.	.	.
[Condition=3] * [Day=2]	0[a]	.	.	.	.	.	.
(Scale)	4473.995[b]	247.4125	4014.430	4986.169			

Dependent Variable: TTP

Model: (Intercept), Condition, Day, Condition * Day

a. Set to zero because this parameter is redundant.

b. Maximum likelihood estimate.

Estimated Marginal Means 1: Condition

Estimates

Condition	Mean	Std. Error	95% Wald Confidence Interval	
			Lower	Upper
1	250.3130381	5.10588126	240.3056947	260.3203815
2	284.4025084	5.16421053	274.2808417	294.5241751
3	302.4994346	5.35110300	292.0114654	312.9874037

Pairwise Comparisons

(I) Condition	(J) Condition	Mean Difference (I-J)	Std. Error	df	Bonferroni Sig.	95% Wald Confidence Interval for Difference	
						Lower	Upper
1	2	-34.0894703[a]	7.26216868	1	.000	-51.4749554	-16.7039852
	3	-52.1863965[a]	7.39623734	1	.000	-69.8928392	-34.4799537
2	1	34.0894703[a]	7.26216868	1	.000	16.7039852	51.4749554
	3	-18.0969262[a]	7.43662382	1	.045	-35.9000534	-.2937990
3	1	52.1863965[a]	7.39623734	1	.000	34.4799537	69.8928392
	2	18.0969262[a]	7.43662382	1	.045	.2937990	35.9000534

Pairwise comparisons of estimated marginal means based on the original scale of dependent variable TTP

a. The mean difference is significant at the .05 level.

Overall Test Results

Wald Chi-Square	df	Sig.
51.893	2	.000

The Wald chi-square tests the effect of Condition. This test is based on the linearly independent pairwise comparisons among the estimated marginal means.

Estimated Marginal Means 2: Day

Estimates

Day	Mean	Std. Error	95% Wald Confidence Interval Lower	95% Wald Confidence Interval Upper
0	255.7178531	3.37471246	249.1035382	262.3321679
1	295.0401429	5.55971985	284.1432922	305.9369936
2	286.4569851	6.25093819	274.2053714	298.7085989

Pairwise Comparisons

(I) Day	(J) Day	Mean Difference (I-J)	Std. Error	df	Bonferroni Sig.	95% Wald Confidence Interval for Difference Lower	95% Wald Confidence Interval for Difference Upper
0	1	-39.3222898[a]	6.50378113	1	.000	-54.8922105	-23.7523692
	2	-30.7391321[a]	7.10372525	1	.000	-47.7453068	-13.7329573
1	0	39.3222898[a]	6.50378113	1	.000	23.7523692	54.8922105
	2	8.5831577	8.36568665	1	.915	-11.4441271	28.6104426
2	0	30.7391321[a]	7.10372525	1	.000	13.7329573	47.7453068
	1	-8.5831577	8.36568665	1	.915	-28.6104426	11.4441271

Pairwise comparisons of estimated marginal means based on the original scale of dependent variable TTP

a. The mean difference is significant at the .05 level.

Overall Test Results

Wald Chi-Square	df	Sig.
45.123	2	.000

The Wald chi-square tests the effect of Day. This test is based on the linearly independent pairwise comparisons among the estimated marginal means.

Estimated Marginal Means 3: Condition* Day

Estimates

				95% Wald Confidence Interval	
Condition	Day	Mean	Std. Error	Lower	Upper
1	0	245.2176837	5.67335987	234.0981027	256.3372647
	1	281.7457760	9.36618312	263.3883944	300.1031575
	2	223.9756547	10.71064016	202.9831857	244.9681237
2	0	253.5880960	5.79991878	242.2204641	264.9557279
	1	290.9095352	9.86208671	271.5802005	310.2388700
	2	308.7098940	10.44613909	288.2358376	329.1839503
3	0	268.3477795	6.05574832	256.4787309	280.2168281
	1	312.4651175	9.65444057	293.5427616	331.3874733
	2	326.6854067	11.30612311	304.5258126	348.8450008

Pairwise Comparisons

(I) Condition*Day	(J) Condition*Day	Mean Difference (I-J)	Std. Error	df	Bonferroni Sig.	95% Wald Confidence Interval for Difference Lower	Upper
[Condition=1]*[Day=0]	[Condition=1]*[Day=1]	-36.5280923[a]	10.95045197	1	.031	-71.5361422	-1.5200423
	[Condition=1]*[Day=2]	21.2420290	12.12043006	1	1.000	-17.5063827	59.9904406
	[Condition=2]*[Day=0]	-8.3704123	8.11332669	1	1.000	-34.3083140	17.5674893
	[Condition=2]*[Day=1]	-45.6918515[a]	11.37751143	1	.002	-82.0651893	-9.3185138
	[Condition=2]*[Day=2]	-63.4922103[a]	11.88733923	1	.000	-101.4954422	-25.4889784
	[Condition=3]*[Day=0]	-23.1300958	8.29813834	1	.191	-49.6588311	3.3986394
	[Condition=3]*[Day=1]	-67.2474338[a]	11.19800138	1	.000	-103.0468869	-31.4479807
	[Condition=3]*[Day=2]	-81.4677231[a]	12.64972063	1	.000	-121.9082503	-41.0271958
[Condition=1]*[Day=1]	[Condition=1]*[Day=0]	36.5280923[a]	10.95045197	1	.031	1.5200423	71.5361422
	[Condition=1]*[Day=2]	57.7701213[a]	14.22825354	1	.002	12.2831028	103.2571397
	[Condition=2]*[Day=0]	28.1576799	11.01655318	1	.381	-7.0616923	63.3770522
	[Condition=2]*[Day=1]	-9.1637593	13.60096101	1	1.000	-52.6453547	34.3178361
	[Condition=2]*[Day=2]	-26.9641180	14.03022480	1	1.000	-71.8180484	17.8898124
	[Condition=3]*[Day=0]	13.3979965	11.15336155	1	1.000	-22.2587453	49.0547382
	[Condition=3]*[Day=1]	-30.7193415	13.45115642	1	.806	-73.7220191	12.2833361
	[Condition=3]*[Day=2]	-44.9396308	14.68175078	1	.079	-91.8764573	1.9971957
[Condition=1]*[Day=2]	[Condition=1]*[Day=0]	-21.2420290	12.12043006	1	1.000	-59.9904406	17.5063827
	[Condition=1]*[Day=1]	-57.7701213[a]	14.22825354	1	.002	-103.2571397	-12.2831028
	[Condition=2]*[Day=0]	-29.6124413	12.18018352	1	.542	-68.5518818	9.3269992
	[Condition=2]*[Day=1]	-66.9338805[a]	14.55948375	1	.000	-113.4798254	-20.3879356
	[Condition=2]*[Day=2]	-84.7342393[a]	14.96127115	1	.000	-132.5646785	-36.9038000
	[Condition=3]*[Day=0]	-44.3721248[a]	12.30406032	1	.011	-83.7075933	-5.0366563
	[Condition=3]*[Day=1]	-88.4894628[a]	14.41964062	1	.000	-134.5883361	-42.3905894
	[Condition=3]*[Day=2]	-102.7097520[a]	15.57389587	1	.000	-152.4987220	-52.9207821
[Condition=2]*[Day=0]	[Condition=1]*[Day=0]	8.3704123	8.11332669	1	1.000	-17.5674893	34.3083140

	[Condition=1]*[Day=1]	-28.1576799	11.01655318	1	.381	-63.3770522	7.0616923
	[Condition=1]*[Day=2]	29.6124413	12.18018352	1	.542	-9.3269992	68.5518818
	[Condition=2]*[Day=1]	-37.3214392[a]	11.44114558	1	.040	-73.8982122	-.7446663
	[Condition=2]*[Day=2]	-55.1217979[a]	11.94825844	1	.000	-93.3197855	-16.9238104
	[Condition=3]*[Day=0]	-14.7596835	8.38517415	1	1.000	-41.5666679	12.0473009
	[Condition=3]*[Day=1]	-58.8770214[a]	11.26264981	1	.000	-94.8831523	-22.8708906
	[Condition=3]*[Day=2]	-73.0973107[a]	12.70698539	1	.000	-113.7209106	-32.4737109
[Condition=2]*[Day=1]	[Condition=1]*[Day=0]	45.6918515[a]	11.37751143	1	.002	9.3185138	82.0651893
	[Condition=1]*[Day=1]	9.1637593	13.60096101	1	1.000	-34.3178361	52.6453547
	[Condition=1]*[Day=2]	66.9338805[a]	14.55948375	1	.000	20.3879356	113.4798254
	[Condition=2]*[Day=0]	37.3214392[a]	11.44114558	1	.040	.7446663	73.8982122
	[Condition=2]*[Day=2]	-17.8003587	14.36602158	1	1.000	-63.7278147	28.1270973
	[Condition=3]*[Day=0]	22.5617557	11.57293575	1	1.000	-14.4363439	59.5598553
	[Condition=3]*[Day=1]	-21.5555822	13.80104985	1	1.000	-65.6768517	22.5656873
	[Condition=3]*[Day=2]	-35.7758715	15.00297218	1	.616	-83.7396268	12.1878838
[Condition=2]*[Day=2]	[Condition=1]*[Day=0]	63.4922103[a]	11.88733923	1	.000	25.4889784	101.4954422
	[Condition=1]*[Day=1]	26.9641180	14.03022480	1	1.000	-17.8898124	71.8180484
	[Condition=1]*[Day=2]	84.7342393[a]	14.96127115	1	.000	36.9038000	132.5646785
	[Condition=2]*[Day=0]	55.1217979[a]	11.94825844	1	.000	16.9238104	93.3197855
	[Condition=2]*[Day=1]	17.8003587	14.36602158	1	1.000	-28.1270973	63.7278147
	[Condition=3]*[Day=0]	40.3621145[a]	12.07451488	1	.030	1.7604914	78.9637376
	[Condition=3]*[Day=1]	-3.7552235	14.22427659	1	1.000	-49.2295278	41.7190808
	[Condition=3]*[Day=2]	-17.9755128	15.39318816	1	1.000	-67.1867692	31.2357436
[Condition=3]*[Day=0]	[Condition=1]*[Day=0]	23.1300958	8.29813834	1	.191	-3.3986394	49.6588311
	[Condition=1]*[Day=1]	-13.3979965	11.15336155	1	1.000	-49.0547382	22.2587453
	[Condition=1]*[Day=2]	44.3721248[a]	12.30406032	1	.011	5.0366563	83.7075933
	[Condition=2]*[Day=0]	14.7596835	8.38517415	1	1.000	-12.0473009	41.5666679
	[Condition=2]*[Day=1]	-22.5617557	11.57293575	1	1.000	-59.5598553	14.4363439
	[Condition=2]*[Day=2]	-40.3621145[a]	12.07451488	1	.030	-78.9637376	-1.7604914
	[Condition=3]*[Day=1]	-44.1173380[a]	11.39650431	1	.004	-80.5513950	-7.6832809

	[Condition=3]*[Day=2]	-58.3376272[a]	12.82577512	1	.000	-99.3409920	-17.3342625
[Condition=3]*[Day=1]	[Condition=1]*[Day=0]	67.2474338[a]	11.19800138	1	.000	31.4479807	103.0468869
	[Condition=1]*[Day=1]	30.7193415	13.45115642	1	.806	-12.2833361	73.7220191
	[Condition=1]*[Day=2]	88.4894628[a]	14.41964062	1	.000	42.3905894	134.5883361
	[Condition=2]*[Day=0]	58.8770214[a]	11.26264981	1	.000	22.8708906	94.8831523
	[Condition=2]*[Day=1]	21.5555822	13.80104985	1	1.000	-22.5656873	65.6768517
	[Condition=2]*[Day=2]	3.7552235	14.22427659	1	1.000	-41.7190808	49.2295278
	[Condition=3]*[Day=0]	44.1173380[a]	11.39650431	1	.004	7.6832809	80.5513950
	[Condition=3]*[Day=2]	-14.2202893	14.86730112	1	1.000	-61.7503110	33.3097324
[Condition=3]*[Day=2]	[Condition=1]*[Day=0]	81.4677231[a]	12.64972063	1	.000	41.0271958	121.9082503
	[Condition=1]*[Day=1]	44.9396308	14.68175078	1	.079	-1.9971957	91.8764573
	[Condition=1]*[Day=2]	102.7097520[a]	15.57389587	1	.000	52.9207821	152.4987220
	[Condition=2]*[Day=0]	73.0973107[a]	12.70698539	1	.000	32.4737109	113.7209106
	[Condition=2]*[Day=1]	35.7758715	15.00297218	1	.616	-12.1878838	83.7396268
	[Condition=2]*[Day=2]	17.9755128	15.39318816	1	1.000	-31.2357436	67.1867692
	[Condition=3]*[Day=0]	58.3376272[a]	12.82577512	1	.000	17.3342625	99.3409920
	[Condition=3]*[Day=1]	14.2202893	14.86730112	1	1.000	-33.3097324	61.7503110

Pairwise comparisons of estimated marginal means based on the original scale of dependent variable TTP

a. The mean difference is significant at the .05 level.

Overall Test Results

Wald Chi-Square	df	Sig.
109.841	8	.000

The Wald chi-square tests the effect of Condition*Day. This test is based on the linearly independent pairwise comparisons among the estimated marginal means.

Sarcomere Length Shortening Velocity [μm/s]

```
MEANS TABLES=ShorteningVel BY Condition BY Day
  /CELLS=MEAN COUNT STDDEV MIN MAX.
```

Means

Case Processing Summary

	Cases					
	Included		Excluded		Total	
	N	Percent	N	Percent	N	Percent
ShorteningVel * Condition * Day	654	97.3%	18	2.7%	672	100.0%

Report

ShorteningVel

Condition	Day	Mean	N	Std. Deviation	Minimum	Maximum
1	0	-1.7938314	139	.90434863	-4.01561	-.28560
	1	-1.2233570	51	1.06954176	-4.33190	-.05047
	2	-1.1402383	39	1.10126488	-4.90000	-.09064
	Total	-1.5554719	229	1.01823628	-4.90000	-.05047
2	0	-1.7325483	132	.79562605	-3.66000	-.33967
	1	-.7157374	46	.69695329	-3.46643	-.04576
	2	-.6731909	41	.65612975	-2.78185	-.05395
	Total	-1.3206444	219	.90467495	-3.66000	-.04576
3	0	-1.3164372	122	.80817161	-3.54092	-.05020
	1	-.5553690	48	.76299844	-3.34029	-.04300
	2	-.3561410	36	.35723919	-1.65293	-.03767

	Total	-.9712822	206	.84771804	-3.54092	-.03767
Total	0	-1.6250490	393	.86296126	-4.01561	-.05020
	1	-.8411920	145	.90718649	-4.33190	-.04300
	2	-.7318207	116	.83144686	-4.90000	-.03767
	Total	-1.2928262	654	.95807527	-4.90000	-.03767

```
* Generalized Linear Models.
GENLIN ShorteningVel BY Condition Day (ORDER=ASCENDING)
  /MODEL Condition Day Condition*Day INTERCEPT=YES
 DISTRIBUTION=NORMAL LINK=IDENTITY
  /CRITERIA SCALE=MLE COVB=MODEL PCONVERGE=1E-006(ABSOLUTE) SINGULAR=1E-012 ANALYSISTYPE=3(WALD)
    CILEVEL=95 CITYPE=WALD LIKELIHOOD=FULL
  /EMMEANS TABLES=Condition SCALE=ORIGINAL COMPARE=Condition CONTRAST=PAIRWISE PADJUST=BONFERRONI
  /EMMEANS TABLES=Day SCALE=ORIGINAL COMPARE=Day CONTRAST=PAIRWISE PADJUST=BONFERRONI
  /MISSING CLASSMISSING=EXCLUDE
  /PRINT CPS DESCRIPTIVES MODELINFO FIT SUMMARY SOLUTION.
```

Generalized Linear Models

Model Information

Dependent Variable	ShorteningVel
Probability Distribution	Normal
Link Function	Identity

Case Processing Summary

	N	Percent
Included	654	97.3%

Excluded	18	2.7%
Total	672	100.0%

Categorical Variable Information

			N	Percent
Factor	Condition	1	229	35.0%
		2	219	33.5%
		3	206	31.5%
		Total	654	100.0%
	Day	0	393	60.1%
		1	145	22.2%
		2	116	17.7%
		Total	654	100.0%

Continuous Variable Information

		N	Minimum	Maximum	Mean	Std. Deviation
Dependent Variable	ShorteningVel	654	-4.90000	-.03767	-1.2928262	.95807527

Goodness of Fit[a]

	Value	df	Value/df
Deviance	449.008	645	.696
Scaled Deviance	654.000	645	
Pearson Chi-Square	449.008	645	.696
Scaled Pearson Chi-Square	654.000	645	
Log Likelihood[b]	-805.012		
Akaike's Information Criterion (AIC)	1630.024		

Finite Sample Corrected AIC (AICC)	1630.366		
Bayesian Information Criterion (BIC)	1674.855		
Consistent AIC (CAIC)	1684.855		

Dependent Variable: ShorteningVel
Model: (Intercept), Condition, Day, Condition * Day
a. Information criteria are in smaller-is-better form.
b. The full log likelihood function is displayed and used in computing information criteria.

Omnibus Test[a]

Likelihood Ratio Chi-Square	df	Sig.
188.927	8	.000

Dependent Variable: ShorteningVel
Model: (Intercept), Condition, Day, Condition * Day
a. Compares the fitted model against the intercept-only model.

Tests of Model Effects

Source	Type III Wald Chi-Square	df	Sig.
(Intercept)	807.843	1	.000
Condition	49.851	2	.000
Day	158.414	2	.000
Condition * Day	7.533	4	.110

Dependent Variable: ShorteningVel

Model: (Intercept), Condition, Day, Condition * Day

Parameter Estimates

Parameter	B	Std. Error	95% Wald Confidence Interval		Hypothesis Test		
			Lower	Upper	Wald Chi-Square	df	Sig.
(Intercept)	-.356	.1381	-.627	-.085	6.651	1	.010
[Condition=1]	-.784	.1915	-1.159	-.409	16.764	1	.000
[Condition=2]	-.317	.1893	-.688	.054	2.807	1	.094
[Condition=3]	0[a]	.	.	.	.	.	.
[Day=0]	-.960	.1572	-1.268	-.652	37.337	1	.000
[Day=1]	-.199	.1827	-.557	.159	1.189	1	.275
[Day=2]	0[a]	.	.	.	.	.	.
[Condition=1] * [Day=0]	.307	.2174	-.119	.733	1.991	1	.158
[Condition=1] * [Day=1]	.116	.2539	-.381	.614	.209	1	.647
[Condition=1] * [Day=2]	0[a]	.	.	.	.	.	.
[Condition=2] * [Day=0]	-.099	.2160	-.522	.324	.210	1	.646
[Condition=2] * [Day=1]	.157	.2550	-.343	.657	.377	1	.539
[Condition=2] * [Day=2]	0[a]	.	.	.	.	.	.
[Condition=3] * [Day=0]	0[a]	.	.	.	.	.	.
[Condition=3] * [Day=1]	0[a]	.	.	.	.	.	.
[Condition=3] * [Day=2]	0[a]	.	.	.	.	.	.
(Scale)	.687[b]	.0380	.616	.765			

Dependent Variable: ShorteningVel

Model: (Intercept), Condition, Day, Condition * Day

a. Set to zero because this parameter is redundant.

b. Maximum likelihood estimate.

Estimated Marginal Means 1: Condition

Estimates

Condition	Mean	Std. Error	95% Wald Confidence Interval Lower	95% Wald Confidence Interval Upper
1	-1.3858089	.06325007	-1.5097767	-1.2618410
2	-1.0404922	.06400658	-1.1659428	-.9150416
3	-.7426491	.06582955	-.8716726	-.6136255

Pairwise Comparisons

(I) Condition	(J) Condition	Mean Difference (I-J)	Std. Error	df	Bonferroni Sig.	95% Wald Confidence Interval for Difference Lower	95% Wald Confidence Interval for Difference Upper
1	2	-.3453167[a]	.08998563	1	.000	-.5607405	-.1298929
	3	-.6431598[a]	.09129129	1	.000	-.8617093	-.4246103
2	1	.3453167[a]	.08998563	1	.000	.1298929	.5607405
	3	-.2978431[a]	.09181706	1	.004	-.5176513	-.0780350
3	1	.6431598[a]	.09129129	1	.000	.4246103	.8617093
	2	.2978431[a]	.09181706	1	.004	.0780350	.5176513

Pairwise comparisons of estimated marginal means based on the original scale of dependent variable ShorteningVel

a. The mean difference is significant at the .05 level.

Overall Test Results

Wald Chi-Square	df	Sig.
49.851	2	.000

The Wald chi-square tests the effect of Condition. This test is based on the linearly independent pairwise comparisons among the estimated marginal means.

Estimated Marginal Means 2: Day

Estimates

Day	Mean	Std. Error	95% Wald Confidence Interval Lower	Upper
0	-1.6142723	.04185682	-1.6963102	-1.5322344
1	-.8314878	.06887208	-.9664746	-.6965010
2	-.7231901	.07704275	-.8741911	-.5721891

Pairwise Comparisons

(I) Day	(J) Day	Mean Difference (I-J)	Std. Error	df	Bonferroni Sig.	95% Wald Confidence Interval for Difference Lower	Upper
0	1	-.7827845[a]	.08059378	1	.000	-.9757244	-.5898446
	2	-.8910822[a]	.08767884	1	.000	-1.1009836	-.6811808
1	0	.7827845[a]	.08059378	1	.000	.5898446	.9757244
	2	-.1082977	.10333900	1	.884	-.3556892	.1390938
2	0	.8910822[a]	.08767884	1	.000	.6811808	1.1009836
	1	.1082977	.10333900	1	.884	-.1390938	.3556892

Pairwise comparisons of estimated marginal means based on the original scale of dependent variable ShorteningVel

a. The mean difference is significant at the .05 level.

Overall Test Results

Wald Chi-Square	df	Sig.
158.414	2	.000

The Wald chi-square tests the effect of Day. This test is based on the linearly independent pairwise comparisons among the estimated marginal means.

Sarcomere Length Relengthening Velocity [μm/s]

```
MEANS TABLES=RelengthVel BY Condition BY Day
  /CELLS=MEAN COUNT STDDEV MIN MAX.
```

Means

Case Processing Summary

	Cases					
	Included		Excluded		Total	
	N	Percent	N	Percent	N	Percent
Relengthening Velocity * Condition * Day	656	97.6%	16	2.4%	672	100.0%

Report

Relengthening Velocity

Condition	Day	Mean	N	Std. Deviation	Minimum	Maximum
1	0	1.2547420	139	.81354951	.07664	3.83355
	1	.7564277	51	.81258881	.00947	2.69967
	2	.6468537	39	.76940939	.01364	3.38775
	Total	1.0402369	229	.84652713	.00947	3.83355
2	0	1.1712303	133	.71719988	.13240	3.60680
	1	.3660651	46	.59691016	.00812	3.46064
	2	.3744768	41	.56760579	.01240	2.28445
	Total	.8543917	220	.77218186	.00812	3.60680
3	0	.8092857	122	.67460561	.01095	2.62688

	1	.3009716	48	.59234154	.01006	2.92995
	2	.1344574	37	.19585243	.00927	.93171
	Total	.5707943	207	.66250517	.00927	2.92995
Total	0	1.0886183	394	.76261873	.01095	3.83355
	1	.4818168	145	.70499982	.00812	3.46064
	2	.3893655	117	.60001857	.00927	3.38775
	Total	.8297787	656	.78976772	.00812	3.83355

```
* Generalized Linear Models.
GENLIN RelengthVel BY Condition Day (ORDER=ASCENDING)
  /MODEL Condition Day Condition*Day INTERCEPT=YES
 DISTRIBUTION=NORMAL LINK=IDENTITY
  /CRITERIA SCALE=MLE COVB=MODEL PCONVERGE=1E-006(ABSOLUTE) SINGULAR=1E-012 ANALYSISTYPE=3(WALD)
    CILEVEL=95 CITYPE=WALD LIKELIHOOD=FULL
  /EMMEANS TABLES=Condition SCALE=ORIGINAL COMPARE=Condition CONTRAST=PAIRWISE PADJUST=BONFERRONI
  /EMMEANS TABLES=Day SCALE=ORIGINAL COMPARE=Day CONTRAST=PAIRWISE PADJUST=BONFERRONI
  /MISSING CLASSMISSING=EXCLUDE
  /PRINT CPS DESCRIPTIVES MODELINFO FIT SUMMARY SOLUTION.
```

Generalized Linear Models

Model Information

Dependent Variable	Relengthening Velocity
Probability Distribution	Normal
Link Function	Identity

Case Processing Summary

	N	Percent
Included	656	97.6%
Excluded	16	2.4%
Total	672	100.0%

Categorical Variable Information

			N	Percent
Factor	Condition	1	229	34.9%
		2	220	33.5%
		3	207	31.6%
		Total	656	100.0%
	Day	0	394	60.1%
		1	145	22.1%
		2	117	17.8%
		Total	656	100.0%

Continuous Variable Information

		N	Minimum	Maximum	Mean	Std. Deviation
Dependent Variable	Relengthening Velocity	656	.00812	3.83355	.8297787	.78976772

Goodness of Fit[a]

	Value	df	Value/df
Deviance	316.604	647	.489
Scaled Deviance	656.000	647	
Pearson Chi-Square	316.604	647	.489
Scaled Pearson Chi-Square	656.000	647	
Log Likelihood[b]	-691.873		

Akaike's Information Criterion (AIC)	1403.745		
Finite Sample Corrected AIC (AICC)	1404.086		
Bayesian Information Criterion (BIC)	1448.607		
Consistent AIC (CAIC)	1458.607		

Dependent Variable: Relengthening Velocity
Model: (Intercept), Condition, Day, Condition * Day
a. Information criteria are in smaller-is-better form.
b. The full log likelihood function is displayed and used in computing information criteria.

Omnibus Test[a]

Likelihood Ratio Chi-Square	df	Sig.
167.248	8	.000

Dependent Variable: Relengthening Velocity
Model: (Intercept), Condition, Day, Condition * Day
a. Compares the fitted model against the intercept-only model.

Tests of Model Effects

	Type III		
Source	Wald Chi-Square	df	Sig.
(Intercept)	432.017	1	.000
Condition	38.247	2	.000
Day	135.894	2	.000

Condition * Day	4.788	4	.310

Dependent Variable: Relengthening Velocity

Model: (Intercept), Condition, Day, Condition * Day

Parameter Estimates

Parameter	B	Std. Error	95% Wald Confidence Interval Lower	95% Wald Confidence Interval Upper	Hypothesis Test Wald Chi-Square	Hypothesis Test df	Hypothesis Test Sig.
(Intercept)	.134	.1142	-.089	.358	1.386	1	.239
[Condition=1]	.512	.1594	.200	.825	10.329	1	.001
[Condition=2]	.240	.1575	-.069	.549	2.322	1	.128
[Condition=3]	0[a]	.	.	.	.	.	.
[Day=0]	.675	.1304	.419	.930	26.788	1	.000
[Day=1]	.167	.1520	-.131	.464	1.200	1	.273
[Day=2]	0[a]	.	.	.	.	.	.
[Condition=1] * [Day=0]	-.067	.1812	-.422	.288	.136	1	.712
[Condition=1] * [Day=1]	-.057	.2120	-.472	.359	.072	1	.788
[Condition=1] * [Day=2]	0[a]	.	.	.	.	.	.
[Condition=2] * [Day=0]	.122	.1800	-.231	.475	.459	1	.498
[Condition=2] * [Day=1]	-.175	.2130	-.592	.243	.675	1	.411
[Condition=2] * [Day=2]	0[a]	.	.	.	.	.	.
[Condition=3] * [Day=0]	0[a]	.	.	.	.	.	.
[Condition=3] * [Day=1]	0[a]	.	.	.	.	.	.
[Condition=3] * [Day=2]	0[a]	.	.	.	.	.	.
(Scale)	.483[b]	.0266	.433	.538			

Dependent Variable: Relengthening Velocity

Model: (Intercept), Condition, Day, Condition * Day

a. Set to zero because this parameter is redundant.

b. Maximum likelihood estimate.

Estimated Marginal Means 1: Condition

Estimates

Condition	Mean	Std. Error	95% Wald Confidence Interval	
			Lower	Upper
1	.8860078	.05303092	.7820691	.9899465
2	.6372574	.05363675	.5321313	.7423835
3	.4149049	.05482772	.3074445	.5223652

Pairwise Comparisons

(I) Condition	(J) Condition	Mean Difference (I-J)	Std. Error	df	Bonferroni Sig.	95% Wald Confidence Interval for Difference	
						Lower	Upper
1	2	.2487504[a]	.07542665	1	.003	.0681806	.4293203
	3	.4711029[a]	.07627816	1	.000	.2884945	.6537113
2	1	-.2487504[a]	.07542665	1	.003	-.4293203	-.0681806
	3	.2223525[a]	.07670058	1	.011	.0387329	.4059721
3	1	-.4711029[a]	.07627816	1	.000	-.6537113	-.2884945
	2	-.2223525[a]	.07670058	1	.011	-.4059721	-.0387329

Pairwise comparisons of estimated marginal means based on the original scale of dependent variable Relengthening Velocity

a. The mean difference is significant at the .05 level.

Overall Test Results

Wald Chi-Square	df	Sig.

38.247	2	.000

The Wald chi-square tests the effect of Condition. This test is based on the linearly independent pairwise comparisons among the estimated marginal means.

Estimated Marginal Means 2: Day

Estimates

Day	Mean	Std. Error	95% Wald Confidence Interval Lower	Upper
0	1.0784193	.03505058	1.0097214	1.1471172
1	.4744881	.05774460	.3613108	.5876655
2	.3852626	.06428278	.2592707	.5112546

Pairwise Comparisons

(I) Day	(J) Day	Mean Difference (I-J)	Std. Error	df	Bonferroni Sig.	95% Wald Confidence Interval for Difference Lower	Upper
0	1	.6039312[a]	.06754985	1	.000	.4422182	.7656442
	2	.6931567[a]	.07321762	1	.000	.5178752	.8684382
1	0	-.6039312[a]	.06754985	1	.000	-.7656442	-.4422182
	2	.0892255	.08641016	1	.905	-.1176387	.2960897
2	0	-.6931567[a]	.07321762	1	.000	-.8684382	-.5178752
	1	-.0892255	.08641016	1	.905	-.2960897	.1176387

Pairwise comparisons of estimated marginal means based on the original scale of dependent variable Relengthening Velocity

a. The mean difference is significant at the .05 level.

Overall Test Results

Wald Chi-Square	df	Sig.
135.894	2	.000

The Wald chi-square tests the effect of Day. This test is based on the linearly independent pairwise comparisons among the estimated marginal means.

Normalized Sarcomere Length Shortening Velocity

```
MEANS TABLES=NormShortVel BY Condition BY Day
  /CELLS=MEAN COUNT STDDEV MIN MAX.
```

Means

Case Processing Summary

	Cases					
	Included		Excluded		Total	
	N	Percent	N	Percent	N	Percent
NormShortVel * Condition * Day	652	97.0%	20	3.0%	672	100.0%

Report

NormShortVel

Condition	Day	Mean	N	Std. Deviation	Minimum	Maximum
1	0	-10.7720	139	1.85502	-19.41	-7.15
	1	-9.9481	51	3.78394	-26.00	-3.64
	2	-11.2303	39	3.10784	-22.79	-5.37
	Total	-10.6665	229	2.64747	-26.00	-3.64
2	0	-10.7192	132	2.10204	-18.70	-7.07
	1	-9.0634	45	2.11331	-14.34	-5.58
	2	-8.6177	41	1.84193	-14.11	-5.05
	Total	-9.9822	218	2.24848	-18.70	-5.05
3	0	-9.7210	122	1.81816	-16.50	-4.64
	1	-8.2373	48	2.39571	-12.28	-3.23
	2	-8.2036	35	2.35856	-12.71	-3.27

	Total	-9.1145	205	2.18028	-16.50	-3.23
Total	0	-10.4280	393	1.98355	-19.41	-4.64
	1	-9.1013	144	2.96204	-26.00	-3.23
	2	-9.3777	115	2.80611	-22.79	-3.27
	Total	-9.9497	652	2.45569	-26.00	-3.23

```
* Generalized Linear Models.
GENLIN NormShortVel BY Condition Day (ORDER=ASCENDING)
  /MODEL Condition Day Condition*Day INTERCEPT=YES
 DISTRIBUTION=NORMAL LINK=IDENTITY
  /CRITERIA SCALE=MLE COVB=MODEL PCONVERGE=1E-006(ABSOLUTE) SINGULAR=1E-012 ANALYSISTYPE=3(WALD)
    CILEVEL=95 CITYPE=WALD LIKELIHOOD=FULL
  /EMMEANS TABLES=Condition SCALE=ORIGINAL COMPARE=Condition CONTRAST=PAIRWISE PADJUST=BONFERRONI
  /EMMEANS TABLES=Day SCALE=ORIGINAL COMPARE=Day CONTRAST=PAIRWISE PADJUST=BONFERRONI
  /EMMEANS TABLES=Condition*Day SCALE=ORIGINAL COMPARE=Condition*Day CONTRAST=PAIRWISE
    PADJUST=BONFERRONI
  /MISSING CLASSMISSING=EXCLUDE
  /PRINT CPS DESCRIPTIVES MODELINFO FIT SUMMARY SOLUTION.
```

Generalized Linear Models

Model Information

Dependent Variable	NormShortVel
Probability Distribution	Normal
Link Function	Identity

Case Processing Summary

	N	Percent

Included	652	97.0%
Excluded	20	3.0%
Total	672	100.0%

Categorical Variable Information

			N	Percent
Factor	Condition	1	229	35.1%
		2	218	33.4%
		3	205	31.4%
		Total	652	100.0%
	Day	0	393	60.3%
		1	144	22.1%
		2	115	17.6%
		Total	652	100.0%

Continuous Variable Information

		N	Minimum	Maximum	Mean	Std. Deviation
Dependent Variable	NormShortVel	652	-26.00	-3.23	-9.9497	2.45569

Goodness of Fit[a]

	Value	df	Value/df
Deviance	3327.738	643	5.175
Scaled Deviance	652.000	643	
Pearson Chi-Square	3327.738	643	5.175
Scaled Pearson Chi-Square	652.000	643	
Log Likelihood[b]	-1456.529		

Akaike's Information Criterion (AIC)	2933.058		
Finite Sample Corrected AIC (AICC)	2933.401		
Bayesian Information Criterion (BIC)	2977.859		
Consistent AIC (CAIC)	2987.859		

Dependent Variable: NormShortVel

Model: (Intercept), Condition, Day, Condition * Day

a. Information criteria are in smaller-is-better form.

b. The full log likelihood function is displayed and used in computing information criteria.

Omnibus Test[a]

Likelihood Ratio Chi-Square	df	Sig.
107.759	8	.000

Dependent Variable: NormShortVel

Model: (Intercept), Condition, Day, Condition * Day

a. Compares the fitted model against the intercept-only model.

Tests of Model Effects

	Type III		
Source	Wald Chi-Square	df	Sig.
(Intercept)	8928.873	1	.000
Condition	61.161	2	.000
Day	44.979	2	.000

Condition * Day	21.806	4	.000

Dependent Variable: NormShortVel

Model: (Intercept), Condition, Day, Condition * Day

Parameter Estimates

			95% Wald Confidence Interval		Hypothesis Test		
Parameter	B	Std. Error	Lower	Upper	Wald Chi-Square	df	Sig.
(Intercept)	-8.204	.3819	-8.952	-7.455	461.500	1	.000
[Condition=1]	-3.027	.5260	-4.058	-1.996	33.110	1	.000
[Condition=2]	-.414	.5199	-1.433	.605	.634	1	.426
[Condition=3]	0[a]	.	.	.	.	.	.
[Day=0]	-1.517	.4332	-2.367	-.668	12.271	1	.000
[Day=1]	-.034	.5022	-1.018	.950	.005	1	.946
[Day=2]	0[a]	.	.	.	.	.	.
[Condition=1] * [Day=0]	1.976	.5960	.808	3.144	10.989	1	.001
[Condition=1] * [Day=1]	1.316	.6951	-.046	2.678	3.585	1	.058
[Condition=1] * [Day=2]	0[a]	.	.	.	.	.	.
[Condition=2] * [Day=0]	-.584	.5923	-1.745	.577	.972	1	.324
[Condition=2] * [Day=1]	-.412	.7000	-1.784	.960	.346	1	.556
[Condition=2] * [Day=2]	0[a]	.	.	.	.	.	.
[Condition=3] * [Day=0]	0[a]	.	.	.	.	.	.
[Condition=3] * [Day=1]	0[a]	.	.	.	.	.	.
[Condition=3] * [Day=2]	0[a]	.	.	.	.	.	.
(Scale)	5.104[b]	.2827	4.579	5.689			

Dependent Variable: NormShortVel

Model: (Intercept), Condition, Day, Condition * Day

a. Set to zero because this parameter is redundant.

b. Maximum likelihood estimate.

Estimated Marginal Means 1: Condition

Estimates

Condition	Mean	Std. Error	95% Wald Confidence Interval	
			Lower	Upper
1	-10.6501	.17245	-10.9881	-10.3121
2	-9.4668	.17530	-9.8104	-9.1232
3	-8.7206	.18074	-9.0749	-8.3664

Pairwise Comparisons

(I) Condition	(J) Condition	Mean Difference (I-J)	Std. Error	df	Bonferroni Sig.	95% Wald Confidence Interval for Difference	
						Lower	Upper
1	2	-1.1834[a]	.24591	1	.000	-1.7721	-.5947
	3	-1.9295[a]	.24981	1	.000	-2.5275	-1.3315
2	1	1.1834[a]	.24591	1	.000	.5947	1.7721
	3	-.7461[a]	.25179	1	.009	-1.3489	-.1434
3	1	1.9295[a]	.24981	1	.000	1.3315	2.5275
	2	.7461[a]	.25179	1	.009	.1434	1.3489

Pairwise comparisons of estimated marginal means based on the original scale of dependent variable NormShortVel

a. The mean difference is significant at the .05 level.

Overall Test Results

Wald Chi-Square	df	Sig.

61.161	2	.000

The Wald chi-square tests the effect of Condition. This test is based on the linearly independent pairwise comparisons among the estimated marginal means.

Estimated Marginal Means 2: Day

Estimates

Day	Mean	Std. Error	95% Wald Confidence Interval Lower	95% Wald Confidence Interval Upper
0	-10.4041	.11412	-10.6277	-10.1804
1	-9.0829	.18851	-9.4524	-8.7134
2	-9.3505	.21113	-9.7643	-8.9367

Pairwise Comparisons

(I) Day	(J) Day	Mean Difference (I-J)	Std. Error	df	Bonferroni Sig.	95% Wald Confidence Interval for Difference Lower	95% Wald Confidence Interval for Difference Upper
0	1	-1.3211[a]	.22037	1	.000	-1.8487	-.7936
	2	-1.0535[a]	.24000	1	.000	-1.6281	-.4790
1	0	1.3211[a]	.22037	1	.000	.7936	1.8487
	2	.2676	.28304	1	1.000	-.4100	.9452
2	0	1.0535[a]	.24000	1	.000	.4790	1.6281
	1	-.2676	.28304	1	1.000	-.9452	.4100

Pairwise comparisons of estimated marginal means based on the original scale of dependent variable NormShortVel

a. The mean difference is significant at the .05 level.

Overall Test Results

Wald Chi-Square	df	Sig.
44.979	2	.000

The Wald chi-square tests the effect of Day. This test is based on the linearly independent pairwise comparisons among the estimated marginal means.

Estimated Marginal Means 3: Condition* Day

Estimates

				95% Wald Confidence Interval	
Condition	Day	Mean	Std. Error	Lower	Upper
1	0	-10.7720	.19162	-11.1475	-10.3964
	1	-9.9481	.31635	-10.5681	-9.3280
	2	-11.2303	.36176	-11.9394	-10.5213
2	0	-10.7192	.19664	-11.1046	-10.3338
	1	-9.0634	.33678	-9.7235	-8.4034
	2	-8.6177	.35282	-9.3092	-7.9261
3	0	-9.7210	.20454	-10.1219	-9.3201
	1	-8.2373	.32608	-8.8764	-7.5982
	2	-8.2036	.38187	-8.9520	-7.4551

Pairwise Comparisons

(I) Condition*Day	(J) Condition*Day	Mean Difference (I-J)	Std. Error	df	Bonferroni Sig.	95% Wald Confidence Interval for Difference	
						Lower	Upper
[Condition=1]*[Day=0]	[Condition=1]*[Day=1]	-.8239	.36986	1	.933	-2.0063	.3585
	[Condition=1]*[Day=2]	.4584	.40937	1	1.000	-.8504	1.7671
	[Condition=2]*[Day=0]	-.0528	.27456	1	1.000	-.9305	.8250
	[Condition=2]*[Day=1]	-1.7085[a]	.38748	1	.000	-2.9473	-.4698
	[Condition=2]*[Day=2]	-2.1543[a]	.40150	1	.000	-3.4379	-.8707
	[Condition=3]*[Day=0]	-1.0509[a]	.28027	1	.006	-1.9470	-.1549
	[Condition=3]*[Day=1]	-2.5347[a]	.37822	1	.000	-3.7438	-1.3256
	[Condition=3]*[Day=2]	-2.5684[a]	.42725	1	.000	-3.9343	-1.2025
[Condition=1]*[Day=1]	[Condition=1]*[Day=0]	.8239	.36986	1	.933	-.3585	2.0063
	[Condition=1]*[Day=2]	1.2823	.48057	1	.274	-.2541	2.8186
	[Condition=2]*[Day=0]	.7712	.37248	1	1.000	-.4197	1.9620
	[Condition=2]*[Day=1]	-.8846	.46206	1	1.000	-2.3618	.5925
	[Condition=2]*[Day=2]	-1.3304	.47388	1	.180	-2.8454	.1846
	[Condition=3]*[Day=0]	-.2270	.37671	1	1.000	-1.4314	.9773
	[Condition=3]*[Day=1]	-1.7108[a]	.45432	1	.006	-3.1632	-.2584
	[Condition=3]*[Day=2]	-1.7445[a]	.49588	1	.016	-3.3298	-.1592
[Condition=1]*[Day=2]	[Condition=1]*[Day=0]	-.4584	.40937	1	1.000	-1.7671	.8504
	[Condition=1]*[Day=1]	-1.2823	.48057	1	.274	-2.8186	.2541
	[Condition=2]*[Day=0]	-.5111	.41175	1	1.000	-1.8275	.8052
	[Condition=2]*[Day=1]	-2.1669[a]	.49426	1	.000	-3.7470	-.5868
	[Condition=2]*[Day=2]	-2.6127[a]	.50533	1	.000	-4.2282	-.9972
	[Condition=3]*[Day=0]	-1.5093[a]	.41558	1	.010	-2.8379	-.1807
	[Condition=3]*[Day=1]	-2.9931[a]	.48703	1	.000	-4.5501	-1.4361
	[Condition=3]*[Day=2]	-3.0268[a]	.52602	1	.000	-4.7084	-1.3451

[Condition=2]*[Day=0]	[Condition=1]*[Day=0]	.0528	.27456	1	1.000	-.8250	.9305
	[Condition=1]*[Day=1]	-.7712	.37248	1	1.000	-1.9620	.4197
	[Condition=1]*[Day=2]	.5111	.41175	1	1.000	-.8052	1.8275
	[Condition=2]*[Day=1]	-1.6558[a]	.38998	1	.001	-2.9025	-.4090
	[Condition=2]*[Day=2]	-2.1016[a]	.40392	1	.000	-3.3929	-.8102
	[Condition=3]*[Day=0]	-.9982[a]	.28373	1	.016	-1.9052	-.0911
	[Condition=3]*[Day=1]	-2.4819[a]	.38078	1	.000	-3.6993	-1.2646
	[Condition=3]*[Day=2]	-2.5156[a]	.42952	1	.000	-3.8888	-1.1425
[Condition=2]*[Day=1]	[Condition=1]*[Day=0]	1.7085[a]	.38748	1	.000	.4698	2.9473
	[Condition=1]*[Day=1]	.8846	.46206	1	1.000	-.5925	2.3618
	[Condition=1]*[Day=2]	2.1669[a]	.49426	1	.000	.5868	3.7470
	[Condition=2]*[Day=0]	1.6558[a]	.38998	1	.001	.4090	2.9025
	[Condition=2]*[Day=2]	-.4458	.48776	1	1.000	-2.0051	1.1136
	[Condition=3]*[Day=0]	.6576	.39402	1	1.000	-.6021	1.9173
	[Condition=3]*[Day=1]	-.8262	.46878	1	1.000	-2.3248	.6725
	[Condition=3]*[Day=2]	-.8599	.50916	1	1.000	-2.4876	.7679
[Condition=2]*[Day=2]	[Condition=1]*[Day=0]	2.1543[a]	.40150	1	.000	.8707	3.4379
	[Condition=1]*[Day=1]	1.3304	.47388	1	.180	-.1846	2.8454
	[Condition=1]*[Day=2]	2.6127[a]	.50533	1	.000	.9972	4.2282
	[Condition=2]*[Day=0]	2.1016[a]	.40392	1	.000	.8102	3.3929
	[Condition=2]*[Day=1]	.4458	.48776	1	1.000	-1.1136	2.0051
	[Condition=3]*[Day=0]	1.1034	.40782	1	.246	-.2004	2.4072
	[Condition=3]*[Day=1]	-.3804	.48043	1	1.000	-1.9163	1.1555
	[Condition=3]*[Day=2]	-.4141	.51991	1	1.000	-2.0762	1.2480
[Condition=3]*[Day=0]	[Condition=1]*[Day=0]	1.0509[a]	.28027	1	.006	.1549	1.9470
	[Condition=1]*[Day=1]	.2270	.37671	1	1.000	-.9773	1.4314
	[Condition=1]*[Day=2]	1.5093[a]	.41558	1	.010	.1807	2.8379
	[Condition=2]*[Day=0]	.9982[a]	.28373	1	.016	.0911	1.9052
	[Condition=2]*[Day=1]	-.6576	.39402	1	1.000	-1.9173	.6021
	[Condition=2]*[Day=2]	-1.1034	.40782	1	.246	-2.4072	.2004

	[Condition=3]*[Day=1]	-1.4838[a]	.38492	1	.004	-2.7144	-.2532
	[Condition=3]*[Day=2]	-1.5175[a]	.43320	1	.017	-2.9024	-.1326
[Condition=3]*[Day=1]	[Condition=1]*[Day=0]	2.5347[a]	.37822	1	.000	1.3256	3.7438
	[Condition=1]*[Day=1]	1.7108[a]	.45432	1	.006	.2584	3.1632
	[Condition=1]*[Day=2]	2.9931[a]	.48703	1	.000	1.4361	4.5501
	[Condition=2]*[Day=0]	2.4819[a]	.38078	1	.000	1.2646	3.6993
	[Condition=2]*[Day=1]	.8262	.46878	1	1.000	-.6725	2.3248
	[Condition=2]*[Day=2]	.3804	.48043	1	1.000	-1.1555	1.9163
	[Condition=3]*[Day=0]	1.4838[a]	.38492	1	.004	.2532	2.7144
	[Condition=3]*[Day=2]	-.0337	.50215	1	1.000	-1.6391	1.5717
[Condition=3]*[Day=2]	[Condition=1]*[Day=0]	2.5684[a]	.42725	1	.000	1.2025	3.9343
	[Condition=1]*[Day=1]	1.7445[a]	.49588	1	.016	.1592	3.3298
	[Condition=1]*[Day=2]	3.0268[a]	.52602	1	.000	1.3451	4.7084
	[Condition=2]*[Day=0]	2.5156[a]	.42952	1	.000	1.1425	3.8888
	[Condition=2]*[Day=1]	.8599	.50916	1	1.000	-.7679	2.4876
	[Condition=2]*[Day=2]	.4141	.51991	1	1.000	-1.2480	2.0762
	[Condition=3]*[Day=0]	1.5175[a]	.43320	1	.017	.1326	2.9024
	[Condition=3]*[Day=1]	.0337	.50215	1	1.000	-1.5717	1.6391

Pairwise comparisons of estimated marginal means based on the original scale of dependent variable NormShortVel

a. The mean difference is significant at the .05 level.

Overall Test Results

Wald Chi-Square	df	Sig.
117.175	8	.000

The Wald chi-square tests the effect of Condition*Day. This test is based on the linearly independent pairwise comparisons among the estimated marginal means.

Normalized Sarcomere Length Relengthening Velocity

```
MEANS TABLES=NormRelengthVel BY Condition BY Day
  /CELLS=MEAN COUNT STDDEV MIN MAX.
```

Means

Case Processing Summary

	Cases					
	Included		Excluded		Total	
	N	Percent	N	Percent	N	Percent
NormRelengthVel * Condition * Day	654	97.3%	18	2.7%	672	100.0%

Report

NormRelengthVel

Condition	Day	Mean	N	Std. Deviation	Minimum	Maximum
1	0	7.0761315	139	2.81434979	1.55785	15.44781
	1	5.1239115	51	3.32331128	1.08036	16.36559
	2	5.3749142	39	3.21644624	1.19922	12.36880
	Total	6.3516306	229	3.12420247	1.08036	16.36559
2	0	6.9067178	133	2.58227599	2.70416	14.82362
	1	3.6168355	46	2.42170717	1.06897	13.75610
	2	3.3476999	41	2.40543752	.93233	10.31738
	Total	5.5555618	220	3.01527148	.93233	14.82362
3	0	5.2278864	122	2.58753291	.89493	11.19264

	1	2.9157413	48	2.30107029	.95102	10.68708
	2	2.3868860	35	1.38788226	.92336	6.45708
	Total	4.2014573	205	2.66385744	.89493	11.19264
Total	0	6.4466444	394	2.78482552	.89493	15.44781
	1	3.9148241	145	2.87656293	.95102	16.36559
	2	3.7427683	115	2.75410781	.92336	12.36880
	Total	5.4098559	654	3.07419289	.89493	16.36559

```
* Generalized Linear Models.
GENLIN NormRelengthVel BY Condition Day (ORDER=ASCENDING)
  /MODEL Condition Day Condition*Day INTERCEPT=YES
 DISTRIBUTION=NORMAL LINK=IDENTITY
  /CRITERIA SCALE=MLE COVB=MODEL PCONVERGE=1E-006(ABSOLUTE) SINGULAR=1E-012 ANALYSISTYPE=3(WALD)
    CILEVEL=95 CITYPE=WALD LIKELIHOOD=FULL
  /EMMEANS TABLES=Condition SCALE=ORIGINAL COMPARE=Condition CONTRAST=PAIRWISE PADJUST=BONFERRONI
  /EMMEANS TABLES=Day SCALE=ORIGINAL COMPARE=Day CONTRAST=PAIRWISE PADJUST=BONFERRONI
  /EMMEANS TABLES=Condition*Day SCALE=ORIGINAL COMPARE=Condition*Day CONTRAST=PAIRWISE
    PADJUST=BONFERRONI
  /MISSING CLASSMISSING=EXCLUDE
  /PRINT CPS DESCRIPTIVES MODELINFO FIT SUMMARY SOLUTION.
```

Generalized Linear Models

Model Information

Dependent Variable	NormRelengthVel
Probability Distribution	Normal
Link Function	Identity

Case Processing Summary

	N	Percent
Included	654	97.3%
Excluded	18	2.7%
Total	672	100.0%

Categorical Variable Information

			N	Percent
Factor	Condition	1	229	35.0%
		2	220	33.6%
		3	205	31.3%
		Total	654	100.0%
	Day	0	394	60.2%
		1	145	22.2%
		2	115	17.6%
		Total	654	100.0%

Continuous Variable Information

		N	Minimum	Maximum	Mean	Std. Deviation
Dependent Variable	NormRelengthVel	654	.89493	16.36559	5.4098559	3.07419289

Goodness of Fit[a]

	Value	df	Value/df
Deviance	4538.426	645	7.036
Scaled Deviance	654.000	645	

Pearson Chi-Square	4538.426	645	7.036
Scaled Pearson Chi-Square	654.000	645	
Log Likelihood[b]	-1561.459		
Akaike's Information Criterion (AIC)	3142.919		
Finite Sample Corrected AIC (AICC)	3143.261		
Bayesian Information Criterion (BIC)	3187.750		
Consistent AIC (CAIC)	3197.750		

Dependent Variable: NormRelengthVel

Model: (Intercept), Condition, Day, Condition * Day

a. Information criteria are in smaller-is-better form.

b. The full log likelihood function is displayed and used in computing information criteria.

Omnibus Test[a]

Likelihood Ratio Chi-Square	df	Sig.
200.991	8	.000

Dependent Variable: NormRelengthVel

Model: (Intercept), Condition, Day, Condition * Day

a. Compares the fitted model against the intercept-only model.

Tests of Model Effects

Source	Type III Wald Chi-Square	df	Sig.

(Intercept)	1551.172	1	.000
Condition	65.196	2	.000
Day	152.282	2	.000
Condition * Day	10.470	4	.033

Dependent Variable: NormRelengthVel

Model: (Intercept), Condition, Day, Condition * Day

Parameter Estimates

Parameter	B	Std. Error	95% Wald Confidence Interval Lower	95% Wald Confidence Interval Upper	Hypothesis Test Wald Chi-Square	Hypothesis Test df	Hypothesis Test Sig.
(Intercept)	2.387	.4453	1.514	3.260	28.735	1	.000
[Condition=1]	2.988	.6134	1.786	4.190	23.732	1	.000
[Condition=2]	.961	.6062	-.227	2.149	2.512	1	.113
[Condition=3]	0[a]	.	.	.	.	.	.
[Day=0]	2.841	.5051	1.851	3.831	31.633	1	.000
[Day=1]	.529	.5855	-.619	1.676	.816	1	.366
[Day=2]	0[a]	.	.	.	.	.	.
[Condition=1] * [Day=0]	-1.140	.6950	-2.502	.222	2.690	1	.101
[Condition=1] * [Day=1]	-.780	.8105	-2.368	.809	.926	1	.336
[Condition=1] * [Day=2]	0[a]	.	.	.	.	.	.
[Condition=2] * [Day=0]	.718	.6904	-.635	2.071	1.082	1	.298
[Condition=2] * [Day=1]	-.260	.8142	-1.856	1.336	.102	1	.750
[Condition=2] * [Day=2]	0[a]	.	.	.	.	.	.
[Condition=3] * [Day=0]	0[a]	.	.	.	.	.	.
[Condition=3] * [Day=1]	0[a]	.	.	.	.	.	.
[Condition=3] * [Day=2]	0[a]	.	.	.	.	.	.
(Scale)	6.939[b]	.3838	6.227	7.734			

Dependent Variable: NormRelengthVel

Model: (Intercept), Condition, Day, Condition * Day

a. Set to zero because this parameter is redundant.

b. Maximum likelihood estimate.

Estimated Marginal Means 1: Condition

Estimates

Condition	Mean	Std. Error	95% Wald Confidence Interval Lower	95% Wald Confidence Interval Upper
1	5.8583191	.20108827	5.4641933	6.2524448
2	4.6237511	.20338549	4.2251228	5.0223793
3	3.5101712	.21074600	3.0971167	3.9232258

Pairwise Comparisons

(I) Condition	(J) Condition	Mean Difference (I-J)	Std. Error	df	Bonferroni Sig.	95% Wald Confidence Interval for Difference Lower	95% Wald Confidence Interval for Difference Upper
1	2	1.2345680[a]	.28601075	1	.000	.5498640	1.9192720
	3	2.3481478[a]	.29129086	1	.000	1.6508034	3.0454923
2	1	-1.2345680[a]	.28601075	1	.000	-1.9192720	-.5498640
	3	1.1135798[a]	.29288143	1	.000	.4124276	1.8147320
3	1	-2.3481478[a]	.29129086	1	.000	-3.0454923	-1.6508034
	2	-1.1135798[a]	.29288143	1	.000	-1.8147320	-.4124276

Pairwise comparisons of estimated marginal means based on the original scale of dependent variable NormRelengthVel

a. The mean difference is significant at the .05 level.

Overall Test Results

Wald Chi-Square	df	Sig.
65.196	2	.000

The Wald chi-square tests the effect of Condition. This test is based on the linearly independent pairwise comparisons among the estimated marginal means.

Estimated Marginal Means 2: Day

Estimates

Day	Mean	Std. Error	95% Wald Confidence Interval Lower	95% Wald Confidence Interval Upper
0	6.4035786	.13290851	6.1430827	6.6640745
1	3.8854961	.21896209	3.4563383	4.3146539
2	3.7031667	.24618480	3.2206534	4.1856800

Pairwise Comparisons

(I) Day	(J) Day	Mean Difference (I-J)	Std. Error	df	Bonferroni Sig.	95% Wald Confidence Interval for Difference Lower	95% Wald Confidence Interval for Difference Upper
0	1	2.5180825[a]	.25614268	1	.000	1.9048821	3.1312829
	2	2.7004119[a]	.27977067	1	.000	2.0306465	3.3701772
1	0	-2.5180825[a]	.25614268	1	.000	-3.1312829	-1.9048821

	2	.1823294	.32947132	1	1.000	-.6064183	.9710771
2	0	-2.7004119[a]	.27977067	1	.000	-3.3701772	-2.0306465
	1	-.1823294	.32947132	1	1.000	-.9710771	.6064183

Pairwise comparisons of estimated marginal means based on the original scale of dependent variable NormRelengthVel

a. The mean difference is significant at the .05 level.

Overall Test Results

Wald Chi-Square	df	Sig.
152.282	2	.000

The Wald chi-square tests the effect of Day. This test is based on the linearly independent pairwise comparisons among the estimated marginal means.

Estimated Marginal Means 3: Condition* Day

Estimates

Condition	Day	Mean	Std. Error	95% Wald Confidence Interval Lower	Upper
1	0	7.0761315	.22343765	6.6382017	7.5140612
	1	5.1239115	.36887453	4.4009307	5.8468922
	2	5.3749142	.42182416	4.5481541	6.2016744
2	0	6.9067178	.22842200	6.4590189	7.3544167
	1	3.6168355	.38840503	2.8555757	4.3780954
	2	3.3476999	.41140714	2.5413567	4.1540430

3	0	5.2278864	.23849751	4.7604399	5.6953330
	1	2.9157413	.38022716	2.1705097	3.6609728
	2	2.3868860	.44527646	1.5141602	3.2596118

Pairwise Comparisons

(I) Condition*Day	(J) Condition*Day	Mean Difference (I-J)	Std. Error	df	Bonferroni Sig.	95% Wald Confidence Interval for Difference Lower	Upper
[Condition=1]*[Day=0]	[Condition=1]*[Day=1]	1.9522200[a]	.43126883	1	.000	.5734751	3.3309650
	[Condition=1]*[Day=2]	1.7012173[a]	.47734684	1	.013	.1751632	3.2272714
	[Condition=2]*[Day=0]	.1694137	.31953246	1	1.000	-.8521157	1.1909431
	[Condition=2]*[Day=1]	3.4592960[a]	.44808799	1	.000	2.0267809	4.8918110
	[Condition=2]*[Day=2]	3.7284316[a]	.46816687	1	.000	2.2317254	5.2251378
	[Condition=3]*[Day=0]	1.8482451[a]	.32681102	1	.000	.8034465	2.8930436
	[Condition=3]*[Day=1]	4.1603902[a]	.44101823	1	.000	2.7504769	5.5703035
	[Condition=3]*[Day=2]	4.6892455[a]	.49819224	1	.000	3.0965497	6.2819413
[Condition=1]*[Day=1]	[Condition=1]*[Day=0]	-1.9522200[a]	.43126883	1	.000	-3.3309650	-.5734751
	[Condition=1]*[Day=2]	-.2510028	.56036064	1	1.000	-2.0424478	1.5404423
	[Condition=2]*[Day=0]	-1.7828063[a]	.43387214	1	.001	-3.1698739	-.3957387
	[Condition=2]*[Day=1]	1.5070759	.53565556	1	.176	-.2053883	3.2195401
	[Condition=2]*[Day=2]	1.7762116[a]	.55256154	1	.047	.0096999	3.5427233
	[Condition=3]*[Day=0]	-.1039750	.43926015	1	1.000	-1.5082678	1.3003178
	[Condition=3]*[Day=1]	2.2081702[a]	.52975571	1	.001	.5145675	3.9017728
	[Condition=3]*[Day=2]	2.7370255[a]	.57822101	1	.000	.8884817	4.5855693
[Condition=1]*[Day=2]	[Condition=1]*[Day=0]	-1.7012173[a]	.47734684	1	.013	-3.2272714	-.1751632
	[Condition=1]*[Day=1]	.2510028	.56036064	1	1.000	-1.5404423	2.0424478
	[Condition=2]*[Day=0]	-1.5318035	.47970015	1	.051	-3.0653810	.0017740
	[Condition=2]*[Day=1]	1.7580787	.57340569	1	.078	-.0750708	3.5912281
	[Condition=2]*[Day=2]	2.0272144[a]	.58922955	1	.021	.1434768	3.9109519

	[Condition=3]*[Day=0]	.1470278	.48457887	1	1.000	-1.4021468	1.6962023
	[Condition=3]*[Day=1]	2.4591729[a]	.56789816	1	.001	.6436308	4.2747151
	[Condition=3]*[Day=2]	2.9880282[a]	.61335695	1	.000	1.0271566	4.9488999
[Condition=2]*[Day=0]	[Condition=1]*[Day=0]	-.1694137	.31953246	1	1.000	-1.1909431	.8521157
	[Condition=1]*[Day=1]	1.7828063[a]	.43387214	1	.001	.3957387	3.1698739
	[Condition=1]*[Day=2]	1.5318035	.47970015	1	.051	-.0017740	3.0653810
	[Condition=2]*[Day=1]	3.2898822[a]	.45059414	1	.000	1.8493552	4.7304092
	[Condition=2]*[Day=2]	3.5590179[a]	.47056609	1	.000	2.0546415	5.0633943
	[Condition=3]*[Day=0]	1.6788313[a]	.33023881	1	.000	.6230743	2.7345884
	[Condition=3]*[Day=1]	3.9909765[a]	.44356432	1	.000	2.5729234	5.4090295
	[Condition=3]*[Day=2]	4.5198318[a]	.50044753	1	.000	2.9199259	6.1197376
[Condition=2]*[Day=1]	[Condition=1]*[Day=0]	-3.4592960[a]	.44808799	1	.000	-4.8918110	-2.0267809
	[Condition=1]*[Day=1]	-1.5070759	.53565556	1	.176	-3.2195401	.2053883
	[Condition=1]*[Day=2]	-1.7580787	.57340569	1	.078	-3.5912281	.0750708
	[Condition=2]*[Day=0]	-3.2898822[a]	.45059414	1	.000	-4.7304092	-1.8493552
	[Condition=2]*[Day=2]	.2691357	.56578644	1	1.000	-1.5396554	2.0779268
	[Condition=3]*[Day=0]	-1.6110509[a]	.45578451	1	.015	-3.0681713	-.1539305
	[Condition=3]*[Day=1]	.7010943	.54353579	1	1.000	-1.0365626	2.4387511
	[Condition=3]*[Day=2]	1.2299496	.59087189	1	1.000	-.6590385	3.1189376
[Condition=2]*[Day=2]	[Condition=1]*[Day=0]	-3.7284316[a]	.46816687	1	.000	-5.2251378	-2.2317254
	[Condition=1]*[Day=1]	-1.7762116[a]	.55256154	1	.047	-3.5427233	-.0096999
	[Condition=1]*[Day=2]	-2.0272144[a]	.58922955	1	.021	-3.9109519	-.1434768
	[Condition=2]*[Day=0]	-3.5590179[a]	.47056609	1	.000	-5.0633943	-2.0546415
	[Condition=2]*[Day=1]	-.2691357	.56578644	1	1.000	-2.0779268	1.5396554
	[Condition=3]*[Day=0]	-1.8801866[a]	.47553853	1	.003	-3.4004596	-.3599136
	[Condition=3]*[Day=1]	.4319586	.56020401	1	1.000	-1.3589858	2.2229029
	[Condition=3]*[Day=2]	.9608139	.60624002	1	1.000	-.9773053	2.8989330
[Condition=3]*[Day=0]	[Condition=1]*[Day=0]	-1.8482451[a]	.32681102	1	.000	-2.8930436	-.8034465
	[Condition=1]*[Day=1]	.1039750	.43926015	1	1.000	-1.3003178	1.5082678
	[Condition=1]*[Day=2]	-.1470278	.48457887	1	1.000	-1.6962023	1.4021468

	[Condition=2]*[Day=0]	-1.6788313[a]	.33023881	1	.000	-2.7345884	-.6230743
	[Condition=2]*[Day=1]	1.6110509[a]	.45578451	1	.015	.1539305	3.0681713
	[Condition=2]*[Day=2]	1.8801866[a]	.47553853	1	.003	.3599136	3.4004596
	[Condition=3]*[Day=1]	2.3121451[a]	.44883600	1	.000	.8772388	3.7470515
	[Condition=3]*[Day=2]	2.8410005[a]	.50512591	1	.000	1.2261381	4.4558628
[Condition=3]*[Day=1]	[Condition=1]*[Day=0]	-4.1603902[a]	.44101823	1	.000	-5.5703035	-2.7504769
	[Condition=1]*[Day=1]	-2.2081702[a]	.52975571	1	.001	-3.9017728	-.5145675
	[Condition=1]*[Day=2]	-2.4591729[a]	.56789816	1	.001	-4.2747151	-.6436308
	[Condition=2]*[Day=0]	-3.9909765[a]	.44356432	1	.000	-5.4090295	-2.5729234
	[Condition=2]*[Day=1]	-.7010943	.54353579	1	1.000	-2.4387511	1.0365626
	[Condition=2]*[Day=2]	-.4319586	.56020401	1	1.000	-2.2229029	1.3589858
	[Condition=3]*[Day=0]	-2.3121451[a]	.44883600	1	.000	-3.7470515	-.8772388
	[Condition=3]*[Day=2]	.5288553	.58552867	1	1.000	-1.3430507	2.4007613
[Condition=3]*[Day=2]	[Condition=1]*[Day=0]	-4.6892455[a]	.49819224	1	.000	-6.2819413	-3.0965497
	[Condition=1]*[Day=1]	-2.7370255[a]	.57822101	1	.000	-4.5855693	-.8884817
	[Condition=1]*[Day=2]	-2.9880282[a]	.61335695	1	.000	-4.9488999	-1.0271566
	[Condition=2]*[Day=0]	-4.5198318[a]	.50044753	1	.000	-6.1197376	-2.9199259
	[Condition=2]*[Day=1]	-1.2299496	.59087189	1	1.000	-3.1189376	.6590385
	[Condition=2]*[Day=2]	-.9608139	.60624002	1	1.000	-2.8989330	.9773053
	[Condition=3]*[Day=0]	-2.8410005[a]	.50512591	1	.000	-4.4558628	-1.2261381
	[Condition=3]*[Day=1]	-.5288553	.58552867	1	1.000	-2.4007613	1.3430507

Pairwise comparisons of estimated marginal means based on the original scale of dependent variable NormRelengthVel

a. The mean difference is significant at the .05 level.

Overall Test Results

Wald Chi-Square	df	Sig.
235.299	8	.000

The Wald chi-square tests the effect of Condition*Day. This test is based on the linearly independent pairwise comparisons among the estimated marginal means.
